# Helical fused 1,2:8,9-dibenzozethrene oligomers with up to 201° end-to-end twist: “one-pot” synthesis and chiral resolution†

**DOI:** 10.1039/d3sc02285d

**Published:** 2023-06-23

**Authors:** Zhitao Sun, Wei Fan, Yi Han, Wei Yuan, Yong Ni, Jinyi Wang, Haipeng Wei, Yanli Zhao, Zhe Sun, Jishan Wu

**Affiliations:** a Joint School of National University of Singapore and Tianjin University, International Campus of Tianjin University Binhai New City Fuzhou 350507 China; b Department of Chemistry, National University of Singapore 3 Science Drive 3 117543 Singapore chmwuj@nus.edu.sg; c School of Chemistry, Chemical Engineering and Biotechnology, Nanyang Technological University 637371 Singapore; d Institute of Molecular Plus, Department of Chemistry and Haihe Laboratory of Sustainable Chemical Transformations, Tianjin University Tianjin 300072 China zhesun@tju.edu.cn

## Abstract

Twisted polyarenes with persistent chirality are desirable but their synthesis has remained a challenge. In this study, we present a “one-pot” synthesis of 1,2:8,9-dibenzozethrene (DBZ) and its vertically fused dimers and trimers using nickel-catalyzed cyclo-oligomerization reactions. X-ray crystallographic analysis confirmed highly twisted helical structures that consist of equal parts left- and right-handed enantiomers. Notably, the end-to-end twist between the terminal anthracene units measured 66°, 130°, and 201° for the DBZ monomer, dimer, and trimer, respectively, setting a new record among twisted polyarenes. Furthermore, the chiral resolution by HPLC yielded two enantiomers for the fused DBZ dimer and trimer, both of which maintained stable configurations and showed absorption dissymmetry factors of around 0.008–0.009. Additionally, their optical and electrochemical properties were investigated, which exhibited a chain-length dependence.

## Abstract 2

Helical fused 1,2:8,9-dibenzozethrene oligomers with up to 201° end-to-end twist were synthesized by “one-pot” nickel-catalyzed cyclo-oligomerization. Their fundamental structures, electronic properties, and chiroptical properties were investigated.

## Graphical abstract

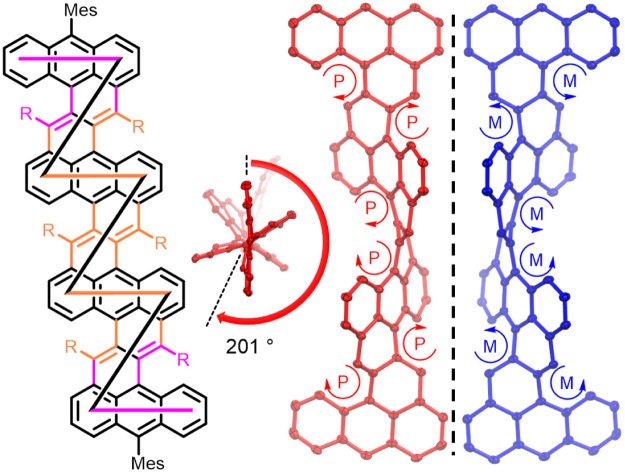

## Introduction

**Fig. 1:**
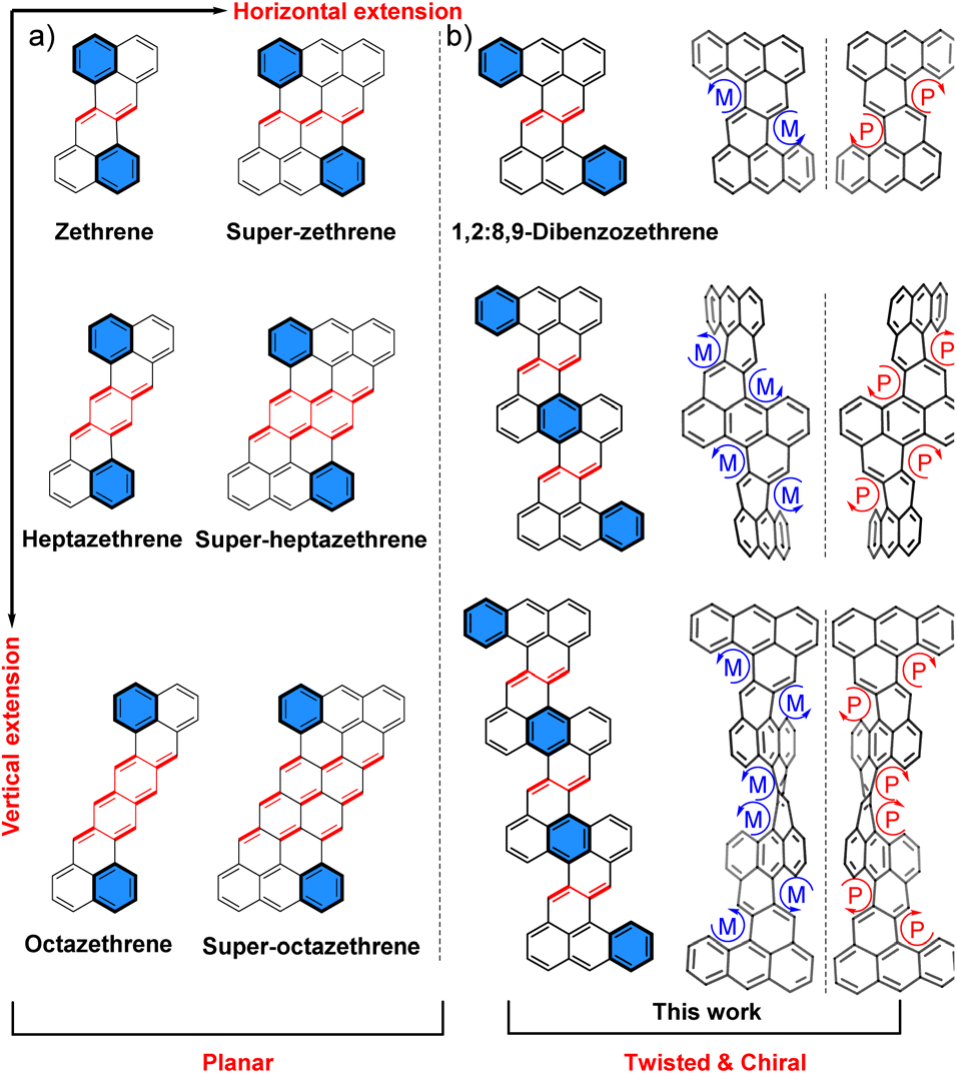
(a) Zethrene and horizontally and vertically extended zethrenes; (b) 1,2:8,9-dibenzozethrene (DBZ) and vertically fused DBZ oligomers reported in this work and their representative enantiomers.

For a long time, chemists have been interested in non-planar polyarenes that adopt a helical conformation due to their aesthetically pleasing structures and interesting chiroptical properties.^1^ However, their synthesis has remained a challenge. After years of effort, twistacenes^2^ and related structures^3^ have reached a record end-to-end twist of 184°,^4^ but most of them have a low racemization barrier, which limits their chiral resolution. To date, only a limited number of twisted polyarenes with a stable helical configuration have been reported, and the key to achieving this is controlling the strain and conformation along the propagation helical axis.^5^ In this study, we report on the facile synthesis and chiral resolution of several vertically fused oligomers of 1,2:8,9-dibenzozethrene (DBZ) (Fig. 1), which possess a stable helical configuration that is either left- or right-handed. Notably, the end-to-end twist in the fused trimer measured as high as 201°, which sets a new record among twisted polyarenes.

Zethrene, a Z-shaped polyarene with a “fixed” 1,4-butadiene moiety between its two terminal naphthalene units (Fig. 1a), was first synthesized in 1955.^6^ Theoretically, horizontally and vertically extended zethrenes (Fig. 1a) were predicted to show open-shell diradical character due to the recovery of aromaticity of the central quinoidal units in the diradical forms.^7^ In recent years, experimental evidence has supported this prediction by us and others.^8–10^ However, with an extension of the zethrene skeleton, the diradical character increases, which presents practical challenges for synthesis, and the obtained molecules usually have a planar or nearly planar geometry. On the other hand, DBZ derivatives with aryl substitution at the bay region exhibit a highly twisted conformation due to the strong steric repulsion between the aryl groups and the terminal anthracene units.^9^ In addition, the parent DBZ was calculated to have a small diradical character (*y*_0_ = 0.124 at UCAM-B3LYP/6-31G* level of theory) because only one aromatic sextet ring can be gained from the closed-shell to the diradical form. In this context, we hypothesized that vertically fused DBZ oligomers (Fig. 1b) with substituents at the cove regions could exhibit a stable helical configuration with reasonable chemical stability. To our delight, the fused DBZ dimer and trimer possess a left-handed or right-handed helical structure, and both enantiomers can be resolved by chiral HPLC at room temperature due to their high configuration stability.

## Result and discussion

### Design and synthesis

**Scheme 1:**
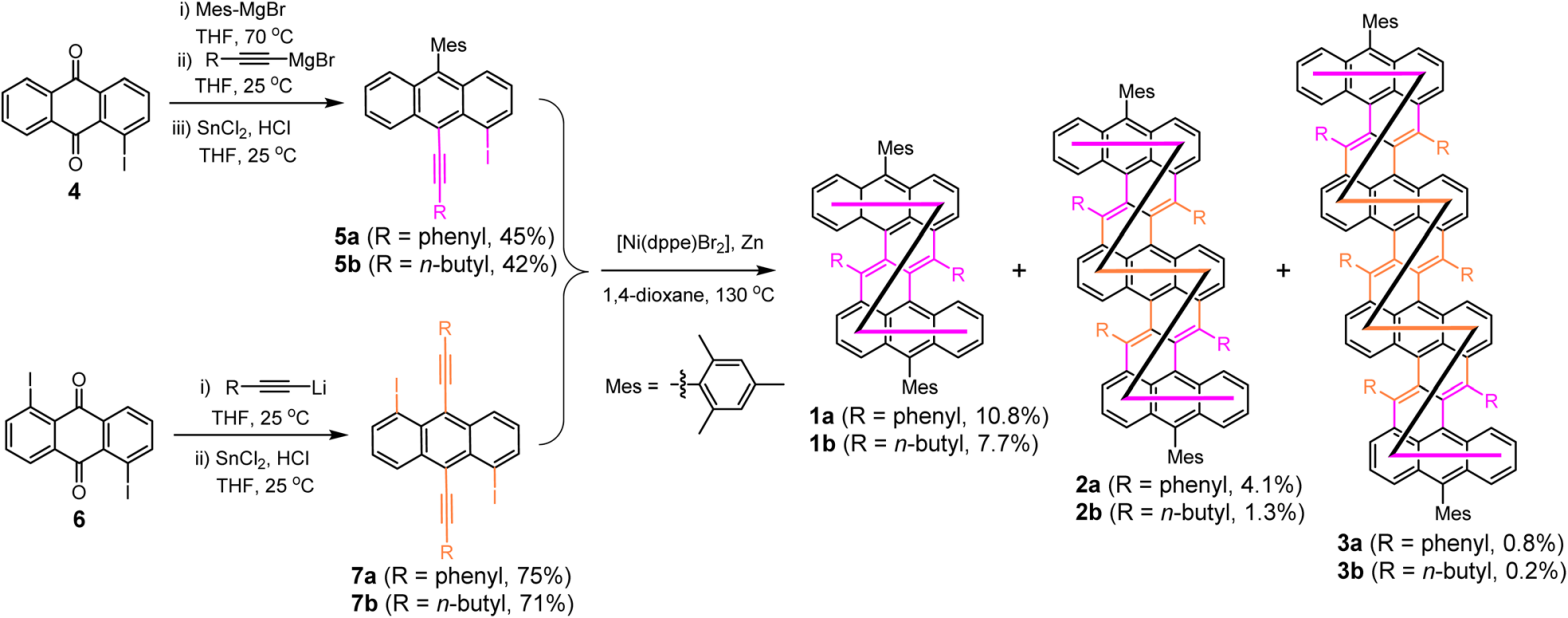
Synthetic routes of the fused 1,2:8,9-dibenzozethrene oligomers.

Nickel-catalyzed cyclodimerization reacton^10^ was used as the key reaction for the “one-pot” synthesis of the fused DBZ oligomers (1a/b–3a/b, Scheme 1). Bulky mesityl groups are attached onto the *meso*-positions of the terminal anthracene units to kinetically stabilize the molecules, whereas the phenyl or *n*-butyl substituents at the cove region induce steric congestion and help to form a stable helical structure. The key intermediates 5a/b and 7a/b containing phenylethynyl or 1-hexynyl units were first synthesized from 1-iodo-9,10-anthraquinone (4) and 1,5-diiodo-9,10-anthraquinone (6), respectively, through stepwise (for 5a/b) or one-pot (for 7a/b) nucleophilic addition with Grignard reagent or organolithium reagent, followed by reductive dehydroxylation reaction. Initially, the 1-hexynyl substituted building blocks 5b and 7b were subjected to the nickel-catalyzed cyclo-oligomerization, which afforded the DBZ derivative 1b and the fused dimer 2b and trimer 3b (R = *n*-butyl) in 7.7%, 1.3%, and 0.2% yield, respectively, after separation by preparative gel permeation chromatography (GPC). The low yields provoked us to use the building blocks having phenylethynyl groups, which gave the phenyl substituted DBZ 1a and fused oligomers 2a and 3a in improved yields of 10.8%, 4.1%, and 0.8%, respectively. A trace amount of the fused DBZ tetramer (*n* = 4) was also detected by MALDI-TOF mass spectrometry analysis of the crude product (Fig. S1†), but the isolation of this compound was unsuccessful. Despite the low yield of the trimer, the reaction was conducted on a relatively substantial scale, thus the reproducibility of the reaction is satisfactory. The structures of the isolated fused DBZ oligomers were confirmed by X-ray crystallographic analysis (for 1a, 1b, 2a, and 3b only, see below and ESI†), NMR, and high-resolution mass spectrometry. All the NMR peaks were assigned by 2D COSY and NOESY NMR techniques aided by gauge-independent atomic orbital (GIAO) calculations (see ESI†).

### X-ray crystallographic analysis and electronic structures

**Fig. 2:**
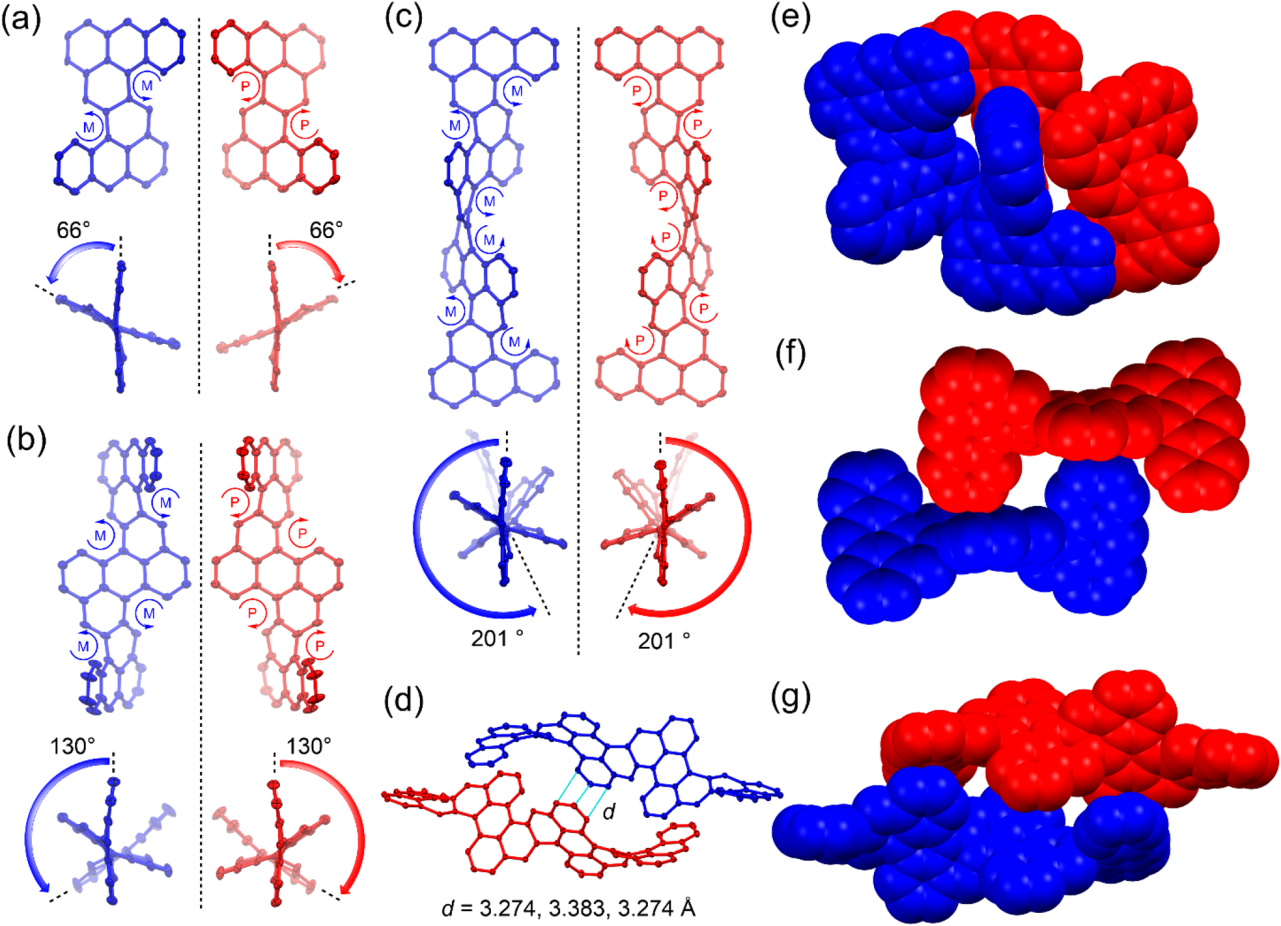
X-ray crystallographic structures of 1a, 2a and 3b. (a–c) Top view and side view of the crystal structure (backbone only) of 1a, 2a, and 3b, showing 50% probability thermal ellipsoids. (d) Short contacts between the enantiomers of 3b. (e–g) Illustration of the regular packing pattern of 1a, 2a, and 3b.

Single crystals of 1a, 2a, and 3b were grown by slowly diffusing methanol into their solutions in dichloromethane (DCM), toluene, and toluene, respectively. X-ray crystallography analysis revealed that all three molecules exhibit a highly twisted helical structure with the co-existence of left-handed and right-handed helix in a 1 : 1 ratio. A large end-to-end twist was observed between the two terminal anthracene units, with 66° for 1a, 130° for 2a, and 201° for 3b (Fig. 2a–c). This highly distorted conformation is attributed to the steric hindrance at the cove region induced by the repulsion between the substituents and nearby benzene rings. The backbone of 3b makes more than half a turn, representing the most twisted polyarene reported to date. Single crystals of 3a exhibited weak diffraction, and its structure was optimized using density functional theory (DFT) at the B3LYP/6-31G(d,p) level of theory, which exhibited a similar twisted structure to 3b. The splay angles between neighboring anthracene mean planes were found to be 66° for 1a, 64°/65° for 2a, and 66°/68°/63° for 3b. In all cases, the steric congestion at the cove regions made the molecules inherently chiral. All the [4]helicene subunits had the same (*M*) or (*P*) configuration in the left-handed or right-handed enantiomer, respectively. In the solid state, the two enantiomers of 1a or 2a were packed into 3D structures primarily through intermolecular [C–H⋯π] interactions (Fig. 2e and f). For 3b, the (*P*,*P*,*P*,*P*,*P*,*P*)- and (*M*,*M*,*M*,*M*,*M*,*M*)- enantiomers were paired through π–π stacking with short distances of 3.274, 3.383 and 3.274 Å, and additional [C–H⋯π] interactions (Fig. 2d and g).

The bond lengths between the butadiene moieties and adjacent anthracene units for 1a, 2a, and 3b are in the range of 1.474–1.480 Å and 1.333–1.378 Å (Fig. S28†). This indicates a “fixed” butadiene character. The C–C bonds linking these butadiene moieties to the nearby anthracene units are also relatively long (1.451–1.472 Å) (Fig. S28†), which suggests weak π-conjugation between the anthracene units. This weak coupling is presumably due to the highly twisted structures of the molecules. Harmonic oscillator model of aromaticity (HOMA)^11^ calculations based on the experimental bond lengths show that the naphthalene moiety linking the neighbouring anthracene units is nearly non-aromatic, with small HOMA values (0.04–0.11), while the anthracene units are aromatic, with HOMA values in the range of 0.58–0.79 (Fig. S28†). This is further supported by the large negative nuclear independent chemical shift (NICS)^12^ values in the anthracene units (−19.2 to −28.3 ppm) but positive NICS values (5.3–6.2 ppm) in the linking naphthalene units (Fig. S28†). In addition, the calculated anisotropy of the induced current density (ACID)^13^ plots show that the diatropic ring currents mainly circulate in each anthracene unit, with few in naphthalene bridges (Fig. S29†). Due to the twisted structure, all these molecules exhibit a closed-shell ground state and nearly zero diradical character, as determined by spin-unrestricted DFT calculations. Indeed, sharp NMR spectra were observed for the solution of 3a, even at elevated temperatures (Fig. S4†).

### Optical and electrochemical properties

**Fig. 3:**
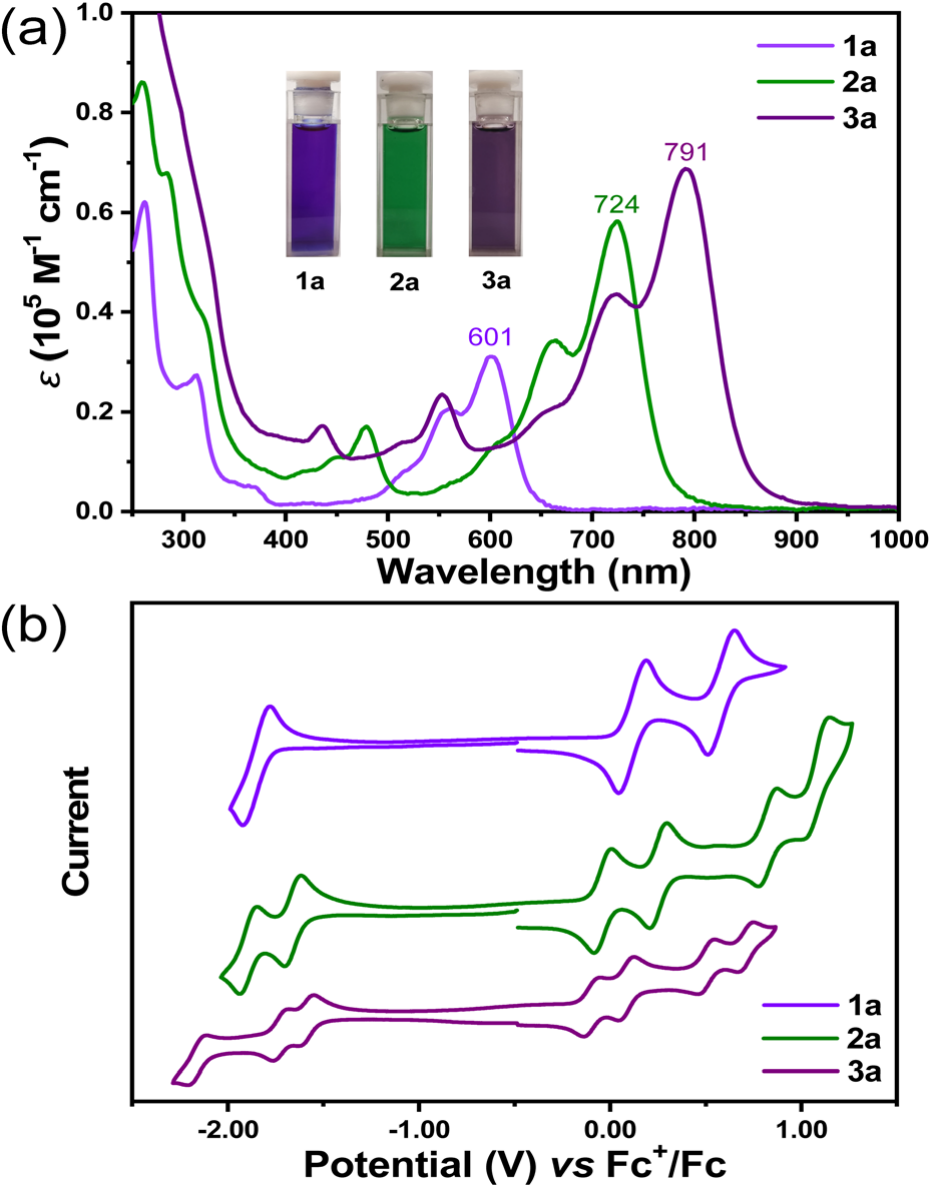
(a) UV-vis spectra of 1a, 2a and 3a in dichloromethane (1 × 10^−5^ M), inset are the photos of their solutions. (b) Cyclic voltammograms of 1a, 2a and 3a (V *vs.* Fc^+^/Fc, in 0.1 M nBu_4_N·PF_6_/dichloromethane).

**Fig. 4:**
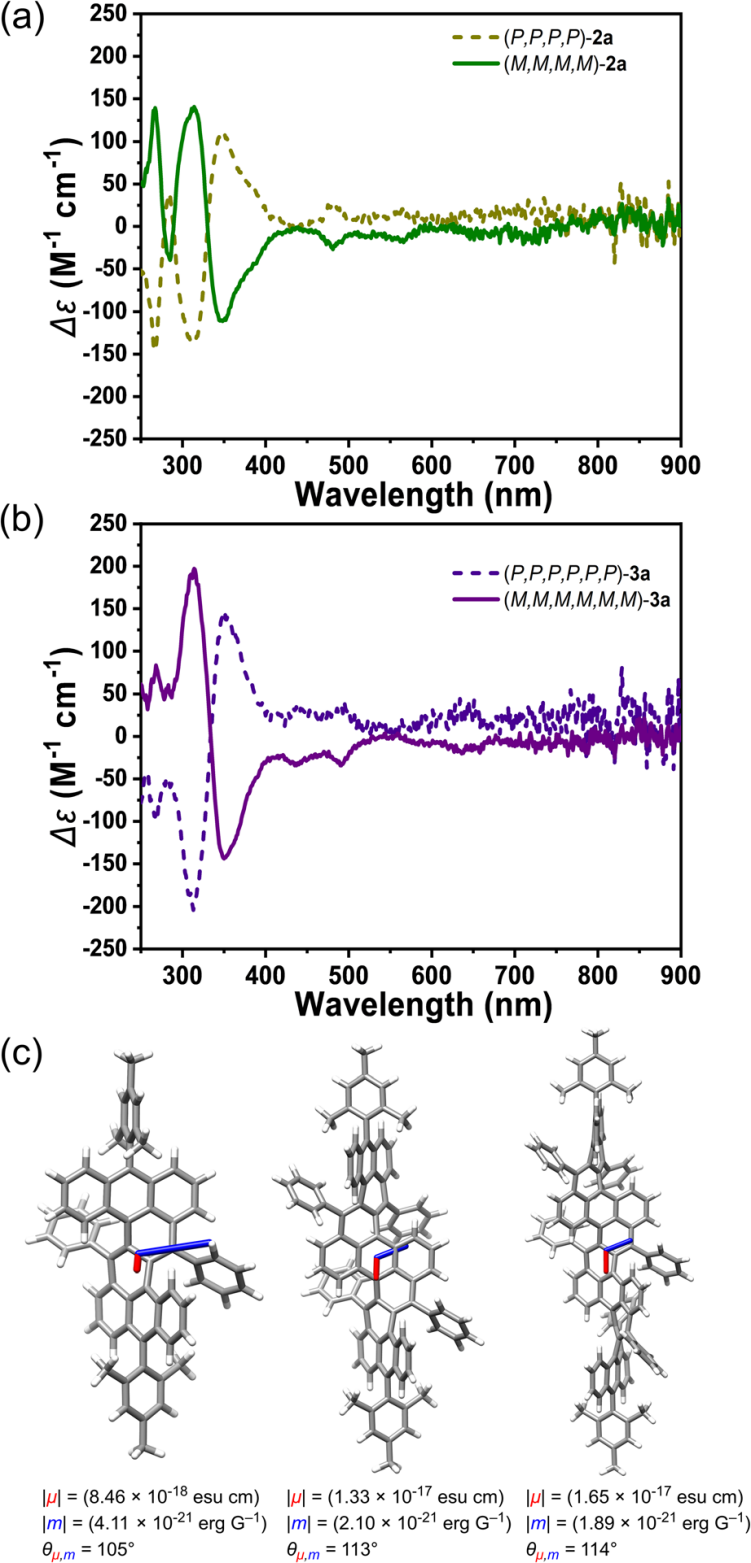
(a and b) CD spectra of (*P*,*P*,*P*,*P*)-2a, (*M*,*M*,*M*,*M*)-2a, (*P*,*P*,*P*,*P*,*P*,*P*)-3a, (*M*,*M*,*M*,*M*,*M*,*M*)-3a measured in dichloromethane at 25 °C with different concentrations (*c* = 1.34 × 10^−5^ M, 1.84 × 10^−5^ M, 6.93 × 10^−6^ M, 1.87 × 10^−5^ M, respectively). (c) Calculated transition dipole moments for S_0_ → S_1_ electronic transitions in (*P*,*P*)-1a, (*P*,*P*,*P*,*P*)-2a, (*P*,*P*,*P*,*P*,*P*,*P*)-3a. The electric transition moments (*μ*) are shown in red, and the magnetic transition moments (*m*) are shown in blue. The length of the *μ* vector is minified by 100 times when the length of the m vector is amplified by 100 times for clarity.

Compounds 1a, 2a, and 3a were dissolved in DCM to yield violet-, green-, and purple-colored solutions, respectively (Fig. 3a). In solution, all three molecules display well-resolved absorption spectra in solution, with the maximum absorption wavelength (*λ*_max_) at 601 nm for 1a (molar absorption coefficient *ε* = 31 000 M^−1^ cm^−1^), 724 nm for 2a (*ε* = 58 200 M^−1^ cm^−1^) and 791 nm for 3a (*ε* = 68 800 M^−1^ cm^−1^) (Fig. 3a). According to time-dependent (TD) DFT calculations, the most intense long-wavelength absorption band was attributed to the HOMO → LUMO electron transition in all cases. Despite the twisted structure, HOMO and LUMO coefficients were found to be distributed along the entire π-conjugated backbones (Fig. S22, S24 and S26†). The calculated electric transition moments (*μ*) for the S_0_–S_1_ transition were aligned along the helical propagation axis in all three molecules (Fig. 4c), and with the extension of the molecular length, the values of *μ* also increased, resulting in increased *ε* values. The optical energy gaps (*E*^Opt^_g_) were determined to be 1.86, 1.49, and 1.34 eV for 1a, 2a, and 3a, respectively, from the onset of the lowest energy absorption band. Compounds 1b, 2b, and 3b displayed similar absorption spectrum with major peaks slightly hypochromic shifted, presumably due to lack of π-conjugation between backbone and *n*-butyl substituents (Fig. S2†). No emission was observed for all six final compounds.

The electrochemical properties of 1a, 2a, and 3a in DCM were investigated using cyclic voltammetry and differential pulse voltammetry measurements (Fig. 3b and S3†). 1a displayed amphoteric redox behavior with three reversible redox waves, whereas 2a and 3a showed six and seven reversible redox waves, respectively, suggesting reduced Coulomb repulsion between the charges in the more extended conjugated backbones. Compound 1a exhibited two reversible oxidation waves with half-wave potential (*E*^ox^_1/2_) at 0.11 and 0.57 V and one reversible reduction wave with *E*^red^_1/2_ at −1.83, whereas for 2a, four sets of reversible oxidations with *E*^ox^_1/2_ at −0.05, 0.24, 0.82 and 1.07 V and two sets of reduction waves with *E*^red^_1/2_ at −1.66 and 1.90 V were observed. Compound 3a showed four sets of reversible oxidations with *E*^ox^_1/2_ at −0.11, 0.07, 0.50 and 0.70 V and three sets of reduction waves with *E*^red^_1/2_ at −1.60, −1.74 and −2.17 V. The HOMO/LUMO energy levels were estimated from the onset of the first oxidation/reduction to be −4.81/−3.02, −4.66/−3.23, and −4.57/−3.27 eV for 1a, 2a, and 3a, respectively. Therefore, extension of the molecular length slightly lifted the HOMO energy level and lowered the LUMO energy level. Accordingly, a convergence of the electrochemical energy gap (*E*^EC^_g_) was observed, with values of 1.79, 1.43, and 1.30 eV for 1a, 2a, and 3a, respectively, which is consistent with optical energy gap.

### Chiral properties

Chiral resolution of the racemic mixture was conducted for compounds 1a, 2a, and 3a by recycling preparative HPLC over a chiral phase (COSMOSIL Cholester) (Fig. S15–17†). Using acetone as an eluent, we separated the enantiomers of 2a after nine cycles, and confirmed the results with analytical chiral-phase HPLC and circular dichroism (CD) measurements. The enantiomers of 3a were separated using acetone/THF (9/1, v/v) as an eluent. However, we were not able to separate the enantiomers of 1a under a similar condition. The CD spectra of the enantiomers of 2a displayed a complete mirror image (Fig. 4a), and similar spectra were found for 3a with higher molar absorption coefficient at major peaks (Fig. 4b). The absolute configurations was estimated as (*P*,*P*,*P*,*P*)-2a, (*M*,*M*,*M*,*M*)-2a, (*P*,*P*,*P*,*P*,*P*,*P*)-3a and (*M*,*M*,*M*,*M*,*M*,*M*)-3a based on experimental and theoretical CD spectra (Fig. S18†). Negative cotton effects (CEs) were mainly localized between 250–330 nm for (*P*)-2a and (*P*)-3a, while weak positive CEs were even found beyond 600 nm for (*P*)-2a and (*P*)-3a. Moderate absorption dissymmetry factors |*g*_abs_| up to 0.008–0.009 at 354 nm and 0.008 at 362 nm were determined for 2a and 3a, respectively (Fig. S19†). The |*g*_abs_| values at the S_0_–S_1_ transitions are substantially low in both cases, which can be explained by nearly orthogonal arrangement of the electric transition moment and magnetic transition moment (*m*) (Fig. 4c).^14^ Thermal racemization of 2a was investigated by HPLC analysis of (*M*,*M*,*M*,*M*)-2a after heating in degassed toluene at 100 °C, which revealed a transformation from (*M*)-2a to (*P*)-2a with a half-life time (*t*^1/2^) of 4.3 h (Fig. S20†). The racemization barrier of Δ*G*^‡^ (373 K) was estimated to be 30 kcal mol^−1^ by using the Eyring equation.

## Conclusions

In summary, highly twisted 1,2:8,9-dibenzozethrene and its vertically fused dimers and trimers were synthesized by a “one-pot” nickel-catalyzed cyclo-oligomerization reaction. The steric congestion at the cove regions of the backbones results in a helical structure. Notably, the fused DBZ trimers exhibit an end-to-end twist of about 201°, which sets a new record among twisted polyarenes. The fused DBZ dimer and trimer also display high configuration stabilities, and chiral resolution of their enantiomers was successful for both. This study demonstrated an efficient way to access highly twisted polyarenes with separatable enantiomers with either left-handed or right-handed helicity.

## Author contributions

J. W. and Z. S. supervised the project. Z.-T. S. synthesized and characterized the compounds. W. F., Y. H., Y. N., and H. W. performed crystallographic analysis. J. Y. W. performed HPLC analysis. W. Y. and Y. Z. performed circular dichroism measurements. All authors discussed the results and contributed to the manuscript writing.

## Conflicts of interest

There are no conflicts to declare.

## Supplementary Material

SC-014-D3SC02285D-s001

SC-014-D3SC02285D-s002

SC-014-D3SC02285D-s003

SC-014-D3SC02285D-s004

SC-014-D3SC02285D-s005

SC-014-D3SC02285D-s006

SC-014-D3SC02285D-s007

SC-014-D3SC02285D-s008

SC-014-D3SC02285D-s009

SC-014-D3SC02285D-s010
