# Supplementary material for: Helical fused 1,2:8,9-dibenzozethrene oligomers with up to 201° end-to-end twist: “one-pot” synthesis and chiral resolution
Source: Chem Sci. 2023 Jun 23;14(29):7922–7. doi: 10.1039/d3sc02285d (PMC10370577; doi:10.1039/d3sc02285d)
Supplement: SC-014-D3SC02285D-s010 [file SC-014-D3SC02285D-s010.pdf]

Electronic Supplementary Material (ESI) for Chemical Science.
This journal is © The Royal Society of Chemistry 2023

Electronic Supplementary Information (ESI)

Table of Contents

1. Experimental section ········ S2
   1.1 General ········ S2
   1.2 Synthetic procedures and characterization data ········ S3
2. Additional spectra ········ S12
3. Thermal stability ········ S21
4. DFT calculations ········ S23
5. X-ray crystallographic data ········ S36
6. $^{1}H/^{13}C$-NMR and HR mass spectra of new compounds ········ S41
7. References ········ S58

1. Experimental Section

1.1. General

All reagents and starting materials were obtained from commercial suppliers and used without further purification unless otherwise noted. Compounds **4** and **6** were synthesized according to previous literatures.[1] Anhydrous THF were distilled from sodium-benzophenone immediately prior to use. All reaction conditions dealing with air- and moisture sensitive compounds were carried out in a dry reaction vessel under an argon atmosphere. The ^{1}H NMR and ^{13}C NMR spectra were recorded in deuterated solvents on Bruker DPX400/DPX500 NMR spectrometer. All chemical shifts are quoted in ppm, relative to tetramethyl silane, using the residual solvent peak as a reference standard. The following abbreviations were used to explain the multiplicities: s = singlet, d = doublet, m = multiplet, t = triplet. Atmospheric Pressure Chemical Ionization Mass Spectrometry (APCI MS) measurements were performed on a Finnigan TSQ 7000 triple stage quadrupole mass spectrometer. UV-vis-NIR absorption spectra were recorded on a Shimadzu UV-3600 spectrophotometer. Cyclic voltammetry measurements were performed in dry DCM on a CHI 620C electrochemical analyzer with a three-electrode cell, using 0.1 M n-Bu_4NPF_6 as supporting electrolyte, AgCl/Ag as reference electrode, gold disk as working electrode, Pt wire as counter electrode, with a scan rate of 50 mV/s. The potential was externally calibrated against the ferrocene/ferrocenium (Fc/Fc^{+}) couple. The HOMO and LUMO energy levels were calculated according to the equations: HOMO = $-(4.8 + E_{ox}^{onset})$ eV and LUMO = $-(4.8 + E_{red}^{onset})$ eV, where the E_{ox}^{onset} and E_{red}^{onset} are the onset potentials of the first oxidative and reductive redox wave, respectively. Chiral HPLC analysis was conducted on a Shimadzu Prominence 2000 instrument. CD spectra were obtained on JASCO J-1500 at 25 °C.

1.2. Synthetic procedures and characterization data

Synthesis of compound 4′:

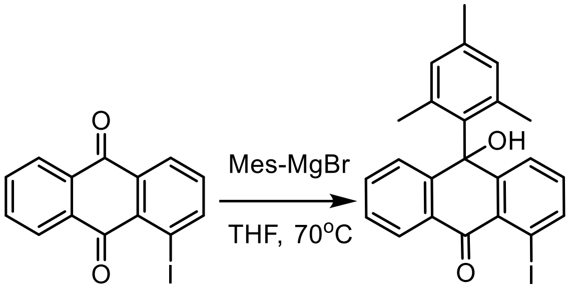

4 **4'**

Compound 1-iodoanthraquinone (**4**, 3.0 g, 9.0 mmol) was dissolved in 200 mL of anhydrous THF, and then mesitylmagnesium bromide (12.0 ml, 12.0 mmol, 1M in diethyl ether) was added dropwise. After the solution was stirred at 70 °C for 12 hours, the reaction was quenched with aq. NH_4Cl. The mixture was treated with 100 mL water and extracted with DCM. The combined organic layer was dried over $MgSO_4$ and the solvent was removed under vacuum. The crude product was purified by silica gel column chromatography (hexane/DCM = 1/1) to give compound **4′** as a yellow solid (2.6 g, 63.7%). ^{1}H NMR (500 MHz, 298 K, CD_2Cl_2): δ ppm 8.25-8.22 (m, 1H), 8.14 (dd, ^3J = 7.8, ^4J = 1.1 Hz, 1H), 7.53-7.47 (m, 2H), 7.26 (dd, ^3J = 7.8, ^4J = 1.1 Hz, 1H), 7.16-7.12 (m, 1H), 7.09 (t, J = 7.8 Hz, 1H), 6.85 (s, 2H), 2.45 (s, 1H), 2.27 (s, 3H), 2.05 (s, 6H). ^{13}C NMR (100 MHz, 298 K, CD_2Cl_2): δ ppm 182.5, 150.9, 146.6, 143.6, 137.5, 137.5, 137.4, 134.5, 134.4, 132.2, 130.5, 129.5, 129.2, 129.0, 128.2, 127.6, 93.5, 77.9, 23.8, 20.7. HRMS analysis (APCI, *m*/*z*) [$(M+H)^+$] calcd for $C_{23}H_{20}O_2I$: 455.0502, found 455.0509 (error: 1.54 ppm).

Synthesis of compound 5a:

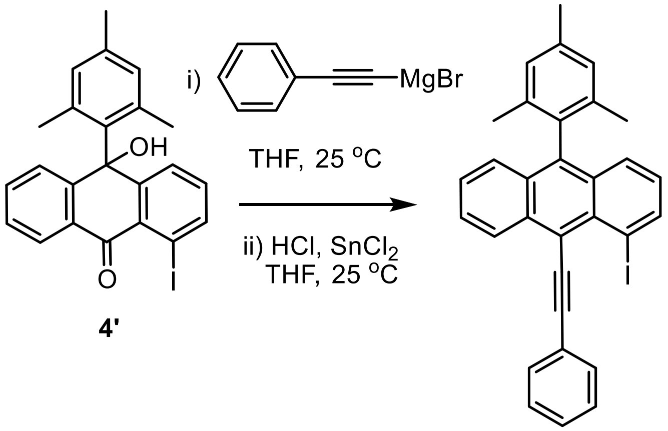

4' **5a**

Phenylacetylene (3.27 g, 32.0 mmol) was dissolved in 200 mL of anhydrous THF and the solution was cooled down to 0 °C with an ice bath. Ethylmagnesium bromide (10.0 mL, 30.0 mmol, 3 M in diethyl ether) was added dropwise and the mixture was allowed to stir for 5 hours. Compound **4′** (4.54 g, 10 mmol) was then added dropwise to the freshly prepared Grignard reagent at 0 °C. The mixture was then slowly warmed up to room temperature and stirred for 14 hours. Subsequently, a solution of $SnCl_2$ (9.45 g, 50.0 mmol) in 40 mL of 3M HCl was added to the reaction mixture, and the solution was further stirred for 3 hours at room temperature. Afterwards, the mixture was treated with water (200 mL) and extracted with DCM. The combined organic solution was dried over $MgSO_4$ and the solvent was removed under vacuum. The crude product was purified by silica gel column chromatography (hexane/DCM = 9/1) to give compound **5a** as a yellow solid (3.7 g, 70.9%). ^{1}H NMR (500 MHz, 298 K, CD_2Cl_2): δ ppm 8.95 (d, J = 8.8 Hz, 1H), 8.40 (dd, ^3J = 7.0, ^4J = 1.2, 1H), 7.89-7.83 (m, 2H), 7.70-7.64 (m, 1H), 7.56 (dd, ^3J = 8.7, ^4J = 1.2 Hz, 1H), 7.51-7.41 (m, 5H), 7.10 (s, 2H), 6.94 (dd, ^1J = 8.7, ^2J = 7.0 Hz, 1H), 2.44 (s, 3H), 1.68 (s, 6H). ^{13}C NMR (100 MHz, 298 K, CD_2Cl_2): δ ppm 143.8, 139.1, 138.2, 137.5, 134.8, 134.6, 132.0, 131.1, 131.1, 130.9, 130.0, 129.1, 129.0, 128.8, 128.4, 127.9, 127.9, 127.1, 126.7, 126.7, 124.4, 119.0, 110.2, 93.5, 87.8, 21.4, 20.0. HRMS analysis (APCI, *m*/*z*) $[(M+H)^+]$ calcd for $C_{31}H_{24}I$: 523.0917, found 523.092 (error: 0.57 ppm).

Synthesis of compound 5b:

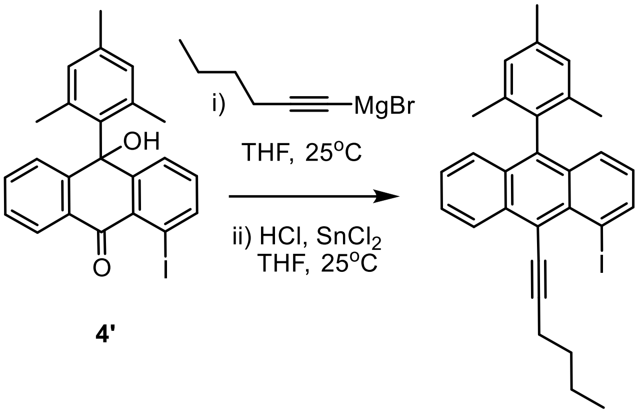

1-Hexyne (2.66 g, 32.0 mmol) was dissolved in 200 mL of anhydrous THF, and the solution was cooled down to 0 °C with an ice bath. Ethylmagnesium bromide (10.0 mL, 30.0 mmol, 3 M in diethyl ether) was added dropwise and the mixture was allowed to stir for 5 hours. Compound **4′** (4.54 g, 10 mmol) was then added dropwise to the freshly prepared Grignard reagent at 0 °C. The mixture was then slowly warmed up to room temperature and stirred for 14 hours. Subsequently, a solution of $SnCl_2$ (9.45 g, 50.0 mmol) in 40 mL of 3M HCl was added to the reaction mixture, and the solution was further stirred for 3 hours at room temperature. Afterwards, the mixture was treated with water (200 mL) and extracted with DCM. The combined organic solution was dried over $MgSO_4$ and the solvent was removed under vacuum. The crude product was purified by silica gel column chromatography (hexane/DCM = 9/1) to give compound **5b** as a yellow solid (3.3 g, 65.7%). ^{1}H NMR (500 MHz, 298 K, $CDCl_3$): δ ppm 8.81 (d, J = 8.8 Hz, 1H), 8.33 (dd, ^3J = 7.0, ^4J = 1.2 Hz, 1H), 7.61-7.55 (m, 1H), 7.50 (dd, ^3J = 8.7, ^4J = 1.2 Hz, 1H), 7.44-7.33 (m, 2H), 7.07 (s, 2H), 6.86 (dd, ^3J = 8.7, ^4J = 7.0 Hz, 1H), 2.78 (t, J = 7.2 Hz, 2H), 2.44 (s, 3H), 1.88-1.78 (m, 2H), 1.69-1.60 (m, 8H), 1.03 (t, J = 7.3 Hz, 3H). ^{13}C NMR (100 MHz, 298 K, $CDCl_3$): δ ppm 143.2, 137.6, 137.5, 137.4, 134.6, 134.5, 130.6, 130.5, 129.6, 128.5, 128.1, 128.0, 127.0, 126.6, 126.3, 126.2, 119.8, 112.7, 93.4, 78.3, 30.4, 22.7, 21.4, 21.1, 20.1, 13.9. HRMS analysis (APCI, *m*/*z*) [$(M+H)^+$] calcd for $C_{29}H_{28}I$: 503.123, found 503.1227 (error: 0.60 ppm).

Synthesis of compound 7a:

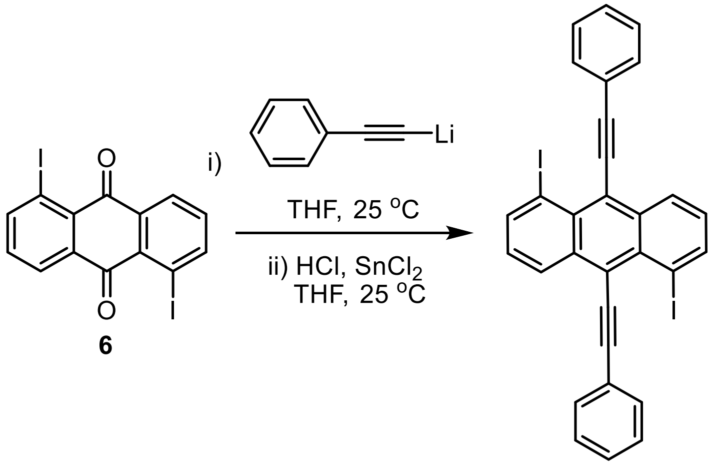

7a

Phenylacetylene (6.55 g, 64.0 mmol) was dissolved in 200 mL of anhydrous THF and the solution was cooled down to -78 °C with an acetone/dry ice bath. *n*-BuLi (30.0 mL, 60.0 mmol, 2 M in cyclohexane) was added dropwise and the mixture was allowed to stir for 3 hours. Then 1,5-diiodoanthraquinone (4.6 g, 10.0 mmol) was added dropwise to the freshly prepared lithium reagent at -78 °C. The mixture was then slowly warmed up to room temperature and stirred overnight. Subsequently, a solution of $SnCl_2$ (9.45 g, 50.0 mmol) in 40 mL of 3M HCl was added to the reaction mixture, and the solution was further stirred for 3 hours at room temperature. Afterwards, the mixture was poured into 200 mL of water and the solid was filtered and collected. The solid was washed by DCM until TLC showed only one spot, and then the residue was subjected to column chromatography on silica gel with DCM as eluent to give compound **7a** as a red solid (4.7 g, 74.6%). ^{1}H NMR (500 MHz, 298 K, CD_2Cl_2): δ ppm 9.00 (dd, ^3J = 8.8, ^4J = 1.1 Hz, 1H), 8.45 (dd, ^3J = 7.1, ^4J = 1.1 Hz, 1H), 7.85-7.80 (m, 2H), 7.51-7.43 (m, 3H), 7.28 (dd, ^3J = 8.8, ^4J = 7.1 Hz, 1H). ^{13}C NMR (125 MHz, 298 K, CD_2Cl_2): δ ppm 144.4, 135.3, 131.3, 131.2, 129.6, 129.2, 129.1, 128.2, 123.9, 121.3, 112.6, 92.9, 87.6. HRMS analysis (APCI, *m*/*z*) [$(M+H)^+$] calcd for $C_{30}H_{17}I_2$: 630.9414, found 630.9411 (error: 0.48 ppm).

Synthesis of compound 7b:

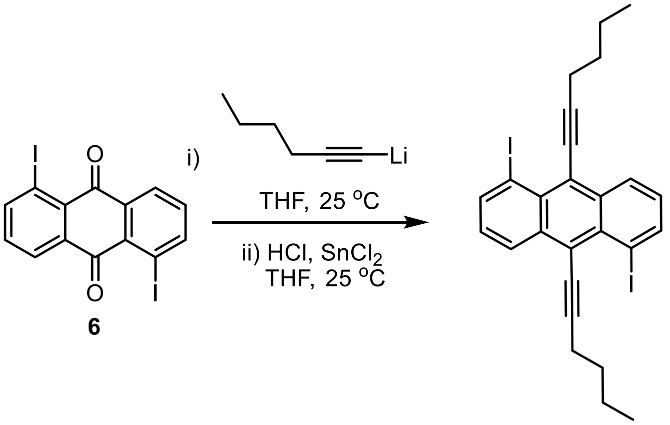

7b

1-Hexyne (5.32 g, 64 mmol) was dissolved in 200 mL of anhydrous THF and the solution was cooled down to -78 °C with an acetone/dry ice bath. *n*-BuLi (30.0 mL, 60.0 mmol, 2 M in cyclohexane) was added dropwise and the mixture was allowed to stir for 3 hours. Then 1,5-diiodoanthraquinone (4.6 g, 10.0 mmol) was added dropwise to the freshly prepared lithium reagent at -78 °C. The mixture was then slowly warmed up to room temperature and stirred overnight. Subsequently, a solution of $SnCl_2$ (9.45 g, 50.0 mmol) in 40 mL of 3M HCl was added to the reaction mixture, and the solution was further stirred for 3 hours at room temperature. Afterwards, the mixture was treated with water (200 mL) and extracted with DCM. The combined organic solution was dried over $MgSO_4$ and the solvent was removed under vacuum. The crude product was purified by silica gel column chromatography (hexane/DCM = 9/1) to give compound **7b** as an orange solid (4.2 g, 71.2%). ^{1}H NMR (500 MHz, 298K, CD_2Cl_2): δ ppm 8.84 (dd, ^3J = 8.8, ^4J = 1.2 Hz, 1H), 8.36 (dd, ^3J = 7.1, ^4J = 1.2 Hz, 1H), 7.17 (dd, ^3J = 8.8, ^4J = 7.1 Hz, 1H), 2.74 (t, J = 7.2 Hz, 2H), 1.83-1.74 (m, 2H), 1.63-1.54 (m, 2H), 1.00 (t, J = 7.4 Hz, 3H). ^{13}C NMR (125 MHz, 298 K, CD_2Cl_2): δ ppm 144.1, 135.3, 130.9, 129.5, 127.6, 121.3, 115.2, 92.7, 78.4, 30.5, 22.9, 21.3, 13.9. HRMS analysis (APCI, *m*/*z*) [$(M+H)^+$] calcd for $C_{26}H_{25}I_2$: 591.004, found 591.0036 (error: 0.68 ppm).

Synthesis of compounds 1a, 2a, and 3a:

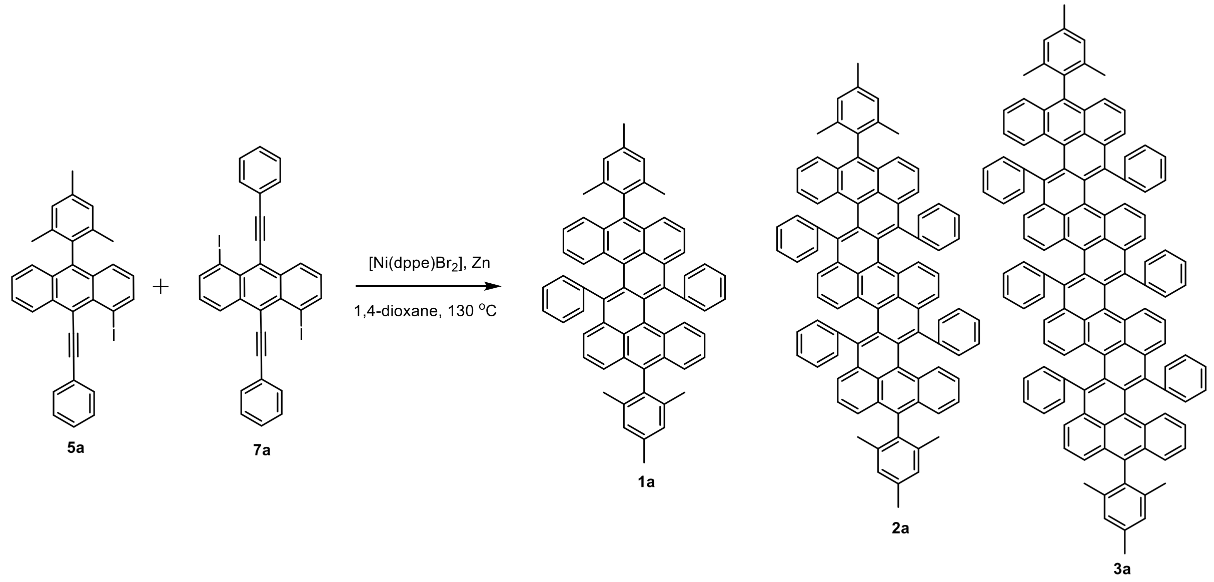

A mixture of **5a** (1.04 g, 2.0 mmol), **7a** (1.26 g, 2.0 mmol), $NiBr_2$(dppe) (93 mg, 0.15 mmol), Zn powder (392 mg, 6 mmol) and 1,4-dioxane (20 mL) in a 350-mL round bottom pressure vessel with PTFE bushing was sealed in glove box. The sealed vessel was kept in an oil bath at 130 °C for 16 h. After cooling to room temperature, the suspension was filtered through a 3 cm thick layer of Celite, and the Celite was rinsed with DCM. The solvents of the filtrate were removed under vacuum and the residue was first purified via silica gel column chromatography (DCM) and then further purified by preparative GPC using THF at a rate of 14 mL/min to afford crude compounds **1a**, **2a**, **3a**. Each crude product was then recrystallized to afford pure compound **1a** (85 mg, 10.8%) as a purple solid, **2a** (48 mg, 4.1%) as a green solid, and **3a** (12 mg, 0.8%) as a dark purple solid.

1a: ^{1}H NMR (500 MHz, 298 K, deacidified CD_2Cl_2): δ ppm 8.06 (d, J = 8.2 Hz, 1H), 7.65 (d, J = 7.8 Hz, 1H), 7.42-7.38 (m, 2H), 7.35-7.26 (m, 2H), 7.22 (d, J = 8.6 Hz, 1H), 7.15 (s, 1H), 7.12 (s, 1H), 7.07-7.01 (m, 1H), 7.00-6.95 (m, 1H), 6.94-6.88 (m, 1H), 6.54 (td, ^{3}J = 7.6, ^{4}J = 1.0 Hz, 1H), 6.15 (d, J = 7.7 Hz, 1H), 2.46 (s, 3H), 1.92 (s, 3H), 1.73 (s, 3H). ^{13}C NMR (125 MHz, 298 K, deacidified CD_2Cl_2): δ ppm 139.1, 138.3, 137.8, 137.7, 137.7, 136.0, 135.1, 133.9, 133.6,

131.9, 130.5, 129.2, 129.1, 128.9, 128.8 128.5, 128.3, 128.2, 128.1, 127.8, 126.9, 126.2, 126.0, 125.7, 125.4, 125.3, 123.6, 21.4, 20.2, 20.1. HRMS analysis (APCI, *m*/*z*) $[(M+H)^+]$ calcd for $C_{64}H_{47}$: 791.3672, found 791.3668 (error: 0.51 ppm). **2a:** ^{1}H NMR (500 MHz, 298K, deacidified CD_2Cl_2): δ ppm 7.92 (dd, ^3J = 11.7, ^4J = 8.6 Hz, 2H), 7.66 (d, *J* = 7.7 Hz, 1H), 7.46 (d, *J* = 7.7 Hz, 1H), 7.43-7.36 (m, 2H), 7.34-7.27 (m, 2H), 7.25-7.20 (m, 2H), 7.19-7.14 (m, 2H), 7.12 (s, 1H), 7.05-6.98 (m, 3H), 6.94 (t, *J* = 7.9 Hz, 1H), 6.88 (t, *J* = 7.5 Hz, 2H), 6.61-6.56 (m, 1H), 6.52 (td, ^1J = 7.6, ^2J = 1.4 Hz, 1H), 6.13 (d, *J* = 7.7 Hz, 1H), 2.47 (s, 3H), 1.94 (s, 3H), 1.74 (s, 3H). ^{13}C NMR (100 MHz, 298 K, deacidified CD_2Cl_2): δ ppm 139.2, 139.0, 138.6, 138.4, 137.8, 137.7, 137.7, 135.9, 135.1, 134.2, 133.8, 133.6, 132.7, 132.2, 131.8, 131.7, 130.5, 129.3, 129.1, 128.9, 128.8, 128.5, 128.4, 128.3, 128.2, 128.1, 128.1, 128.0, 127.9, 127.7, 127.6, 127.2, 126.8, 126.2, 126.1, 125.7, 125.6, 125.4, 125.2, 123.6, 123.6, 21.4, 20.3, 20.1. HRMS analysis (APCI, *m*/*z*) $[(M+H)^+]$ calcd for $C_{92}H_{63}$: 1167.4924, found 1167.4922 (error: 0.17 ppm). **3a:** ^{1}H NMR (500 MHz, 298K, THF-d_8): δ ppm 8.06 (d, *J* = 8.5 Hz, 1H), 7.91 (d, *J* = 8.6 Hz, 1H), 7.85 (d, *J* = 8.5 Hz, 1H), 7.74 (d, *J* = 7.7 Hz, 1H), 7.53 (d, *J* = 7.8 Hz, 1H), 7.45 (d, *J* = 7.8 Hz, 1H), 7.42-7.38 (m, 2H), 7.36-7.28 (m, 2H), 7.28-7.19 (m, 4H), 7.18-7.13 (m, 2H), 7.12 (s, 1H), 7.07-7.00 (m, 3H), 7.00-6.87 (m, 5H), 6.84 (t, *J* = 7.5 Hz, 1H), 6.66-6.61 (m, 1H), 6.59 (d, *J* = 7.5, 1.8 Hz, 1H), 6.49 (td, ^3J = 7.6, ^4J = 1.3 Hz, 1H), 6.09 (d, *J* = 7.9, 1.5 Hz, 1H), 2.44 (s, 3H), 1.92 (s, 3H), 1.75 (s, 3H). ^{13}C NMR (125 MHz, 298 K, THF-d_8): δ ppm 139.6, 139.4, 139.3, 139.1, 138.9, 138.8, 138.0, 137.9, 137.8, 136.0, 135.5, 134.5, 134.4, 134.2, 134.1, 133.9, 133.0, 133.0, 132.6, 132.3, 132.3, 132.2, 132.0, 130.9, 129.8, 129.4, 129.3, 129.1, 128.9, 128.8, 128.7, 128.7, 128.6, 128.5, 128.5, 128.4, 128.3, 128.2, 128.1, 128.0, 128.0, 127.7, 127.6, 127.2, 126.4, 126.2, 126.0, 125.9, 125.5, 125.5, 124.1, 123.9, 21.2, 20.1, 20.0. HRMS analysis (APCI, *m*/*z*) $[(M+H)^+]$ calcd for $C_{122}H_{79}$: 1543.6176, found 1543.6175 (error: 0.06 ppm).

Synthesis of compounds 1b, 2b, and 3b:

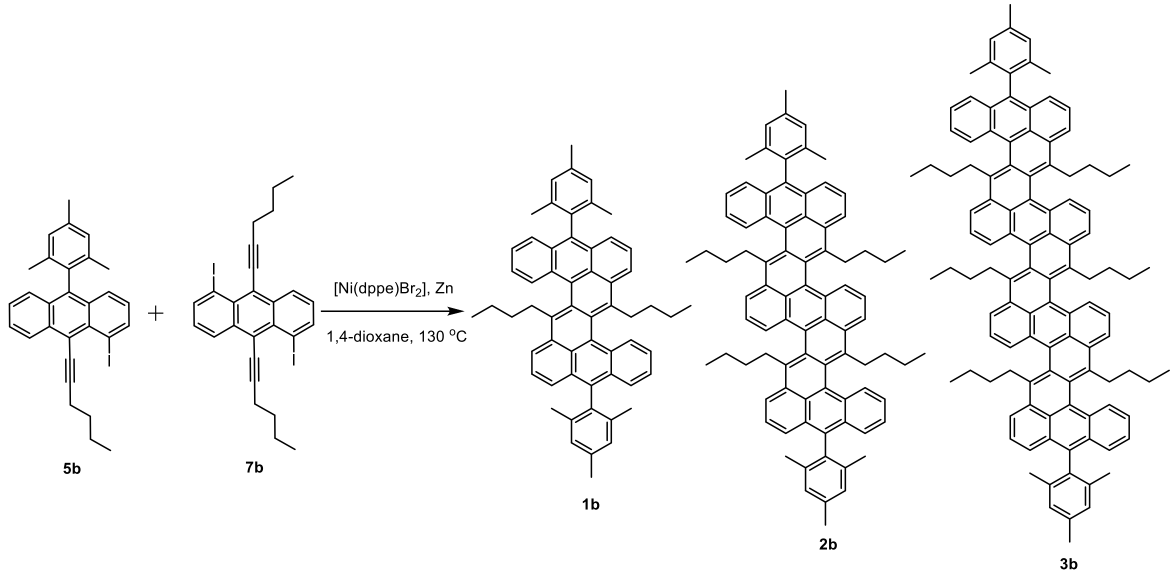

A mixture of **5b** (1.0 g, 2.0 mmol), **7b** (1.18 g, 2 mmol), $NiBr_2$(dppe) (93 mg, 0.15 mmol), Zn powder (392 mg, 6.0 mmol) and 1,4-dioxane (20 mL) in a 350-mL round bottom pressure vessel with PTFE bushing was sealed in glove box. The sealed vessel was kept in an oil bath at 130 °C for 16 h. After cooling to room temperature, the suspension was filtered through a 3 cm thick layer of Celite, and the Celite was rinsed with DCM. The solvents of the filtrate were removed under reduced pressure and the residue was first purified via silica gel column chromatography (DCM) and then further purified by preparative GPC using THF at a rate of 14 mL/min to afford crude compounds **1b**, **2b**, **3b**. Each crude product was then recrystallized to afford compound **1b** (60 mg, 7.7%) as a purple solid, **2b** (15 mg, 1.3%) as a green solid, and **3b** (3 mg, 0.2%) as a dark purple solid.

1b: ^{1}H NMR (500 MHz, 298 K, deacidified $CDCl_3$): δ ppm 8.23 (d, *J* = 8.4 Hz, 1H), 7.75 (d, *J* = 6.5 Hz, 1H), 7.51 (d, *J* = 8.3 Hz, 1H), 7.45-7.35 (m, 3H), 7.34-7.30 (m, 1H), 7.15 (s, 1H), 7.12 (s, 1H), 2.86-2.77 (m, 1H), 2.76-2.67 (m, 1H), 2.48 (s, 3H), 1.89 (s, 3H), 1.78 (s, 3H), 1.54-1.47 (m, 1H), 1.39-1.30 (m, 1H), 0.89-0.77 (m, 1H), 0.70-0.61 (m, 1H), 0.27 (t, *J* = 7.3 Hz, 3H). ^{13}C

NMR (125 MHz, 298 K, deacidified $CDCl_3$): δ ppm 138.0, 137.7, 137.3, 135.3, 135.0, 135.0, 134.3, 132.7, 130.3, 129.5, 129.3, 129.0, 128.5, 128.4, 128.3, 127.4, 126.3, 126.1, 125.7, 125.5, 125.2, 121.3, 32.0, 28.3, 21.8, 21.4, 20.3, 20.1, 13.5. HRMS analysis (APCI, *m/z*) $[(M+H)^+]$ calcd for $C_{58}H_{55}$: 751.4298, found 751.43 (error: 0.27 ppm). **2b:** ^1H NMR (500 MHz, 298K, THF-d_8): δ ppm 8.14 (d, *J* = 8.4 Hz, 1H), 8.07 (d, *J* = 8.6 Hz, 1H), 7.85 (t, *J* = 7.3 Hz, 2H), 7.55-7.49 (m, 2H), 7.48-7.45 (m, 1H), 7.42-7.36 (m, 2H), 7.35-7.31 (m, 1H), 7.17 (s, 1H), 7.14 (s, 1H), 3.10-3.01 (m, 1H), 2.93-2.78 (m, 3H), 2.47 (s, 3H), 1.86 (s, 3H), 1.79 (s, 3H), 1.62-1.51 (m, 3H), 1.46-1.36 (m, 1H), 1.07-0.94 (m, 1H), 0.93-0.68 (m, 3H), 0.54 (t, *J* = 7.3 Hz, 3H), 0.28 (t, *J* = 7.4 Hz,3H). ^{13}C NMR (125 MHz, 298 K, THF-d_8): δ ppm 137.4, 137.1, 135.2, 134.9, 134.9, 134.7, 134.3, 133.0, 132.5, 132.3, 130.2, 129.6, 129.2, 128.9, 128.6, 128.4, 128.3, 128.2, 127.9, 127.6, 127.3, 126.7, 126.4, 126.1, 125.8, 125.6, 125.5, 124.8, 121.9, 121.4, 32.3, 31.9, 29.7, 28.7, 28.1, 22.0, 21.4, 20.4, 19.3, 19.3, 13.3, 12.7. HRMS analysis (APCI, *m/z*) $[(M+H)^+]$ calcd for $C_{84}H_{79}$: 1087.6176, found 1087.6184 (error: 0.74 ppm). **3b:** ^1H NMR (500 MHz, 298K, THF-d_8): δ ppm 8.14 (d, *J* = 8.5 Hz, 1H), 8.09 (d, *J* = 8.7 Hz, 1H), 8.00 (d, *J* = 8.7 Hz, 1H), 7.92-7.84 (m, 3H), 7.58-7.50 (m, 3H), 7.49-7.45 (m, 1H), 7.42-7.37 (m, 2H), 7.36-7.32 (m, 1H), 7.17 (s, 1H), 7.15 (s, 1H), 3.15-3.03 (m, 2H), 2.98-2.80 (m, 4H), 2.47 (s, 3H), 1.87 (s, 3H), 1.80 (s, 3H), 1.64-1.52 (m, 4H), 1.46-1.39 (m, 2H), 1.08-0.98 (m, 2H), 0.92-0.81 (m, 4H), 0.52-0.58 (m, 6H), 0.28 (t, *J* = 7.4 Hz, 3H). ^{13}C NMR (125 MHz, 298 K, THF-*d*8): δ ppm 138.2, 137.9, 136.0, 135.8, 135.7, 135.5, 135.1, 134.1, 133.7, 133.3, 133.1, 133.1, 131.0, 130.4, 130.0, 129.9, 129.7, 129.3, 129.2, 129.1, 129.0, 128.9, 128.7, 128.4, 128.1, 127.5, 127.5, 127.4, 127.3, 126.9, 126.6, 126.3, 125.6, 122.7, 122.2, 33.1, 33.1, 32.7, 30.5, 29.6, 29.4, 29.0, 27.8, 25.6, 25.5, 25.4, 25.3, 25.2, 25.1, 25.0, 24.8, 22.7, 22.7, 22.2, 21.2, 20.1, 20.1, 14.0, 13.5. HRMS analysis (APCI, *m/z*) $[(M+H)^+]$ calcd for $C_{110}H_{103}$: 1423.8054, found 1423.8044 (error: 0.70 ppm).

2. Additional spectra

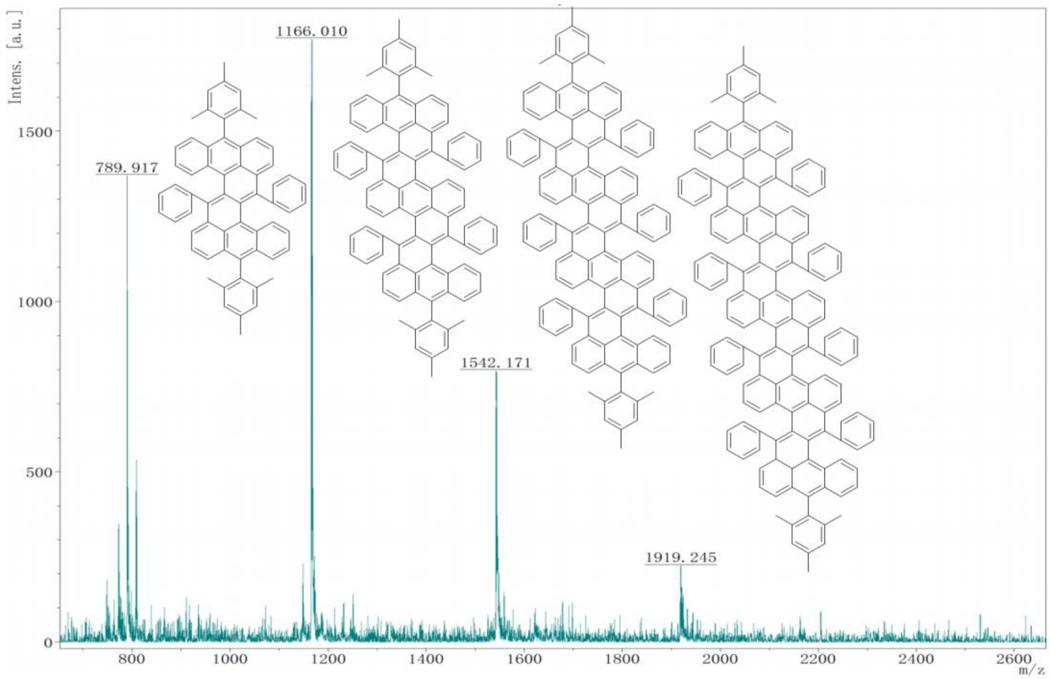

Figure S1. MALDI-TOF mass spectrum of the crude product after the cyclodimerization reaction between **5a** and **7a**. In addition to the phenyl-substituted 1,2:8,9-dibenzozethrene (DBZ) and the fused DBZ dimer and trimer, tetramer was also observed, but could not be isolated.

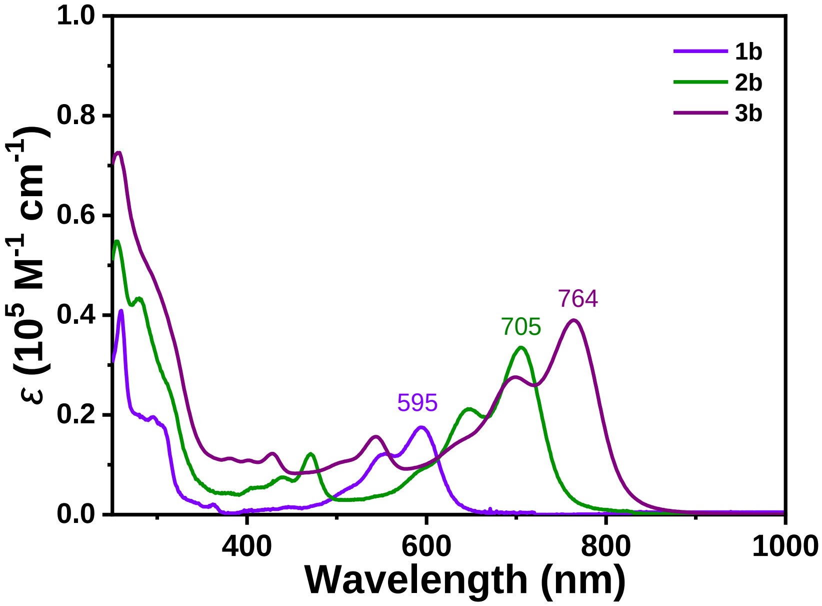

Figure S2. UV-vis-NIR absorption spectra of **1b**, **2b**, and **3b** in dichloromethane (1×10^{-5} M).

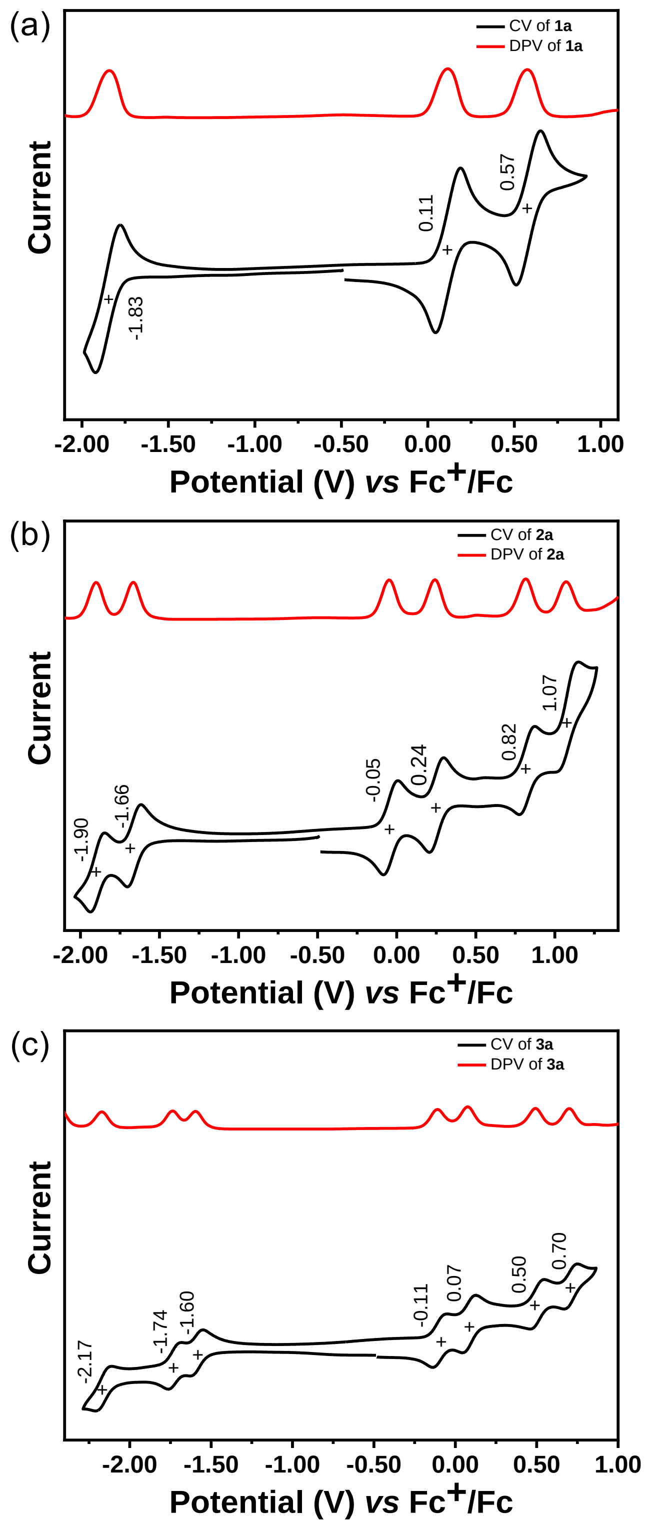

Figure S3. Cyclic voltammogram and differential pulse voltammogram and of (a) **1a**, (b) **2a**, and (c) **3a** (V vs. Fc/Fc$^+$, in 0.1 M *n*-Bu_4NPF_6/dichloromethane).

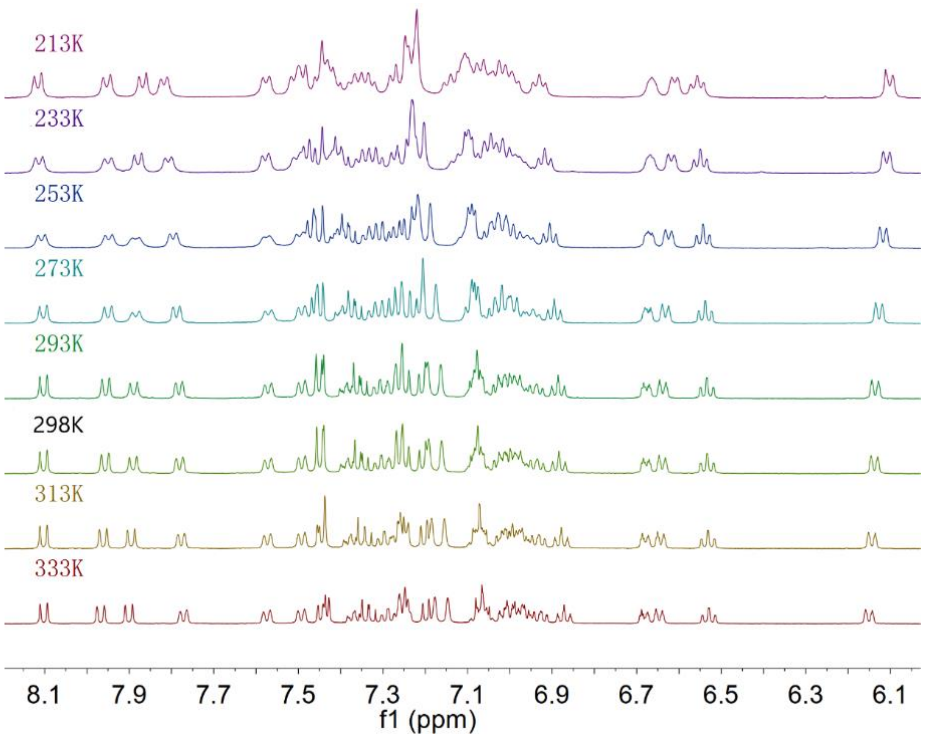

Figure S4. Variable-temperature ^{1}H NMR spectra of **3a** at the aromatic region (THF-d_8, 500 MHz). The broadening at low temperatures could be due to aggregation.

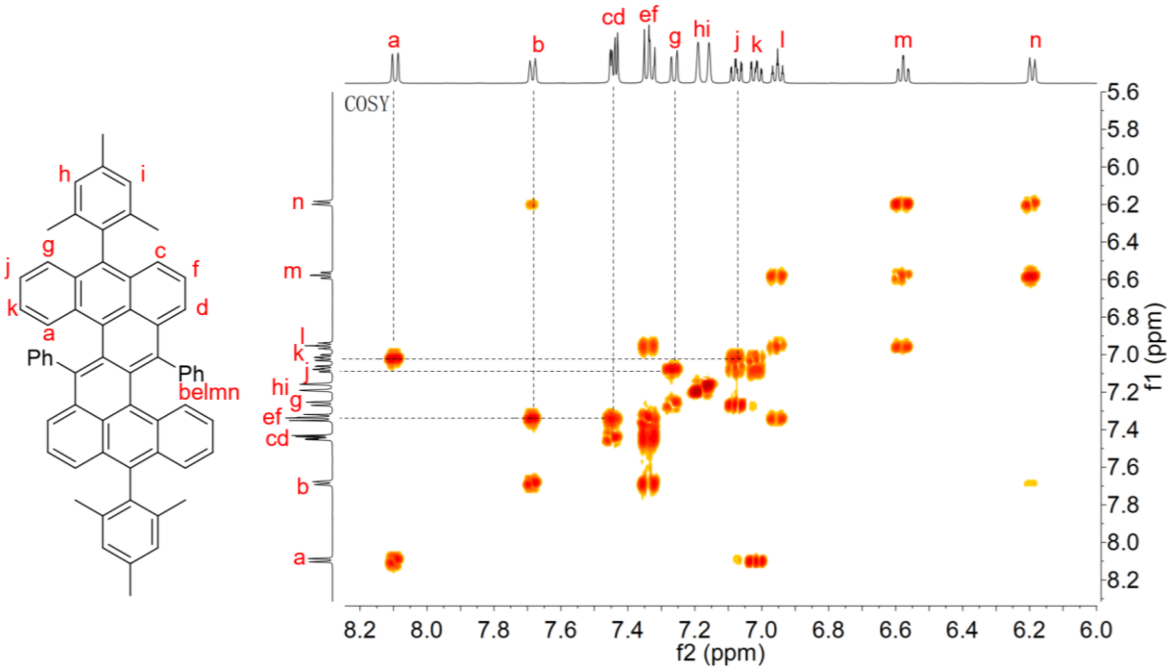

Figure S5. Partial 2D COSY NMR spectrum of **1a** in deacidified CD_2Cl_2 (500 MHz) at 298 K.

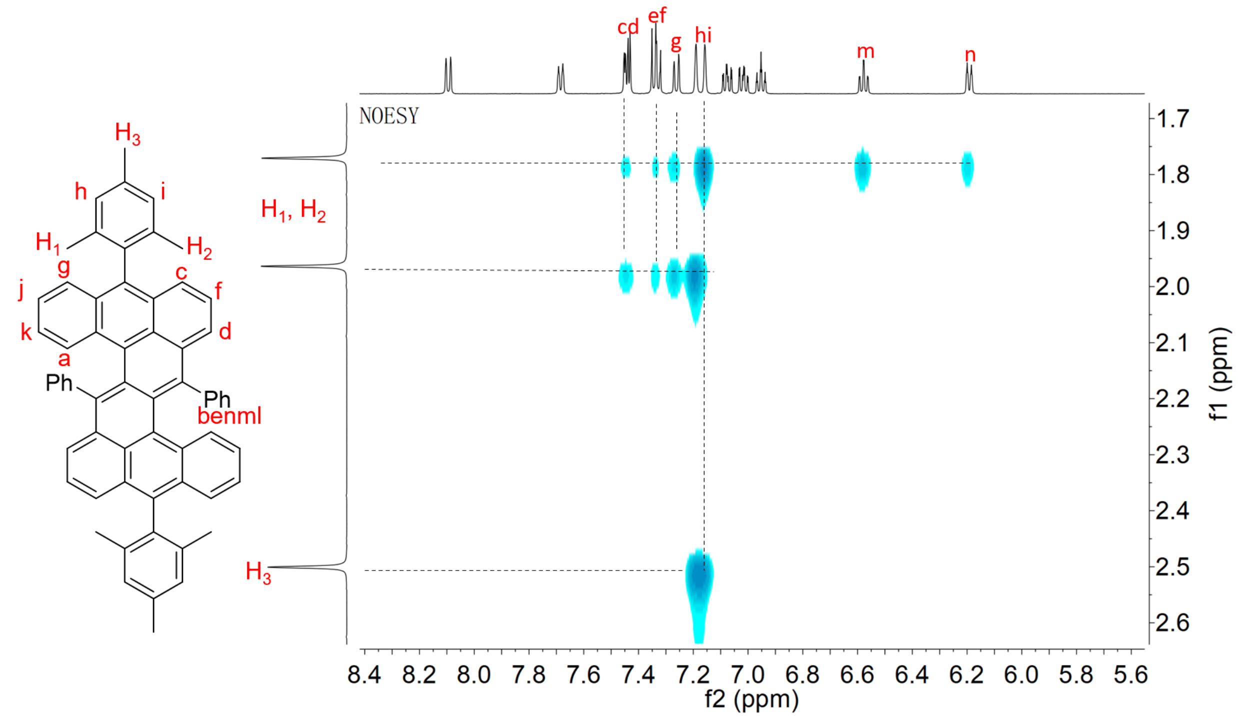

Figure S6. Partial 2D NOESY NMR spectrum of **1a** in deacidified CD_2Cl_2 (500 MHz) at 298 K.

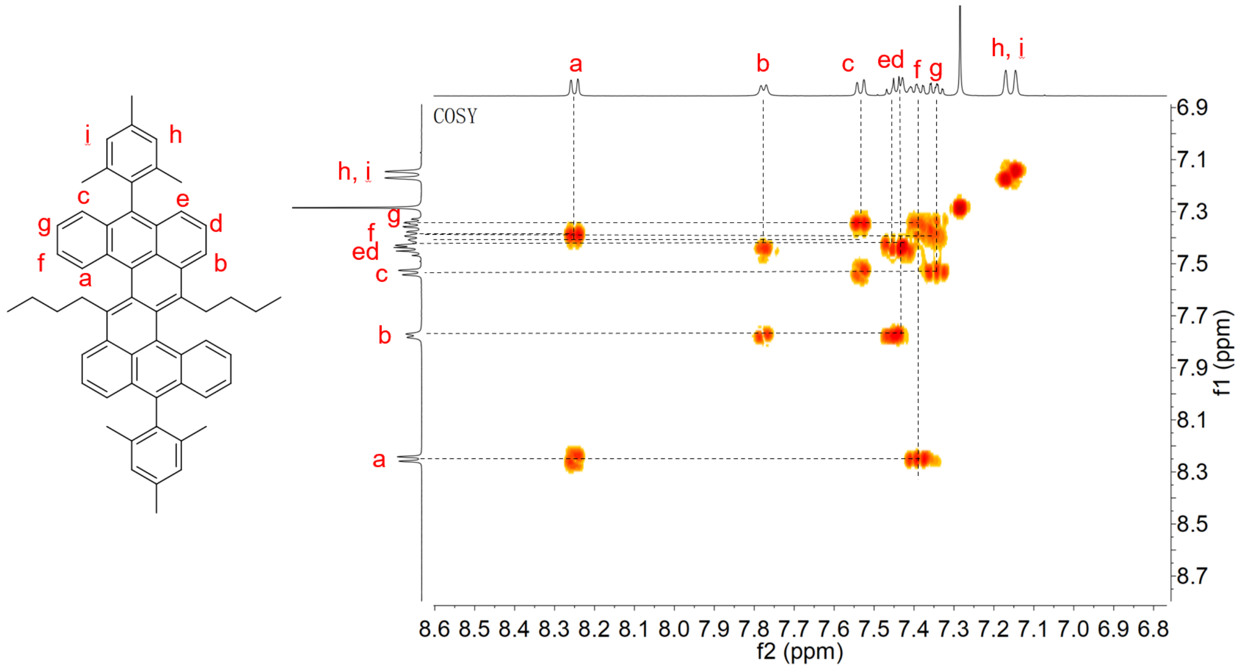

Figure S7. Partial 2D COSY NMR spectrum of **1b** in deacidified $CDCl_3$ (500 MHz) at 298 K.

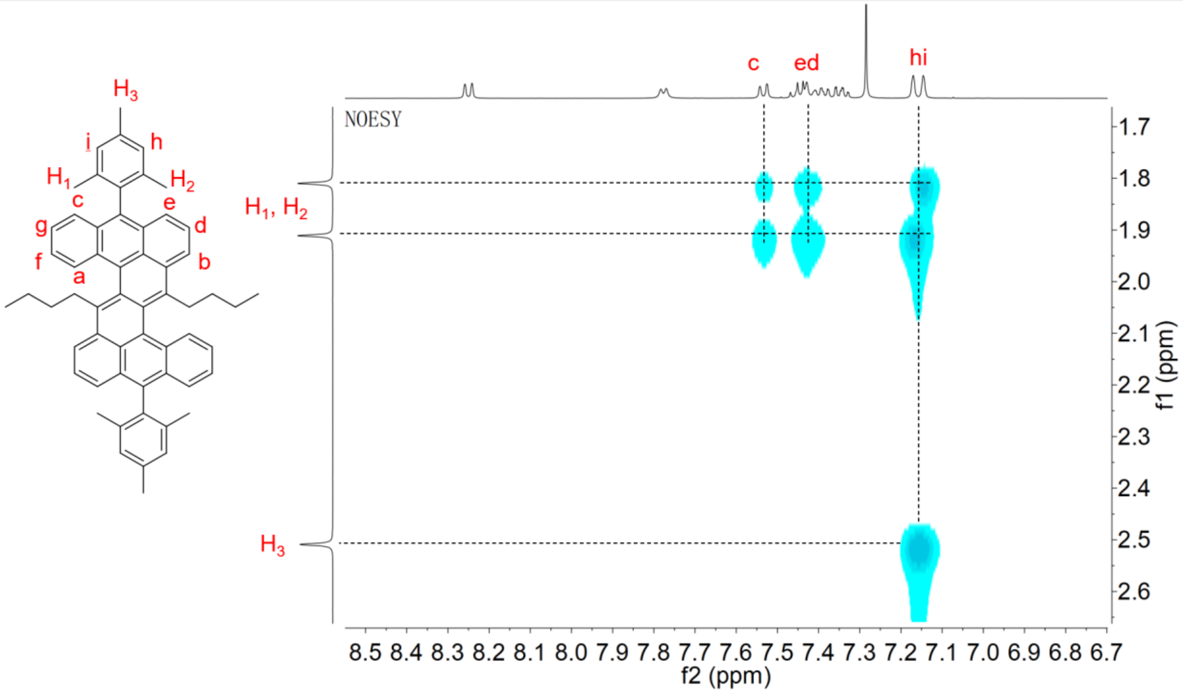

Figure S8. Partial 2D NOESY NMR spectrum of **1b** in deacidified $CDCl_3$ (500 MHz) at 298 K.

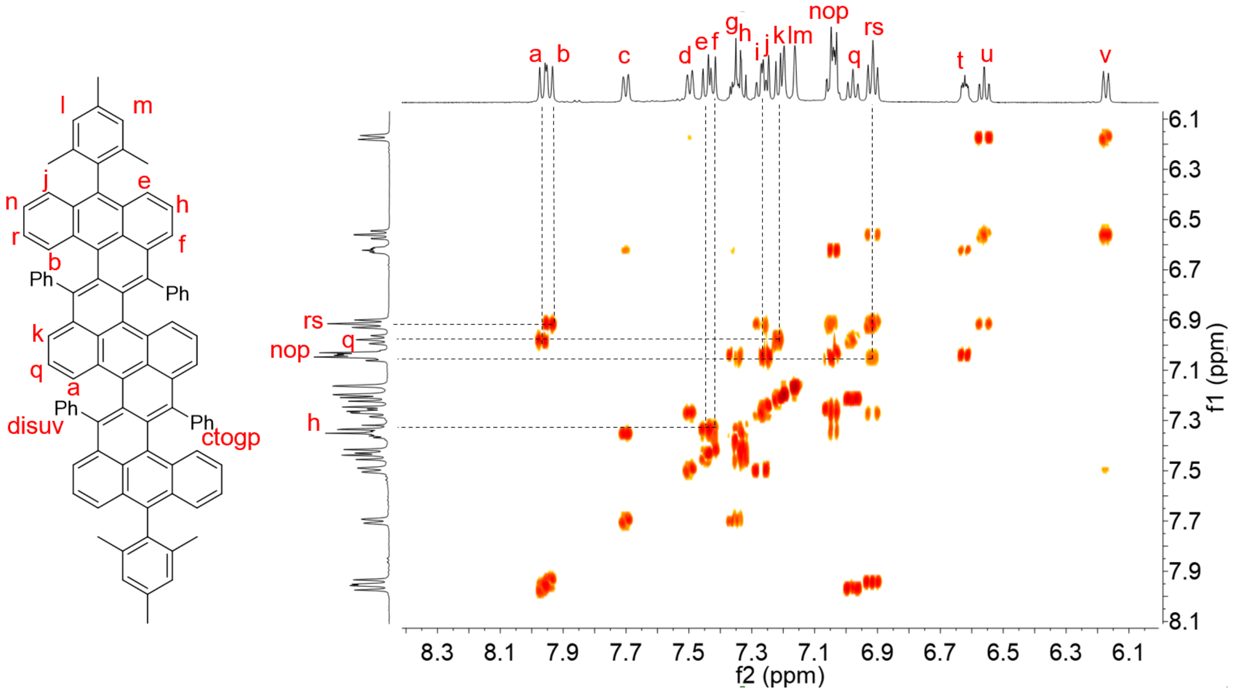

Figure S9. Partial 2D COSY NMR spectrum of **2a** in deacidified CD_2Cl_2 (500 MHz) at 298 K.

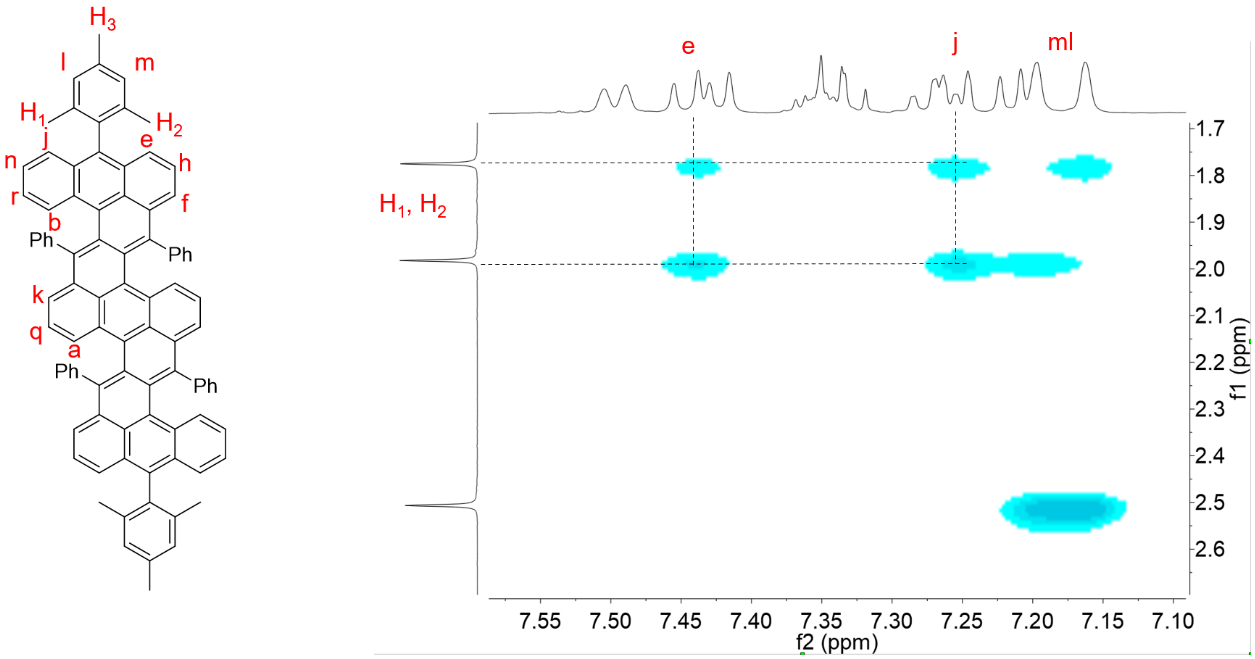

Figure S10. Partial 2D NOESY NMR spectrum of **2a** in deacidified CD_2Cl_2 (500 MHz) at 298 K.

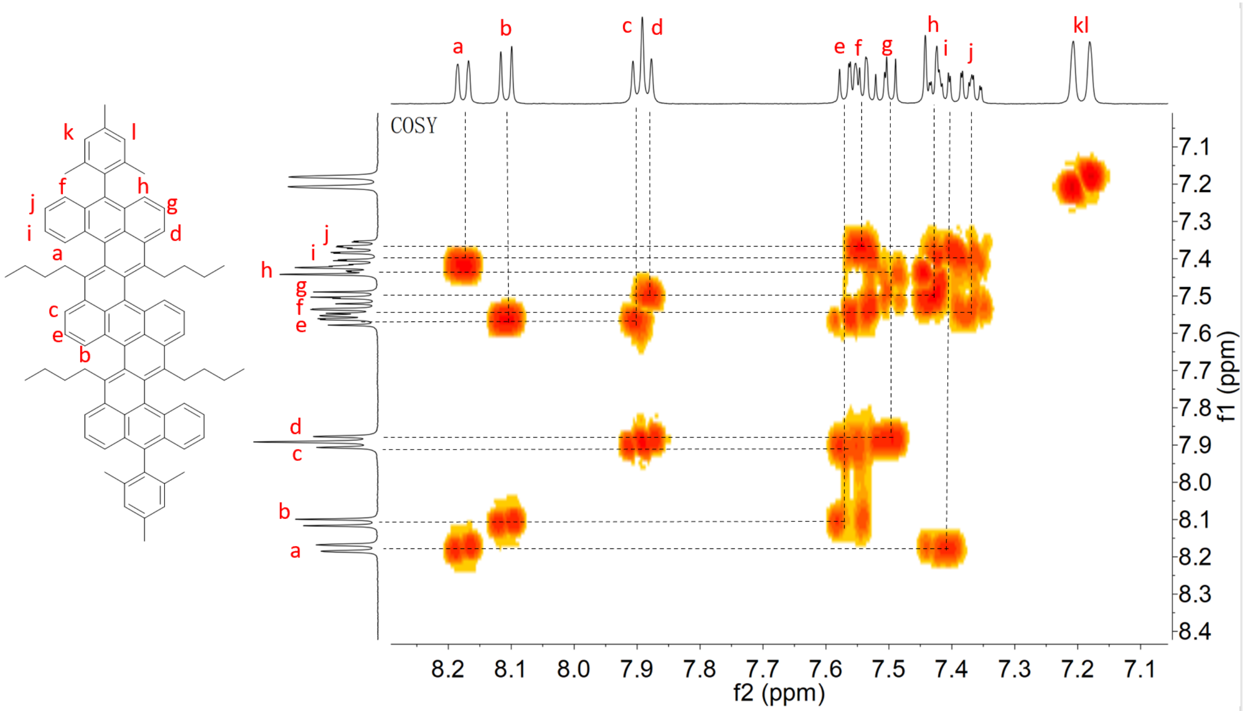

Figure S11. Partial 2D COSY NMR spectrum of **2b** in THF-d_8 (500 MHz) at 298 K.

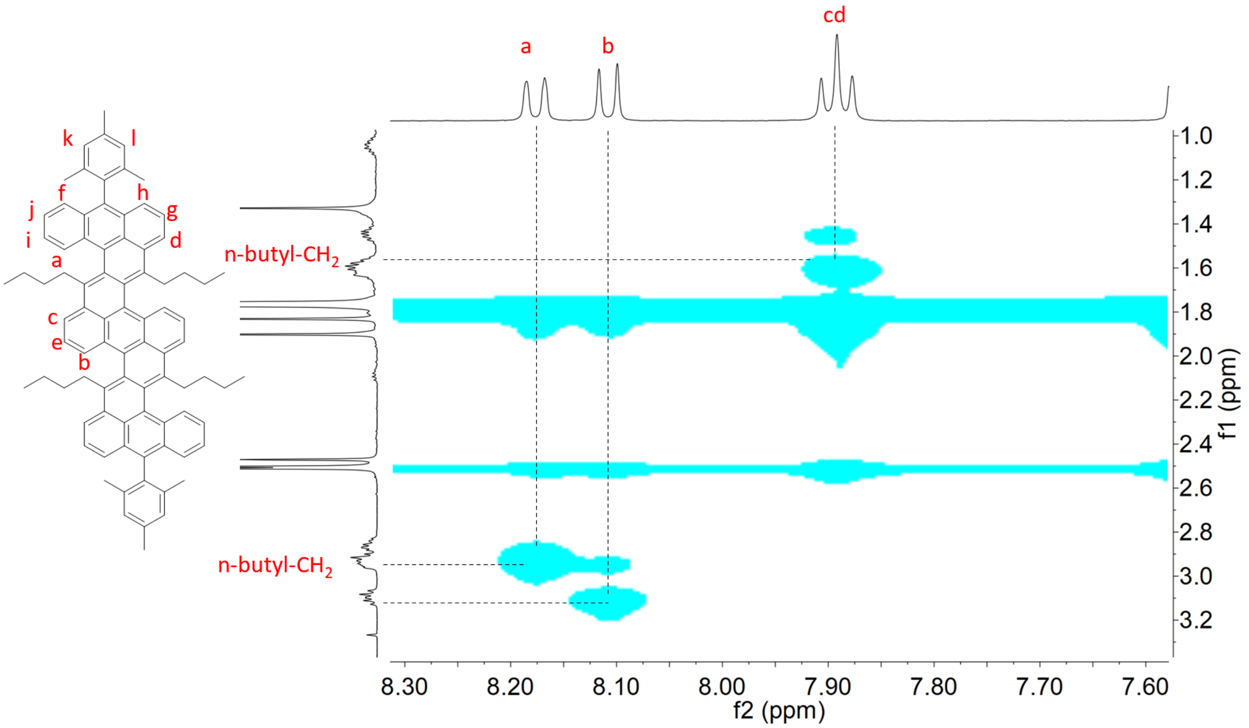

Figure S12. Partial 2D NOESY NMR spectrum of **2b** in THF-d_8 (500 MHz) at 298 K.

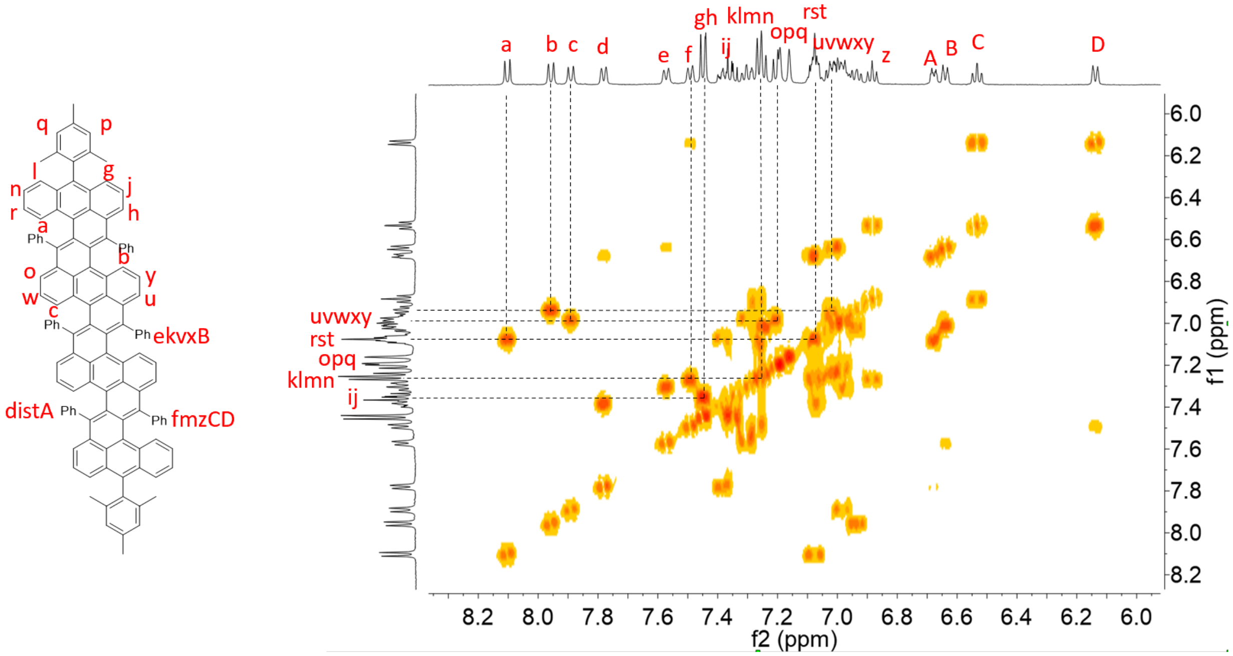

Figure S13. Partial 2D COSY NMR spectrum of **3a** in THF-d_8 (500 MHz) at 298 K.

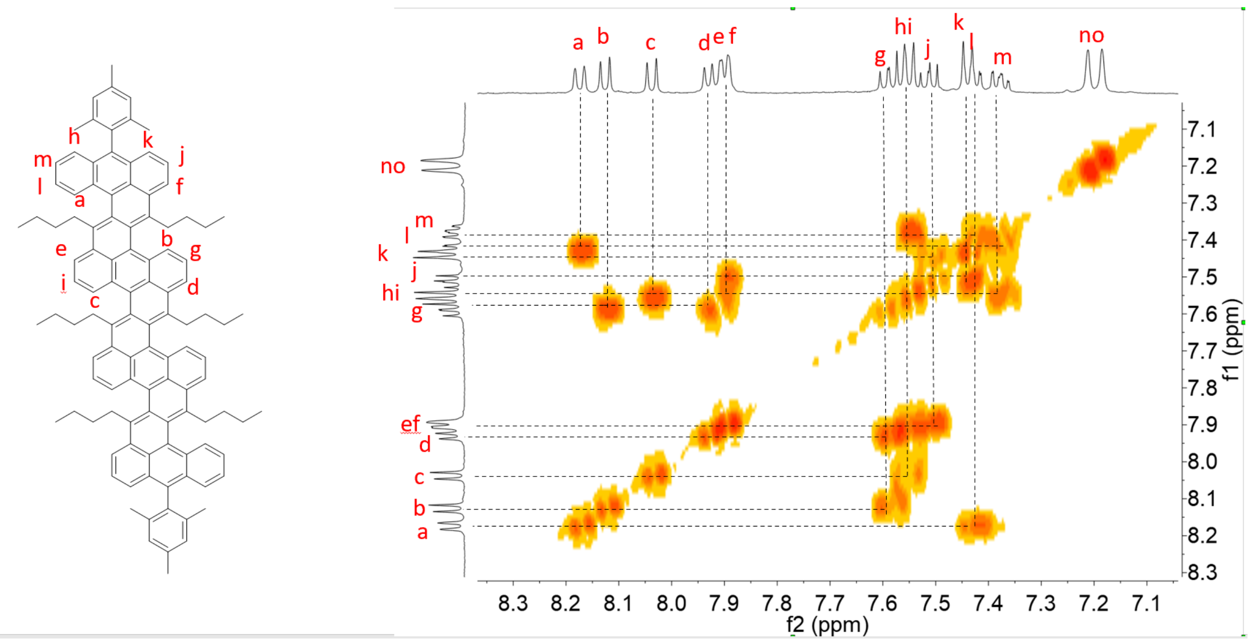

Figure S14. Partial 2D COSY NMR spectrum of **3b** in THF-d_8 (500 MHz) at 298 K.

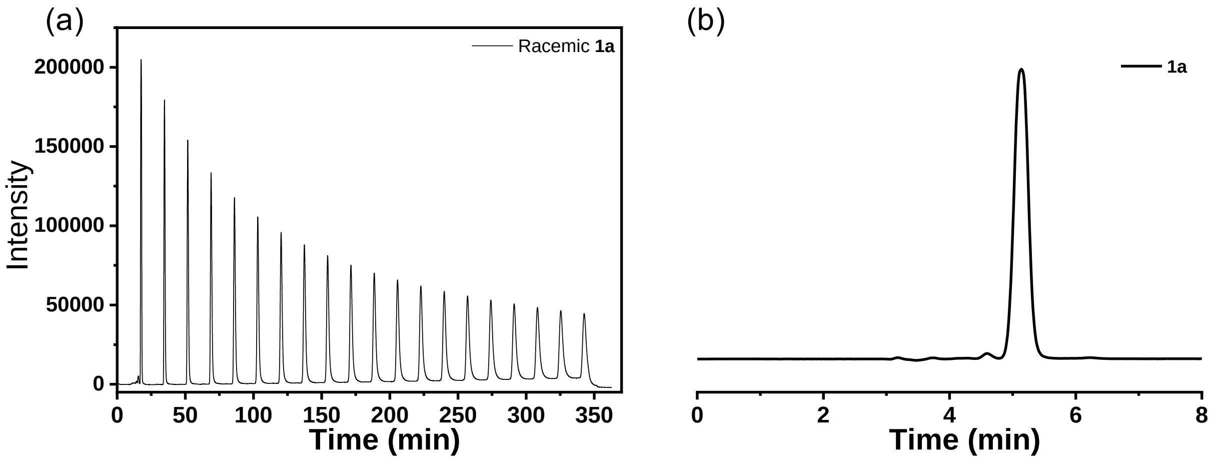

Figure S15. (a) Preparative chiral HPLC result for the isolation of racemic **1a**. [Conditions] Column: COSMOSIL Cholester 20ϕ × 250 mm, 25 °C, UV detector: 353 nm, eluent: acetone, flow rate: 6.0 mL min^{-1}. (b) Analytical chiral HPLC curve for **1a**. [Conditions] Column: COSMOSIL Cholester 4.6ϕ × 250 mm, 25 °C, UV detector: 353 nm, eluent: acetone, flow rate: 1.0 mL min^{-1}.

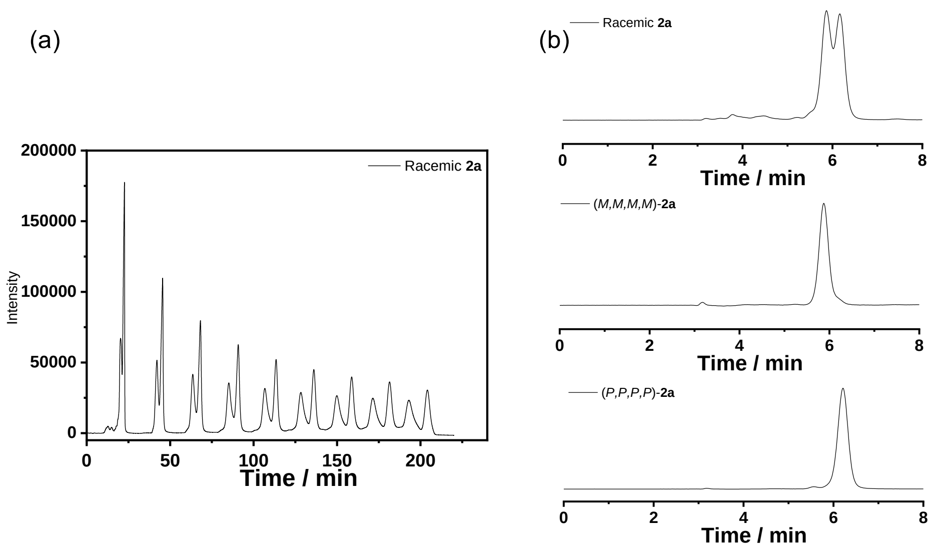

Figure S16. (a) Preparative chiral HPLC result for the isolation of racemic **2a**. [Conditions] Column: COSMOSIL Cholester 20ϕ × 250 mm, 25 °C, UV detector: 353 nm, eluent: acetone, flow rate: 6.0 mL min^{-1}. (b) Analytical chiral HPLC curves for **2a** and its two enantiomers. [Conditions] Column: COSMOSIL Cholester 4.6ϕ × 250 mm, 25 °C, UV detector: 353 nm, eluent: acetone, flow rate: 1.0 mL min^{-1}.

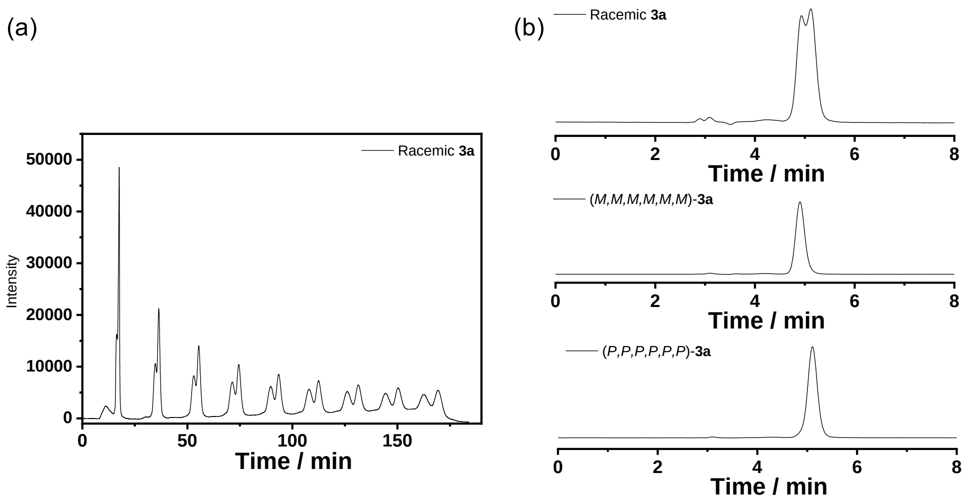

Figure S17. (a) Preparative chiral HPLC result for the isolation of racemic **3a**. [Conditions] Column: COSMOSIL Cholester 20ϕ × 250 mm, 25 °C, UV detector: 353 nm, eluent: acetone/THF (9:1 v/v), flow rate: 6.0 mL min^{-1}. (b) Analytical chiral HPLC curves for **3a** and its two enantiomers. [Conditions] Column: COSMOSIL Cholester 4.6ϕ × 250 mm, 25 °C, UV detector: 353 nm, eluent: acetone/THF (9:1 v/v), flow rate: 1.0 mL min^{-1}.

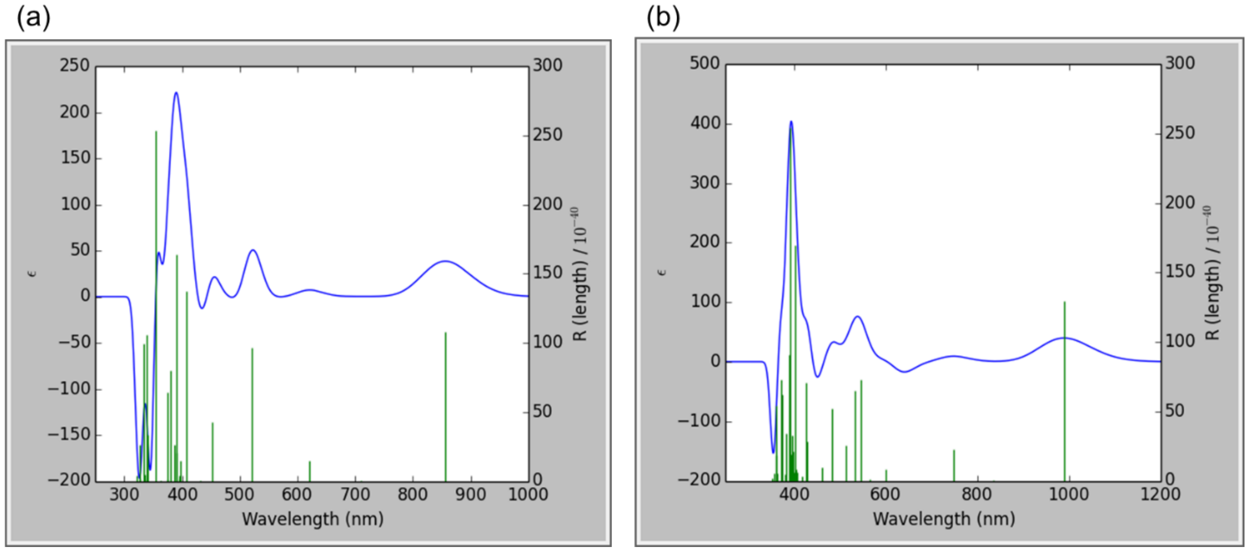

Figure S18. Simulated CD spectra of (a) (*P*,*P*,*P*,*P*)-**2a** and (b) (*P*,*P*,*P*,*P*,*P*,*P*)-**3a**.

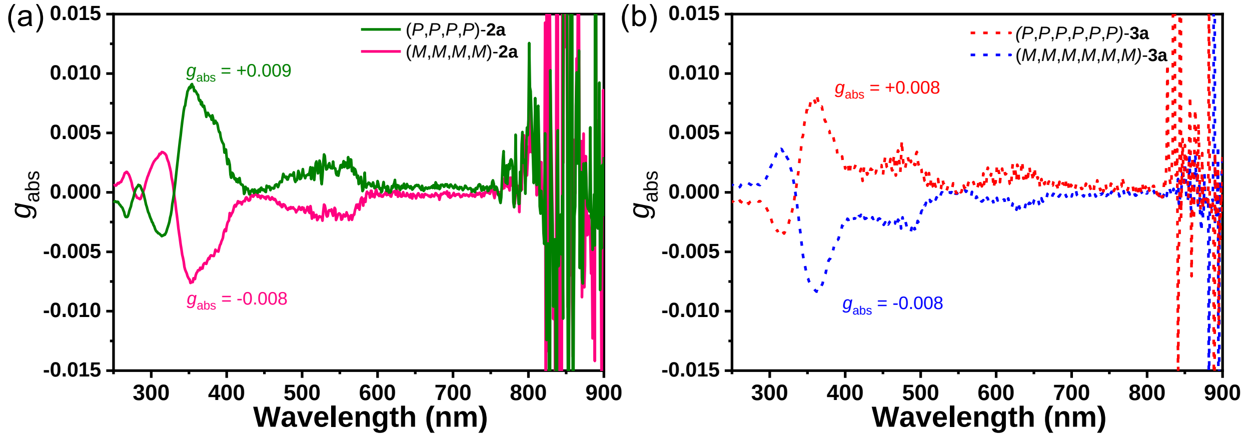

Figure S19. Dissymmetry factors (g_{abs}) of the enantiomers of **2a** and **3a**.

3. Thermal stability

Thermal racemization of (*M*,*M*,*M*,*M*)-2a at 100 ºC

Thermal racemization of **2a** was monitored by chiral HPLC. It switches between the (*M*)- and (*P*)-form isomers following a reversible first order reaction.[2] Rate constant (k) could be obtained

by fitting the experimental data (*α*, mole ratio of (*M*,*M*,*M*,*M*)-**2a** at time *t*) using the following equation:

$$ln(2\alpha - 1) = -2kt$$

Then the racemization barrier ($\Delta G^{\ddagger}$) was calculated from the Eyring equation:

$$\Delta G^{\ddagger}(T) = -RTln(kh/\kappa k_b T)$$

where *R* is the gas constant (*R* = 8.31441 J K^{-1} mol^{-1}); *h* is the Planck constant (*h* = 6.626176 × 10^{-34} J s); *kB* is the Boltzmann constant (*kB* = 1.380662 × 10^{-23} J K^{-1}); *κ* is the transmission coefficient (*κ* = 1).

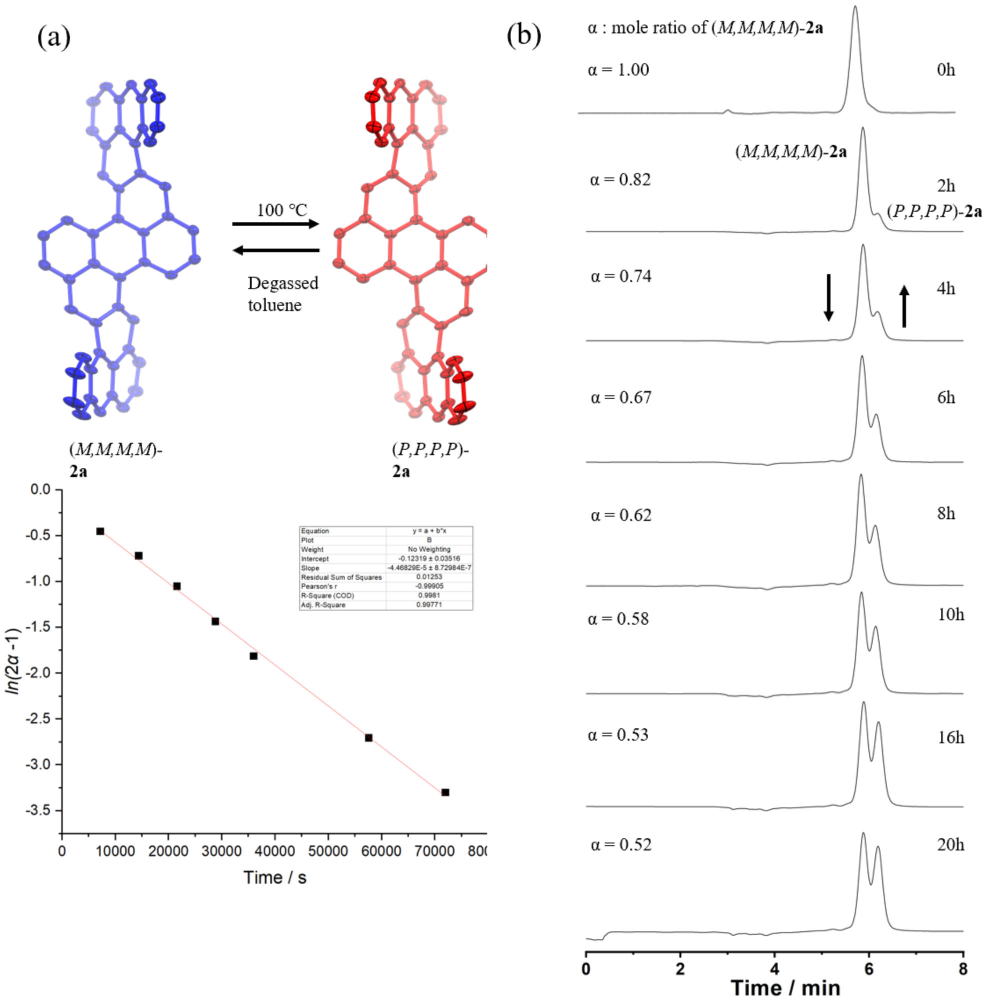

Figure S20. (a) Thermal racemization of (*M*,*M*,*M*,*M*)-**2a** and fitted plot. (b) HPLC analysis of (*M*,*M*,*M*,*M*)-**2a** after heating in degassed toluene at 100 °C. [Conditions] Column: COSMOSIL Cholester 4.6ϕ × 250 mm, 25 °C, UV detector: 720nm, eluent: acetone, flow rate: 1.0 mL min^{-1}.

4. DFT calculations

Density functional theory (DFT) calculations were performed with the Gaussian09 program suite[3] with Becke's three-parameter hybrid exchange functionals and the Lee-Yang-Parr correlation functional (B3LYP) employing the 6-31G(d,p) basis set for all atoms.[4] Time-dependent DFT (TD-DFT) calculations were performed at the B3LYP/6-31G(d,p) level of theory. NICS values were calculated (B3LYP/6-31G(d,p)) using the standard GIAO procedure (NMR=GIAO).[5] ACID plot (B3LYP/6- 31G(d,p)) was calculated by using the method developed by Herges based on the optimized ground-state geometries.[6]

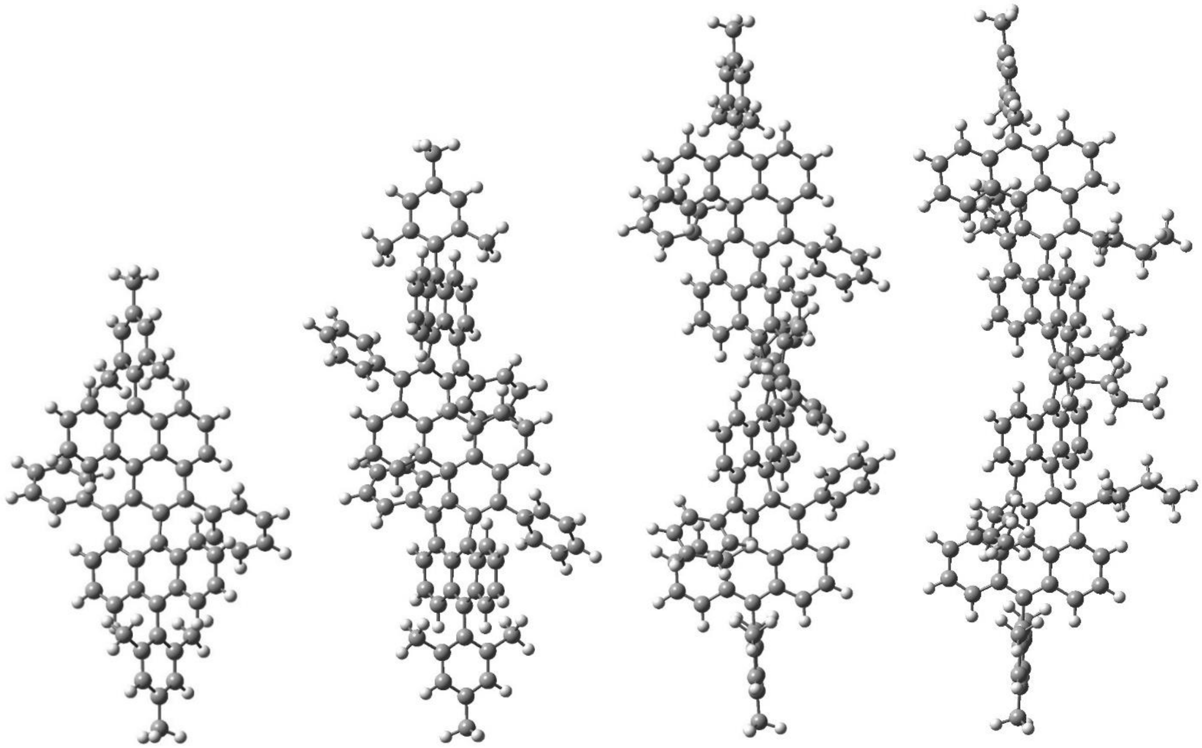

Figure S21. Optimized geometries of (*P*,*P*)-**1a**, (*P*,*P*,*P*,*P*)-**2a**, (*P*,*P*,*P*,*P*,*P*,*P*)-**3a**, and (*P*,*P*,*P*,*P*,*P*,*P*)-**3b** at B3LYP/6-31G(d,p) level of theory showing a twisted backbone.

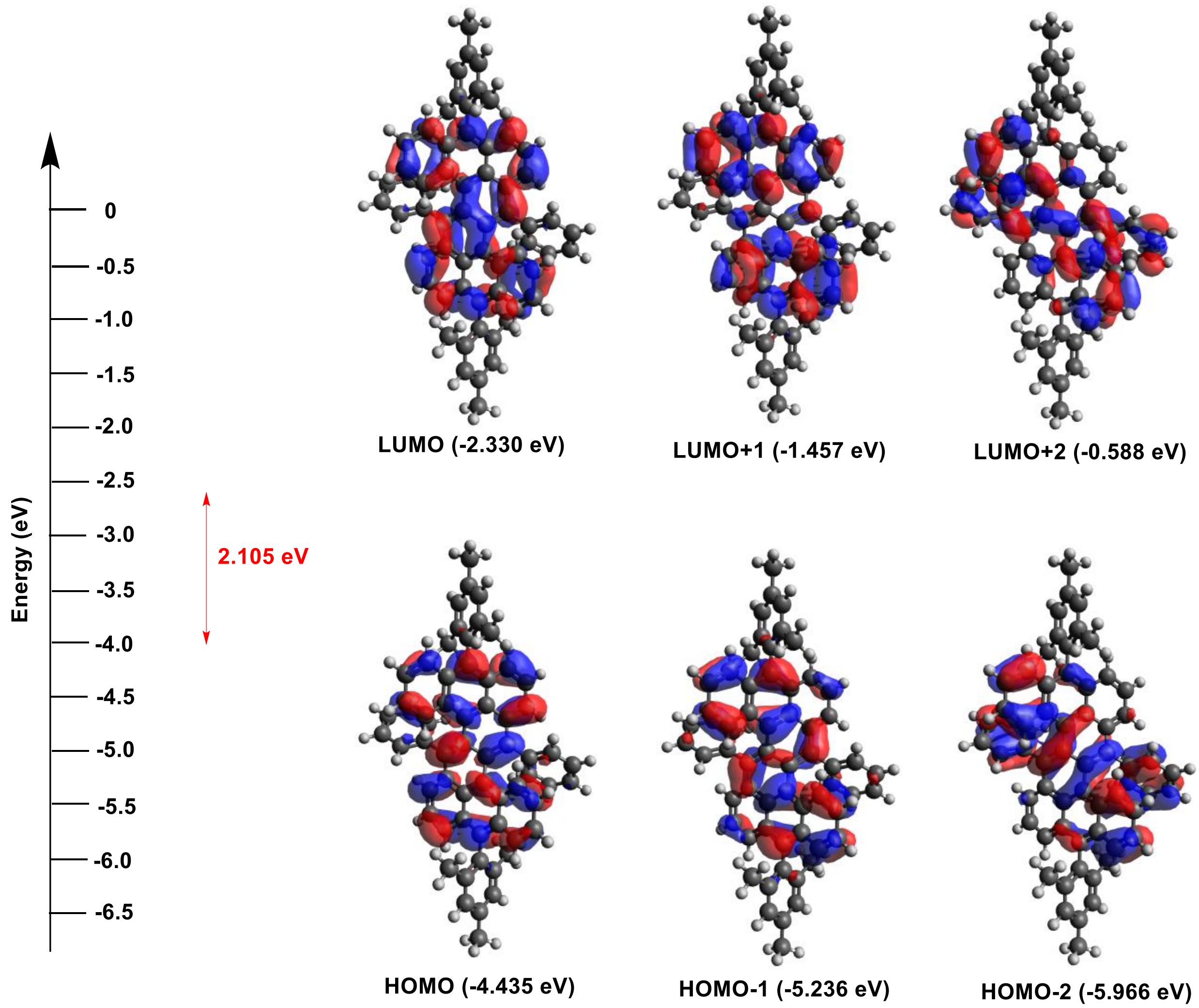

Figure S22. Calculated (B3LYP/6-31G(d,p)) frontier molecular orbital profiles and energy diagram of **1a**.

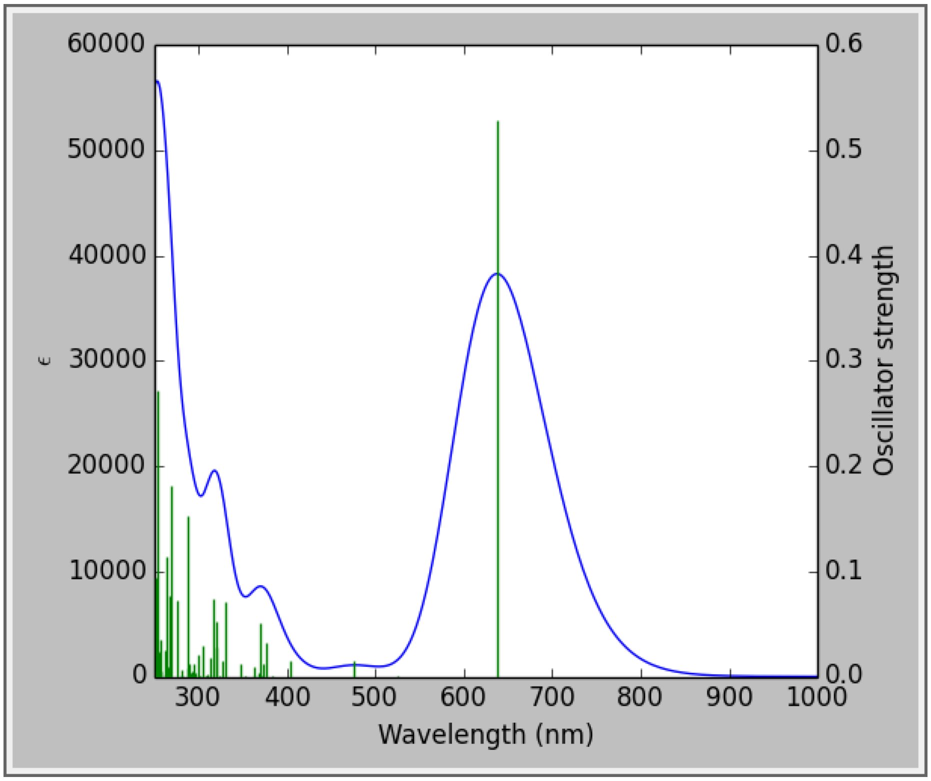

Figure S23. Calculated (B3LYP/6-31G (d,p)) absorption spectrum of **1a**.

Table S1. Selected TD-DFT (B3LYP/6-31G(d,p)) calculated wavelength, oscillator strength and compositions of major electronic transitions of **1a**.

Energy (cm^{-1})	Wavelength (nm)	Osc. Strength	Major contributions
15680.22	637.746	0.5279	HOMO->LUMO (100%)
20986.55	476.4957	0.0146	H-1->LUMO (36%), HOMO->L+1 (60%)
24728.96	404.3842	0.0148	H-2->LUMO (88%), HOMO->L+2 (10%)
26551.77	376.6227	0.0324	H-1->L+1 (24%), HOMO->L+2 (66%)
26834.87	372.6494	0.0125	H-3->LUMO (65%), H-1->L+1 (19%), H-9->LUMO (3%), H-4->L+1 (2%), HOMO->L+2 (7%)
26973.6	370.7329	0.0514	H-3->LUMO (27%), H-1->L+1 (52%), HOMO->L+2 (14%)
28755.28	347.7622	0.0126	H-10->LUMO (41%), H-8->LUMO (10%), HOMO->L+4 (40%)

30300.64	330.0261	0.0706	H-12->LUMO (35%), H-9->LUMO (22%), HOMO->L+5 (31%)
30571.64	327.1006	0.015	HOMO->L+6 (89%)
31195.91	320.5548	0.052	H-12->LUMO (32%), HOMO->L+7 (58%)
31274.15	319.7529	0.0281	H-10->LUMO (40%), HOMO->L+4 (25%)
31497.56	317.4849	0.0745	H-13->LUMO (46%), H-12->LUMO (15%), HOMO->L+5 (11%), HOMO->L+7 (18%)
34738.3	287.8667	0.1523	H-7->L+1 (14%), H-1->L+4 (36%), HOMO->L+13 (32%)
36180.42	276.3926	0.0725	H-14->LUMO (10%), H-10->L+1 (37%), H-8->L+1 (18%), H-1->L+6 (12%)
37206.36	268.7713	0.1815	H-10->L+1 (41%), H-1->L+6 (38%)
37891.93	263.9085	0.1136	H-11->L+1 (16%), H-8->L+1 (10%), H-1->L+6 (36%)

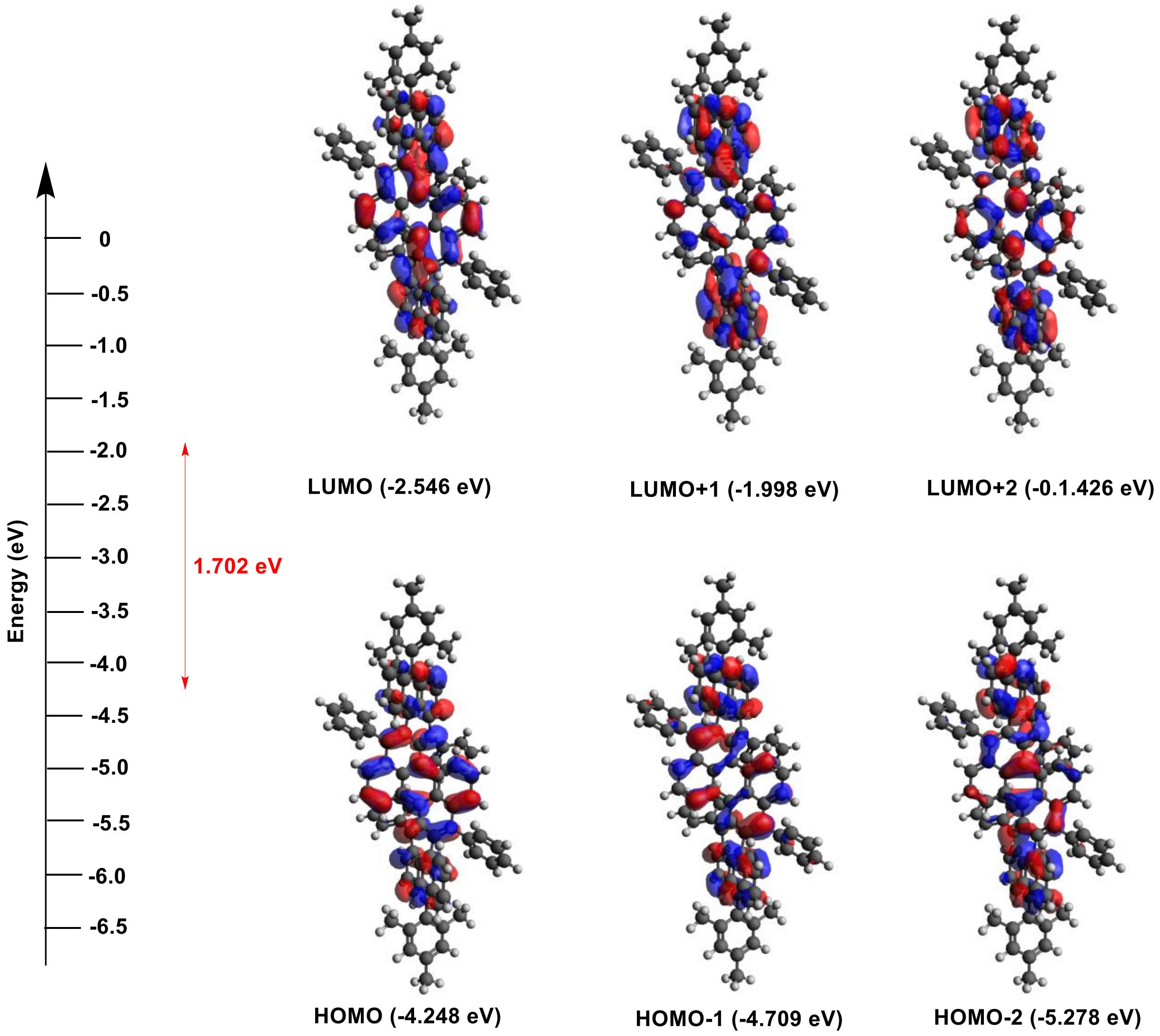

Figure S24. Calculated (B3LYP/6-31G(d,p)) frontier molecular orbital profiles and energy diagram of **2a**.

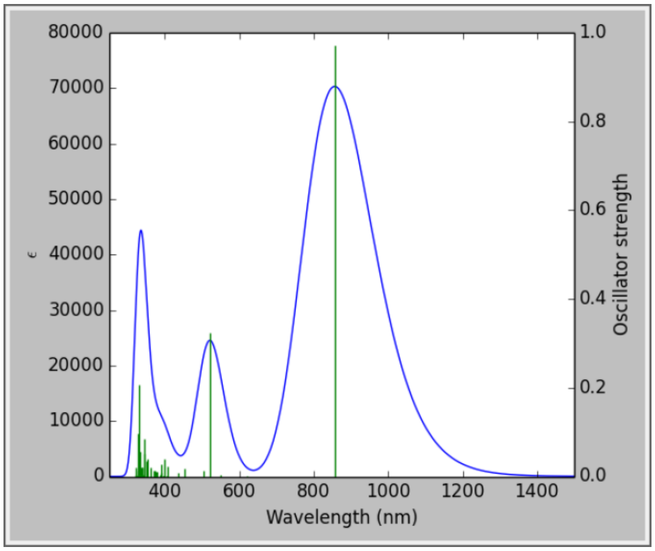

Figure S25. Calculated (B3LYP/6-31G (d,p)) absorption spectrum of **2a**.

Table S2. Selected TD-DFT (B3LYP/6-31G(d,p)) calculated wavelength, oscillator strength and compositions of major electronic transitions of **2a**.

**Energy (cm^{-1})**	**Wavelength (nm)**	**Osc. Strength**	**Major contributions**
11680.52	856.12	0.9704	HOMO->LUMO (100%)
19175.83	521.48	0.3227	H-1->L+1 (89%), H-2->LUMO (4%), HOMO->L+2 (5%)
19821.88	504.493	0.0127	H-2->LUMO (27%), HOMO->L+2 (62%)
22124.59	451.9857	0.0184	H-3->LUMO (76%), H-2->L+1 (11%)
24512	407.9635	0.0232	HOMO->L+3 (89%)
25586.33	390.8338	0.0269	H-9->LUMO (33%), HOMO->L+4 (39%)
26338.03	379.679	0.0108	H-12->LUMO (29%), H-10->LUMO (44%)
26650.17	375.2321	0.0111	H-11->LUMO (13%), H-8->LUMO (17%), H-1->L+3 (11%), HOMO->L+5 (37%)
26726.79	374.1564	0.0138	H-13->LUMO (30%), H-8->LUMO (18%), H-3->L+1 (11%)
26943.76	371.1435	0.013	H-13->LUMO (10%), H-6->LUMO (10%), H-3->L+1 (56%)
28926.27	345.7065	0.0848	H-18->LUMO (21%), H-1->L+3 (21%), HOMO->L+7 (11%), HOMO->L+9 (14%)

30216.76	330.9422	0.206	H-18->LUMO (29%), H-1->L+6 (11%), HOMO->L+9 (16%), HOMO->L+11 (11%)
30395	329.0014	0.0957	H-19->LUMO (26%), H-18->LUMO (12%), H-1->L+6 (20%), HOMO->L+11 (12%)

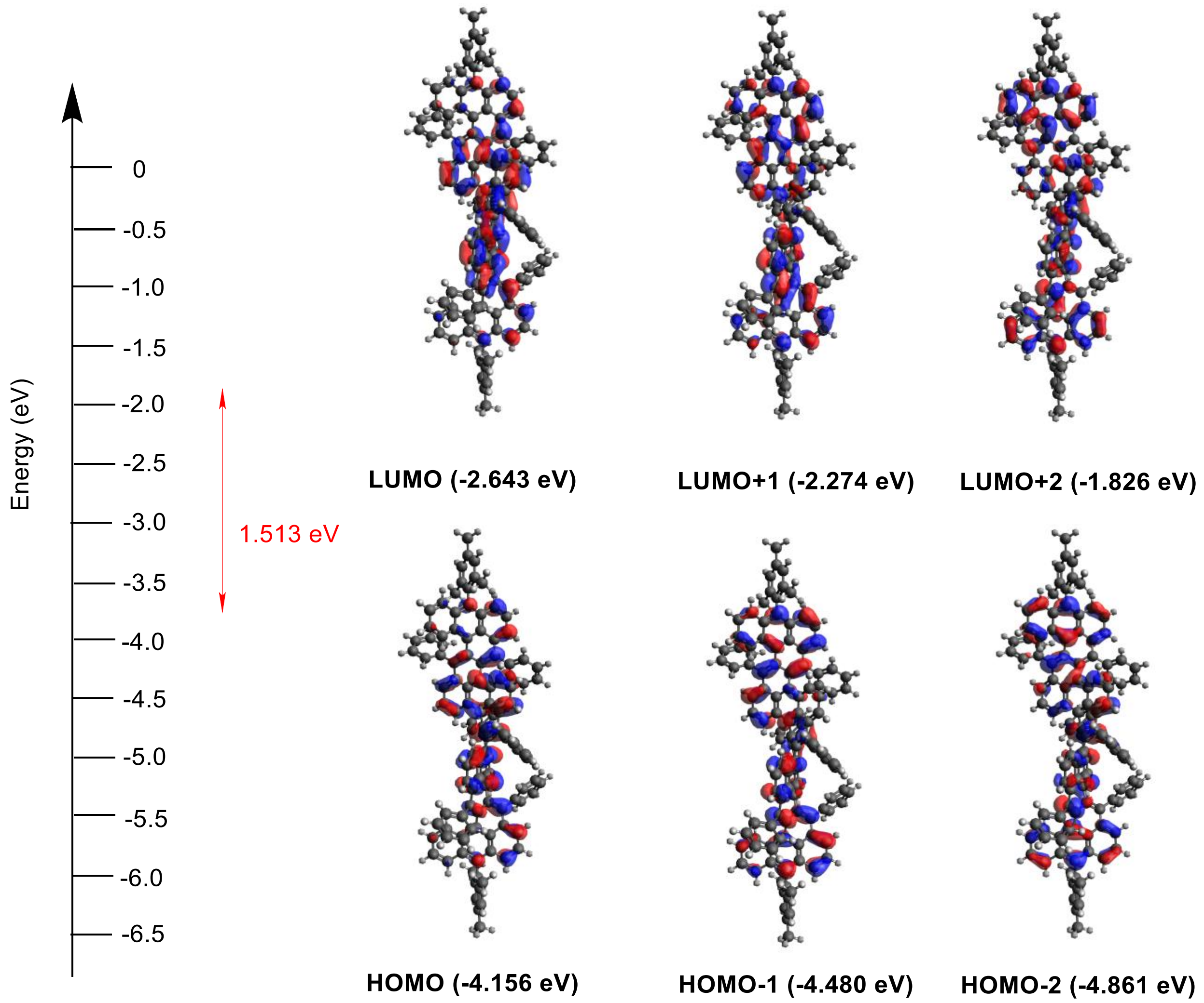

Figure S26. Calculated (B3LYP/6-31G(d,p)) frontier molecular orbital profiles and energy diagram of **3a**.

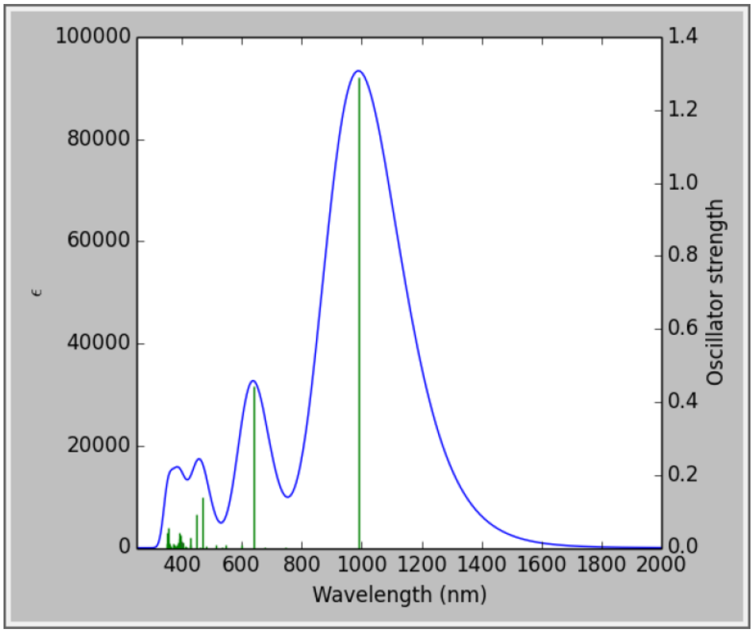

Figure S27. Calculated (B3LYP/6-31G (d,p)) absorption spectrum of **3a**.

Table S3. Selected TD-DFT (B3LYP/6-31G(d,p)) calculated wavelength, oscillator strength and compositions of major electronic transitions of **3a**.

Energy (cm^{-1})	Wavelength (nm)	Osc. Strength	Major contributions
10110.97	989.0252	1.2887	HOMO->LUMO (100%)
15660.06	638.5671	0.4428	H-1->L+1 (99%)
21271.26	470.1179	0.1404	H-3->L+1 (12%), H-2->L+2 (44%), H-1->L+3 (40%)
22348.01	447.4671	0.0912	H-3->L+1 (16%), H-2->L+2 (52%), H-1->L+3 (27%)
23390.08	427.5317	0.0299	H-5->LUMO (52%), H-4->L+1 (32%)
24845.1	402.4938	0.0166	H-8->LUMO (12%), H-5->LUMO (12%), HOMO->L+5 (40%)
25262.09	395.85	0.0362	H-14->LUMO (47%), H-11->LUMO (15%)
25445.99	392.9893	0.0262	H-12->LUMO (44%), H-11->LUMO (19%)
25624.23	390.2556	0.0414	H-11->LUMO (16%), HOMO->L+7 (54%)
26971.18	370.7661	0.0129	H-18->LUMO (51%), HOMO->L+13 (10%)

27951.95	357.7568	0.0116	H-8->L+1 (16%), H-5->L+1 (10%), HOMO->L+12 (25%)
28071.32	356.2355	0.0138	H-7->L+1 (12%), H-6->L+1 (28%)
28154.4	355.1843	0.0537	H-7->L+1 (10%), H-6->L+1 (20%), H-1->L+5 (10%)
28343.94	352.8092	0.0423	H-8->L+1 (39%), H-1->L+5 (19%)

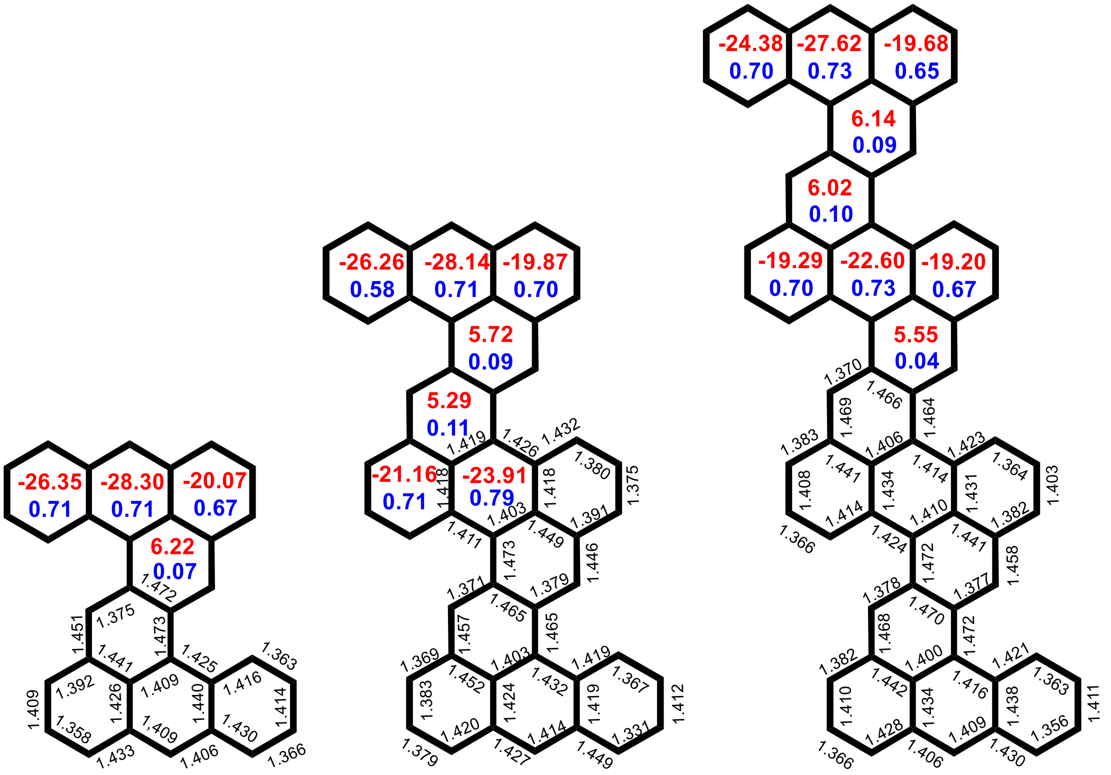

Figure S28. Calculated HOMA values (Blue), NICS(1)$_{ZZ}$ values (Red), and selected bond lengths based on the single-crystal structures of **1a**, **2a**, and **3b**.

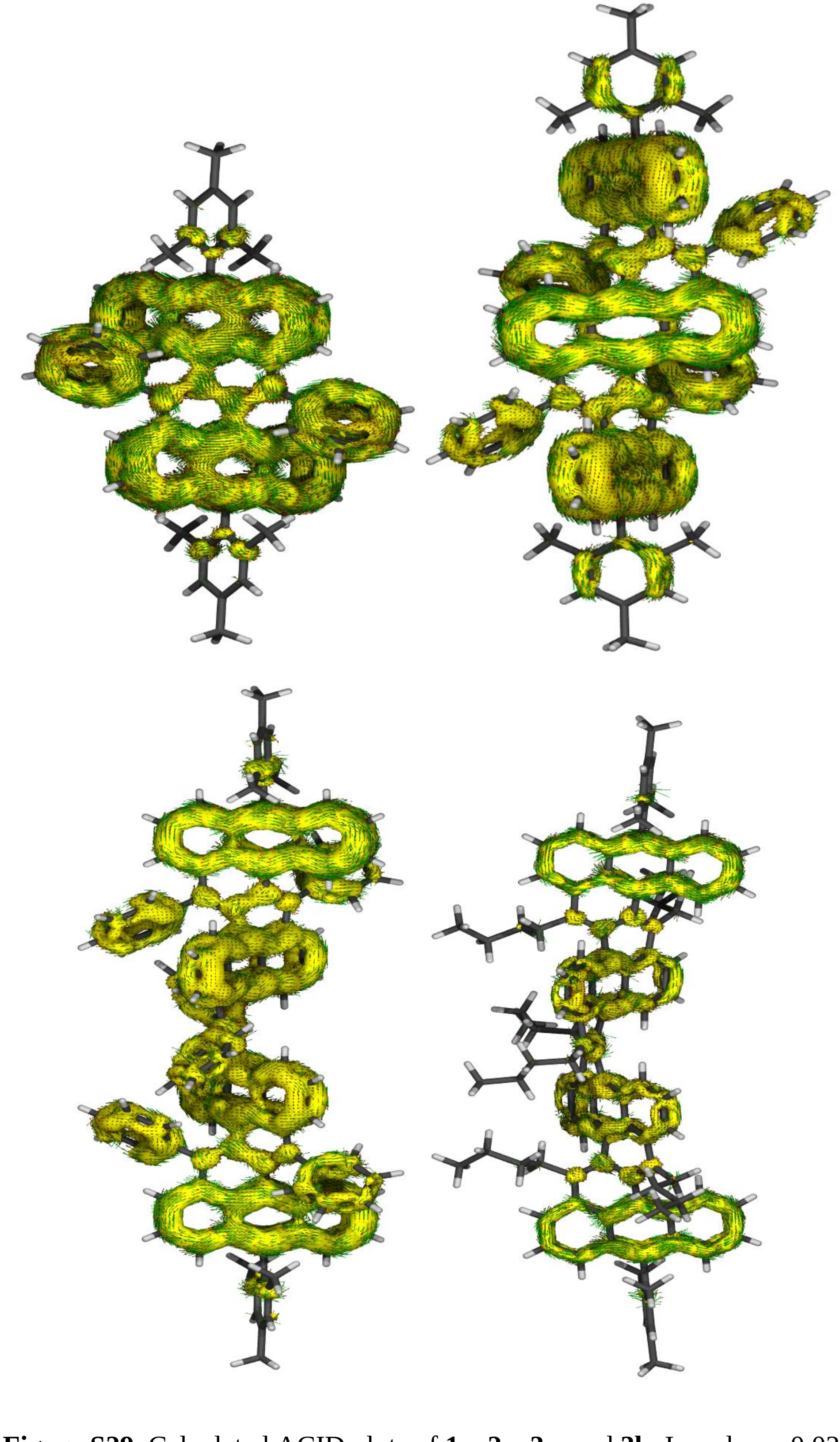

Figure S29. Calculated ACID plots of **1a**, **2a**, **3a**, and **3b.** Isovalue = 0.02.

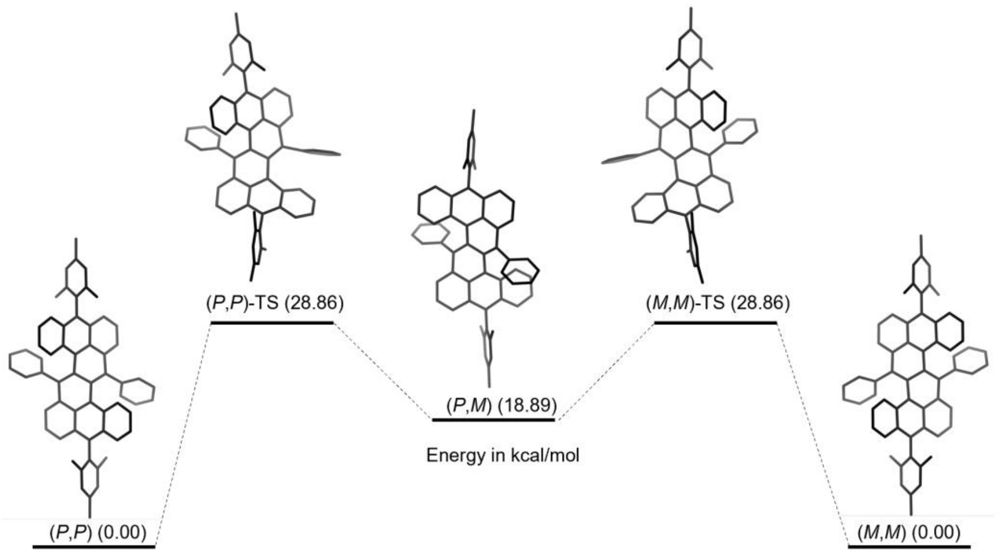

Figure S30. Computational studies on the racemic processes of **1a** at the B3LYP/6-31g (d,p) level of theory.

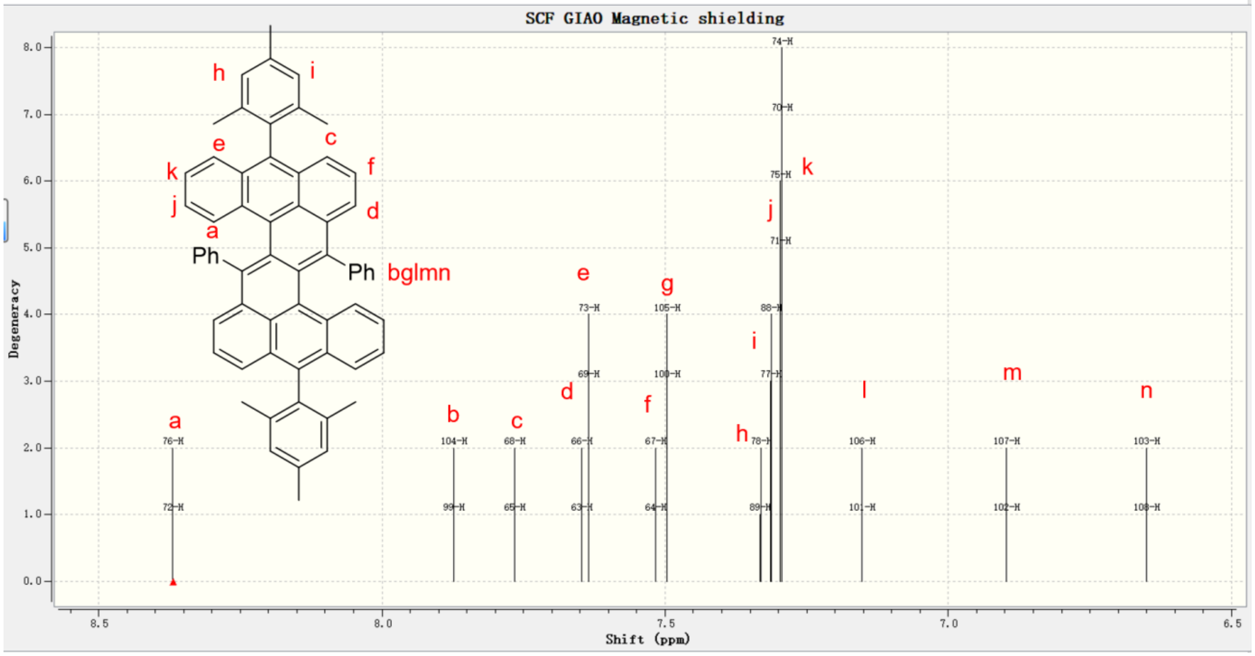

Figure S31. Calculated (B3LYP/6-31G(d,p)-GIAO) ^{1}H NMR spectrum of **1a**.

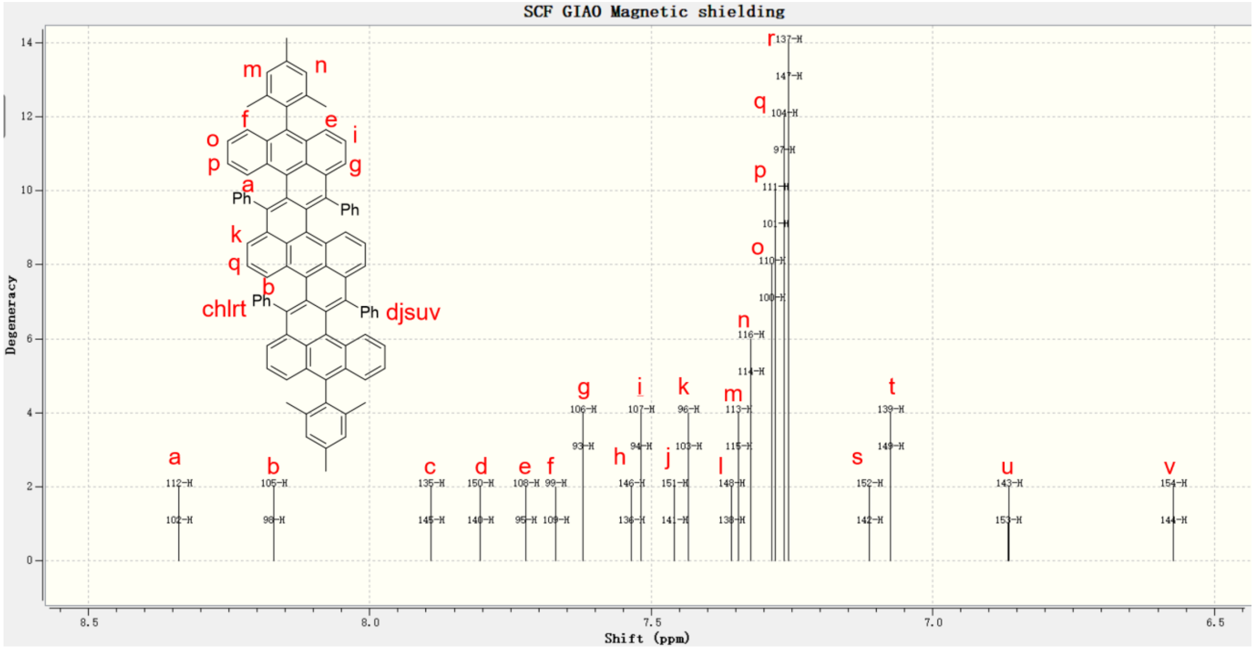

Figure S32. Calculated (B3LYP/6-31G(d,p)-GIAO) ^{1}H NMR spectrum of **2a**.

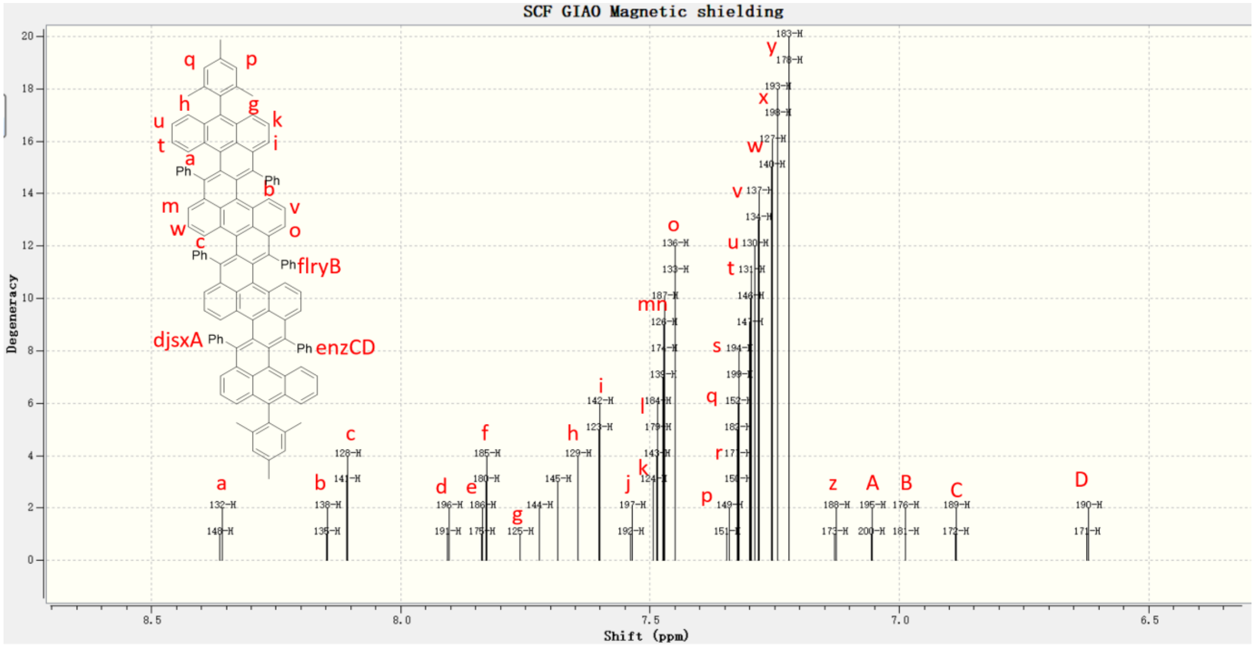

Figure S33. Calculated (B3LYP/6-31G(d,p)-GIAO) ^{1}H NMR spectrum of **3a**.

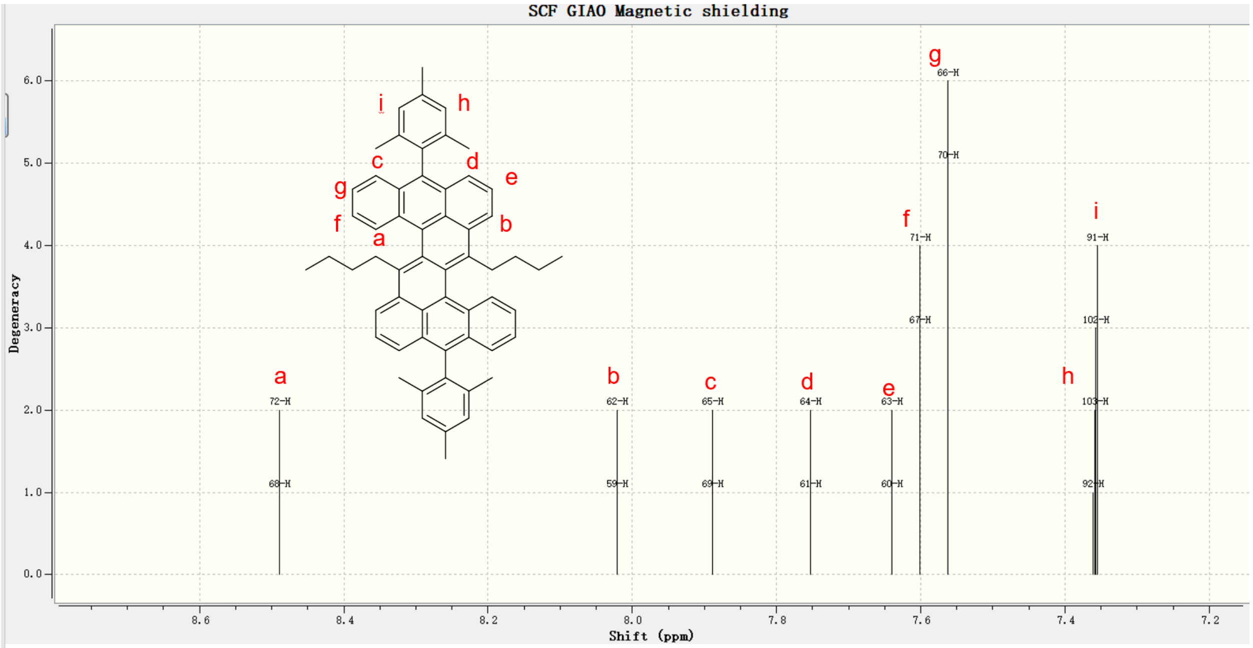

Figure S34. Calculated (B3LYP/6-31G(d,p)-GIAO) ^{1}H NMR spectrum of **1b**.

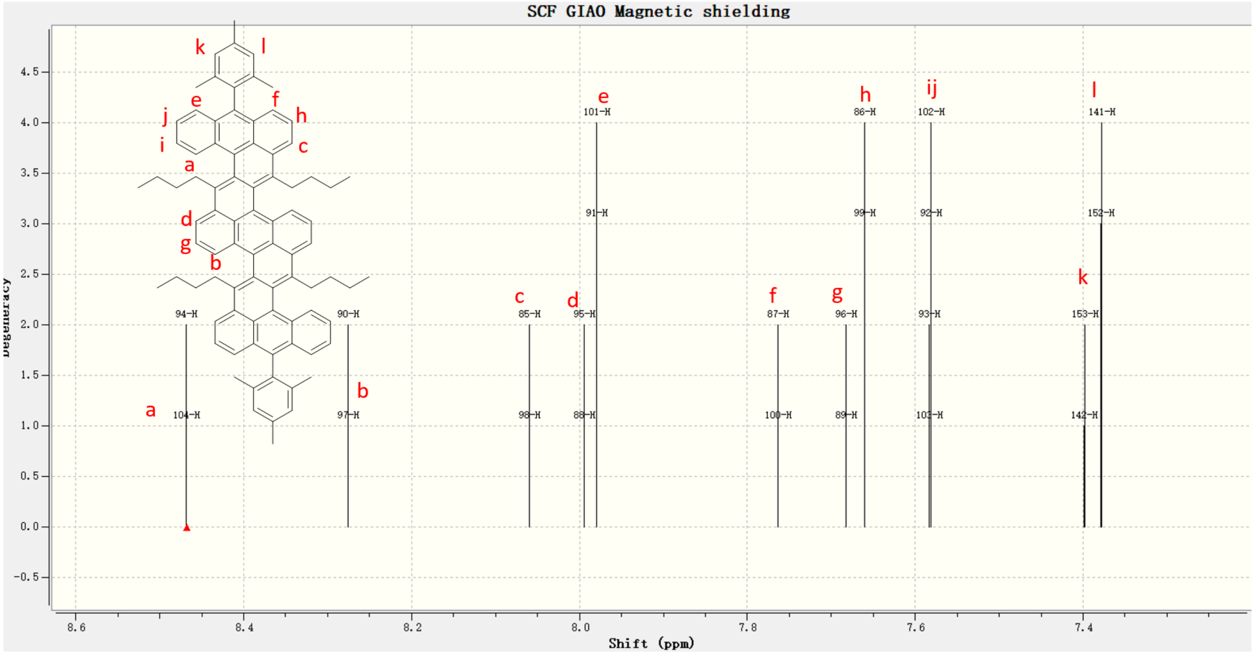

Figure S35. Calculated (B3LYP/6-31G(d,p)-GIAO) ^{1}H NMR spectrum of **2b**.

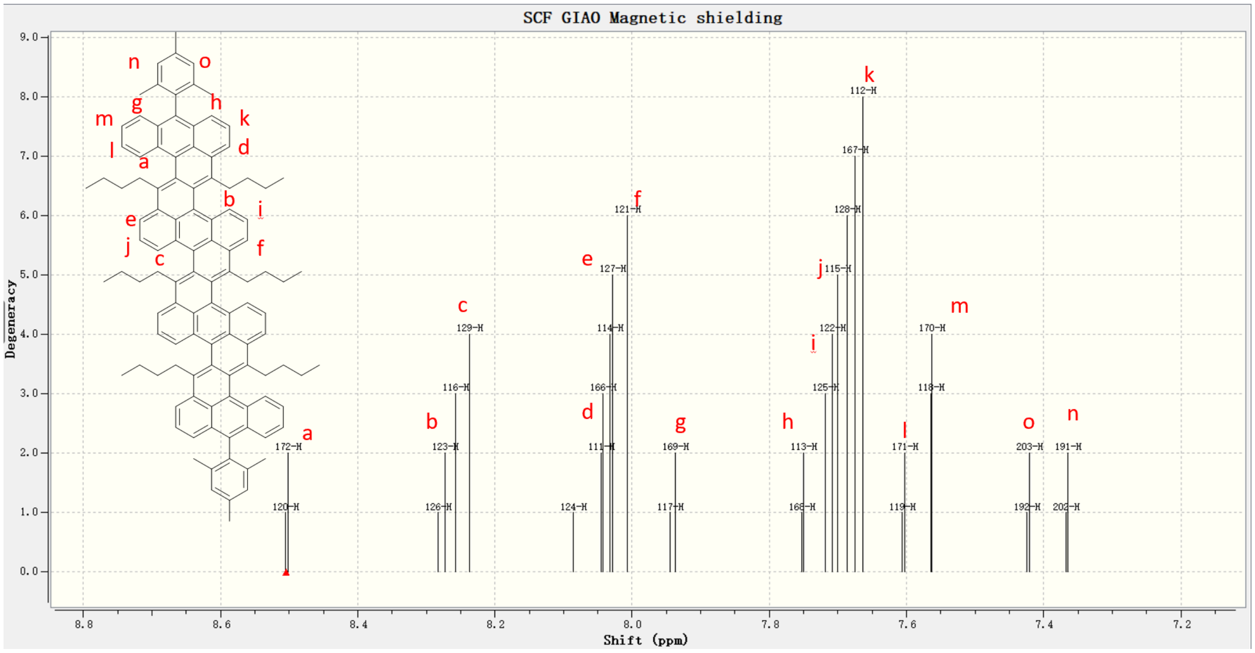

Figure S36. Calculated (B3LYP/6-31G(d,p)-GIAO) ^{1}H NMR spectrum of **3b**.

5. X-ray crystallographic data

Table S4. Crystal data and structure refinement for **1a** (Deposition Numbers 2248470).

Empirical formula	$C_{62.5}H_{47}Cl$
Formula weight	833.45
Temperature/K	104.0
Crystal system	triclinic
Space group	P-1
a/Å	14.1114(8)
b/Å	17.2949(10)
c/Å	19.1195(11)
α/°	92.959(2)
β/°	105.254(2)
γ/°	90.553(2)

Volume/Å^3	4494.5(4)
Z	4
ρ_{calc}g/cm^3	1.232
μ/mm^{-1}	1.058
F(000)	1756.0
Crystal size/mm^3	0.499 × 0.496 × 0.374
Radiation	CuKα (λ = 1.54178)
2Θ range for data collection/°	4.798 to 146.408
Index ranges	$-17 \le h \le 17$, $-18 \le k \le 21$, $-23 \le l \le 23$
Reflections collected	93144
Independent reflections	17143 [R_{int} = 0.0700, R_{sigma} = 0.0560]
Data/restraints/parameters	17143/0/1156
Goodness-of-fit on F^2	1.152
Final R indexes [I>=2σ (I)]	R_1 = 0.1218, wR_2 = 0.3018
Final R indexes [all data]	R_1 = 0.1296, wR_2 = 0.3038
Largest diff. peak/hole / e Å^{-3}	1.41/-1.08

Table S5. Crystal data and structure refinement for **1b** (Deposition Numbers 2248471).

Empirical formula	$C_{116}H_{108}$
Formula weight	1502.02
Temperature/K	100.0
Crystal system	monoclinic
Space group	C2/c
a/Å	37.1031(13)

b/Å	18.2874(6)
c/Å	30.6172(10)
α/°	90
β/°	123.328(2)
γ/°	90
Volume/$Å^3$	17357.8(11)
Z	8
ρ_{calc}g/cm^3	1.150
μ/mm^{-1}	0.484
F(000)	6432.0
Crystal size/mm^3	0.46 × 0.16 × 0.11
Radiation	CuKα (λ = 1.54178)
2Θ range for data collection/°	5.61 to 133.94
Index ranges	-43 ≤ h ≤ 44, -21 ≤ k ≤ 21, -36 ≤ l ≤ 32
Reflections collected	154547
Independent reflections	15370 [R_{int} = 0.1129, R_{sigma} = 0.0563]
Data/restraints/parameters	15370/0/1080
Goodness-of-fit on F^2	1.043
Final R indexes [I>=2σ (I)]	R_1 = 0.0562, wR_2 = 0.1372
Final R indexes [all data]	R_1 = 0.0681, wR_2 = 0.1453

Largest diff. peak/hole / e Å^{-3} 0.49/-0.36

Table S6. Crystal data and structure refinement for **2a** (Deposition Numbers 2248472).

Empirical formula	$C_{105.98}H_{77.98}$
Formula weight	1351.48
Temperature/K	100.00
Crystal system	triclinic
Space group	P-1
a/Å	12.6773(3)
b/Å	17.7433(4)
c/Å	18.0024(4)
α/°	72.5110(10)
β/°	77.9020(10)
γ/°	82.932(2)
Volume/Å^3	3768.61(15)
Z	2
ρ_{calc}g/cm^3	1.191
μ/mm^{-1}	0.508
F(000)	1428.0
Crystal size/mm^3	0.18 × 0.15 × 0.12
Radiation	CuKα (λ = 1.54178)
2Θ range for data collection/°	5.234 to 133.188
Index ranges	-15 ≤ h ≤ 15, -21 ≤ k ≤ 18, -21 ≤ l ≤ 21
Reflections collected	80370
Independent reflections	13163 [R_{int} = 0.0783, R_{sigma} = 0.0452]
Data/restraints/parameters	13163/1850/1392

Goodness-of-fit on F^2	1.045
Final R indexes [I>=2σ (I)]	R_1 = 0.0766, wR_2 = 0.1801
Final R indexes [all data]	R_1 = 0.1047, wR_2 = 0.2001
Largest diff. peak/hole / e $Å^{-3}$	0.44/-0.47

Table S7. Crystal data and structure refinement for **3b** (Deposition Numbers 2248473).

Empirical formula	$C_{138}H_{134}$
Formula weight	1792.44
Temperature/K	100.0
Crystal system	triclinic
Space group	P-1
a/Å	15.4947(8)
b/Å	17.9161(9)
c/Å	19.1436(10)
α/°	86.013(3)
β/°	86.561(3)
γ/°	73.993(3)
Volume/$Å^3$	5091.3(5)
Z	2
$\rho_{calc}g/cm^3$	1.169
μ/mm^{-1}	0.491
F(000)	1924.0
Crystal size/mm^3	0.22 × 0.12 × 0.07
Radiation	CuKα (λ = 1.54178)
2Θ range for data collection/°	5.14 to 133.714

Index ranges	$-18 \le h \le 17$, $-21 \le k \le 21$, $-22 \le l \le 19$
Reflections collected	83077
Independent reflections	17695 [R_{int} = 0.0531, R_{sigma} = 0.0484]
Data/restraints/parameters	17695/99/1300
Goodness-of-fit on F^2	1.043
Final R indexes [I>=2σ (I)]	R_1 = 0.0585, wR_2 = 0.1513
Final R indexes [all data]	R_1 = 0.0652, wR_2 = 0.1568
Largest diff. peak/hole / e Å^{-3}	0.45/-0.34

6. ^{1}H/^{13}C NMR spectra and HR MS spectra

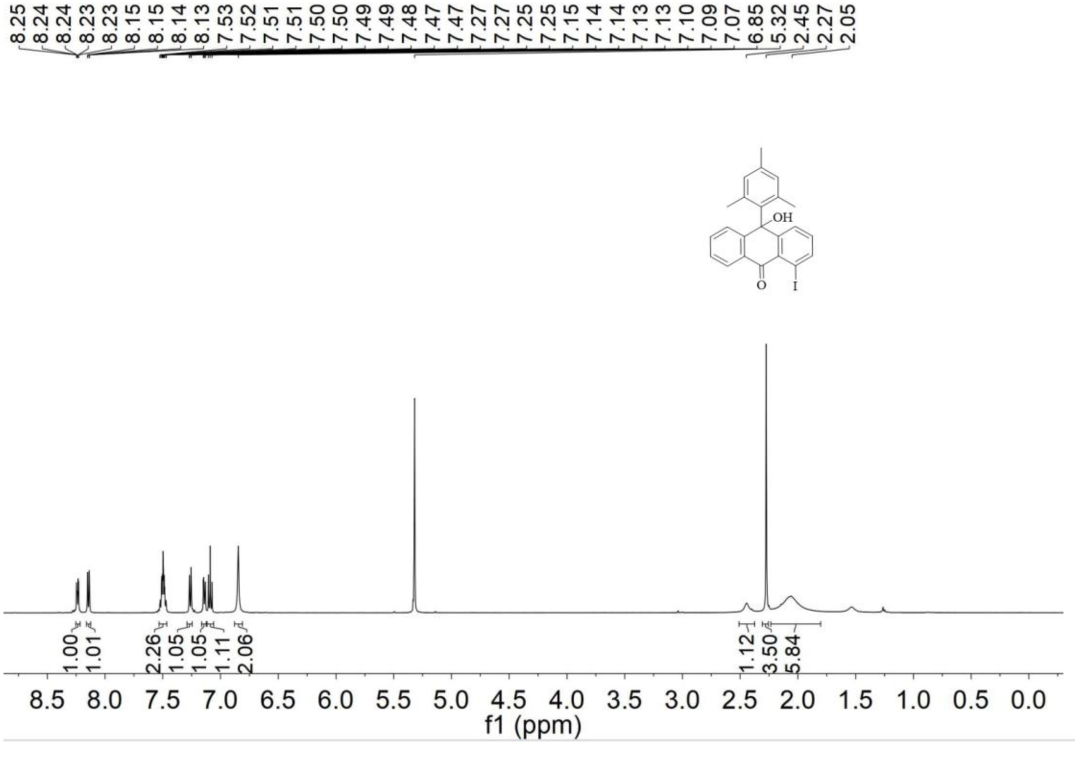

Figure S37. ^{1}H NMR spectrum of compound **4′** (500 MHz, CD_2Cl_2, rt).

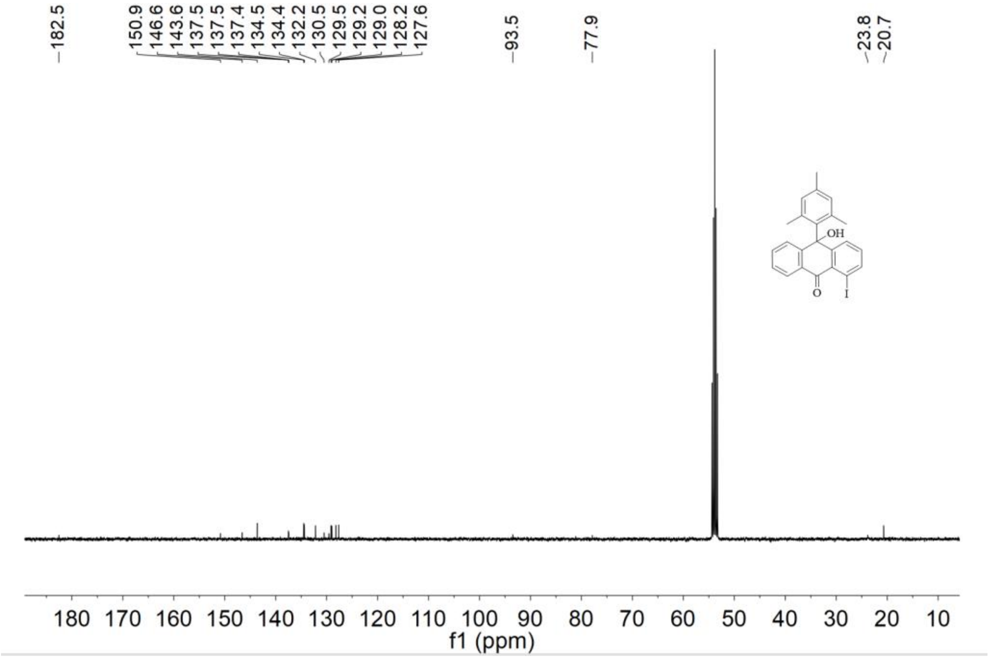

Figure S38. ^{13}C NMR spectrum of compound **4′** (100MHz, CD_2Cl_2, rt).

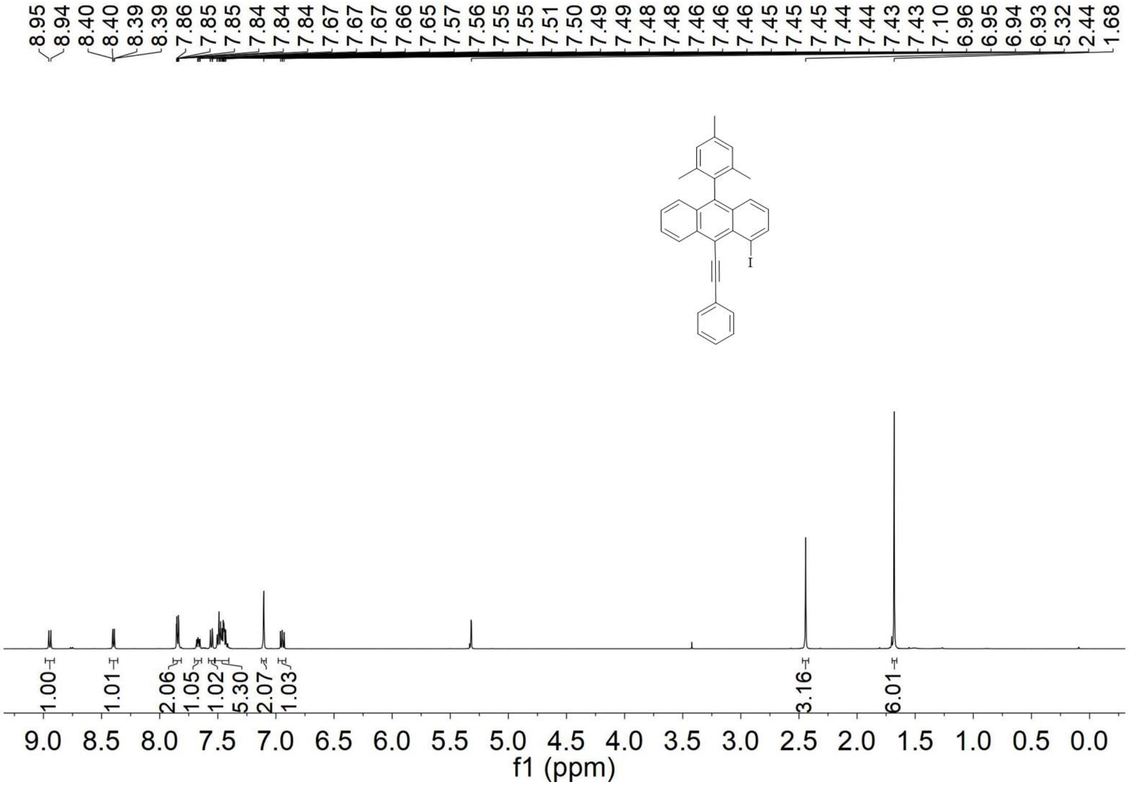

Figure S39. ^{1}H NMR spectrum of compound **5a** (500 MHz, CD_2Cl_2, rt).

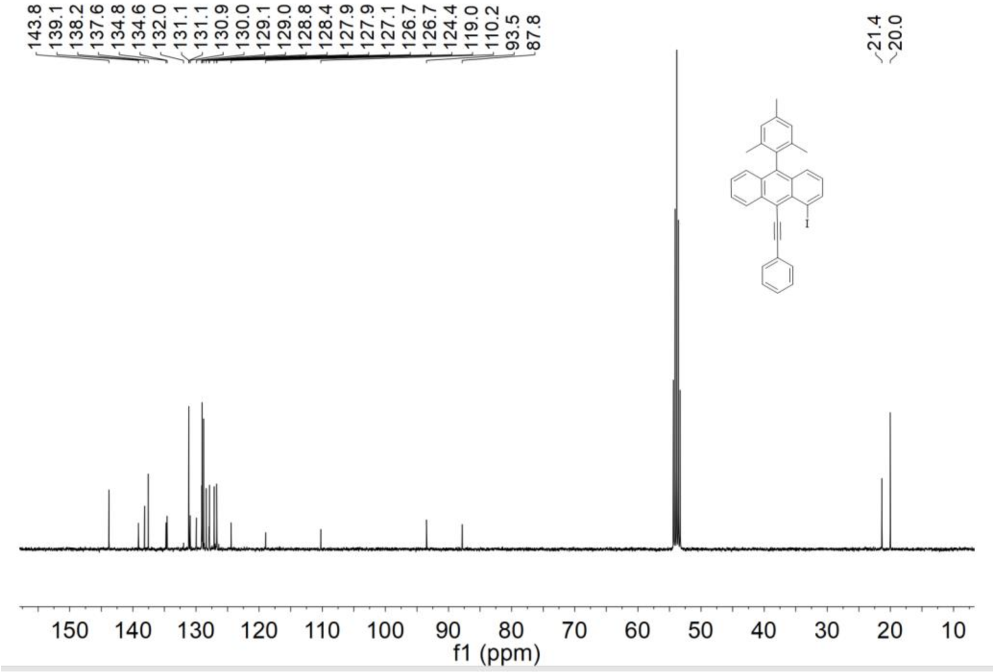

Figure S40. ^{13}C NMR spectrum of compound **5a** (100 MHz, CD_2Cl_2, rt).

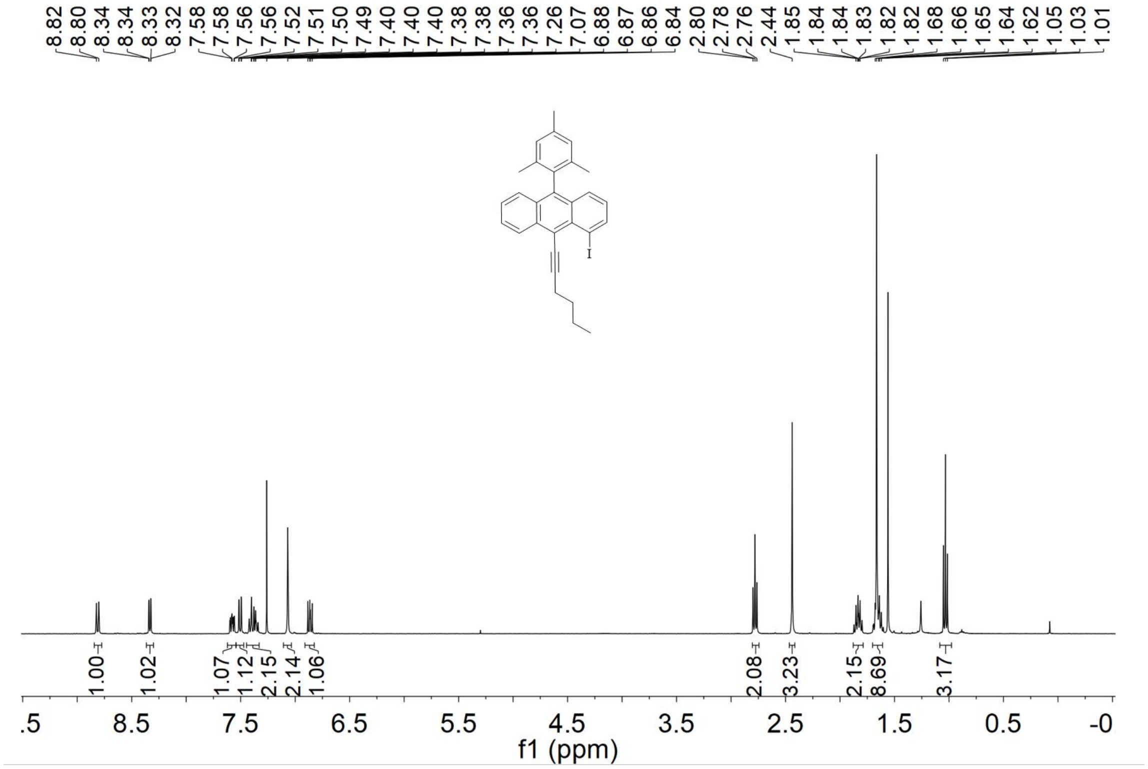

Figure S41. ^{1}H NMR spectrum of compound **5b** (500 MHz, $CDCl_3$, rt).

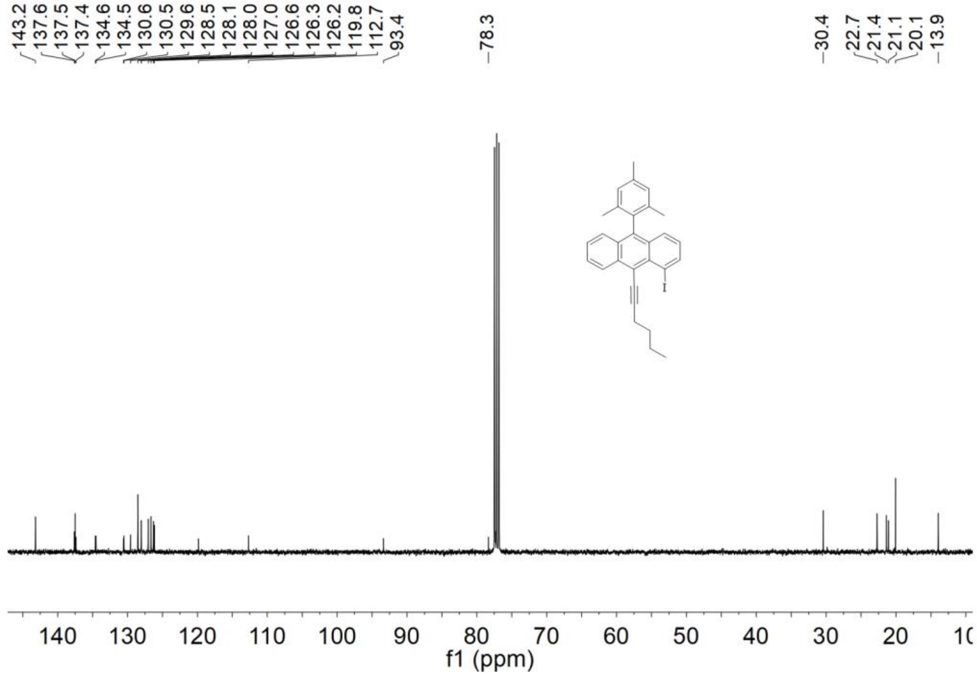

Figure S42. ^{13}C NMR spectrum of compound **5b** (100 MHz, $CDCl_3$, rt).

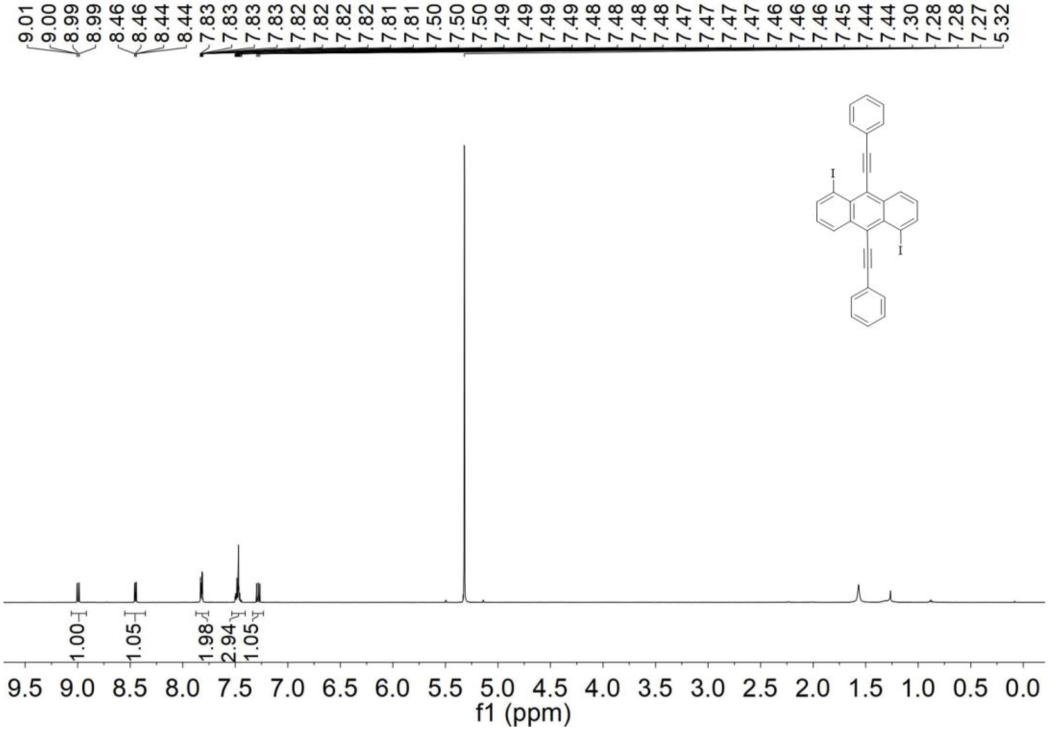

Figure S43. ^{1}H NMR spectrum of compound **7a** (500 MHz, CD_2Cl_2, rt).

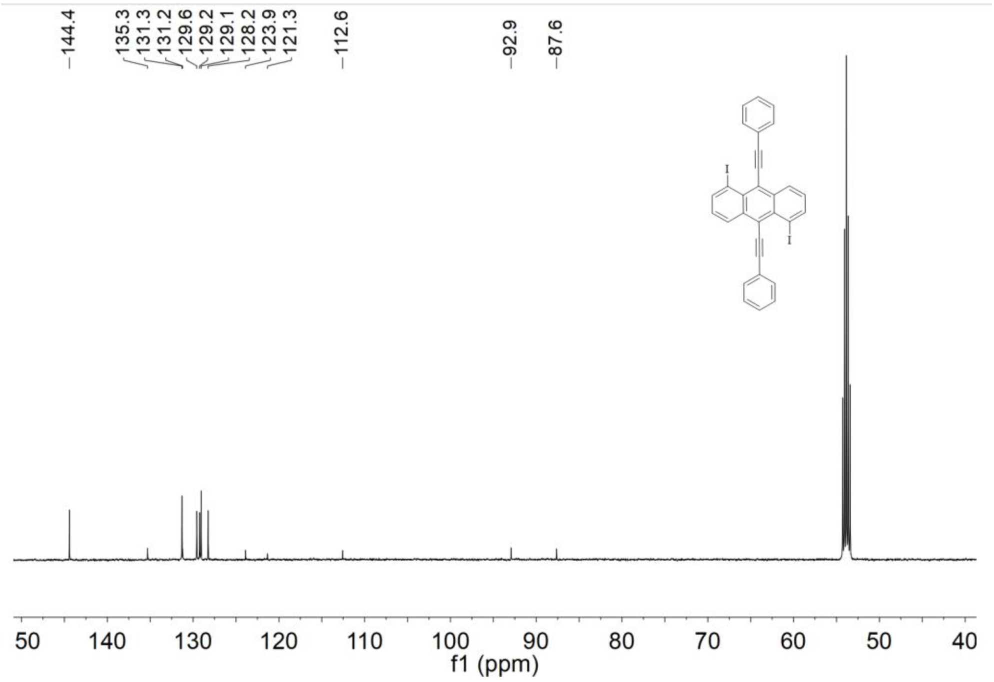

Figure S44. ^{13}C NMR spectrum of compound **7a** (125 MHz, CD_2Cl_2, rt).

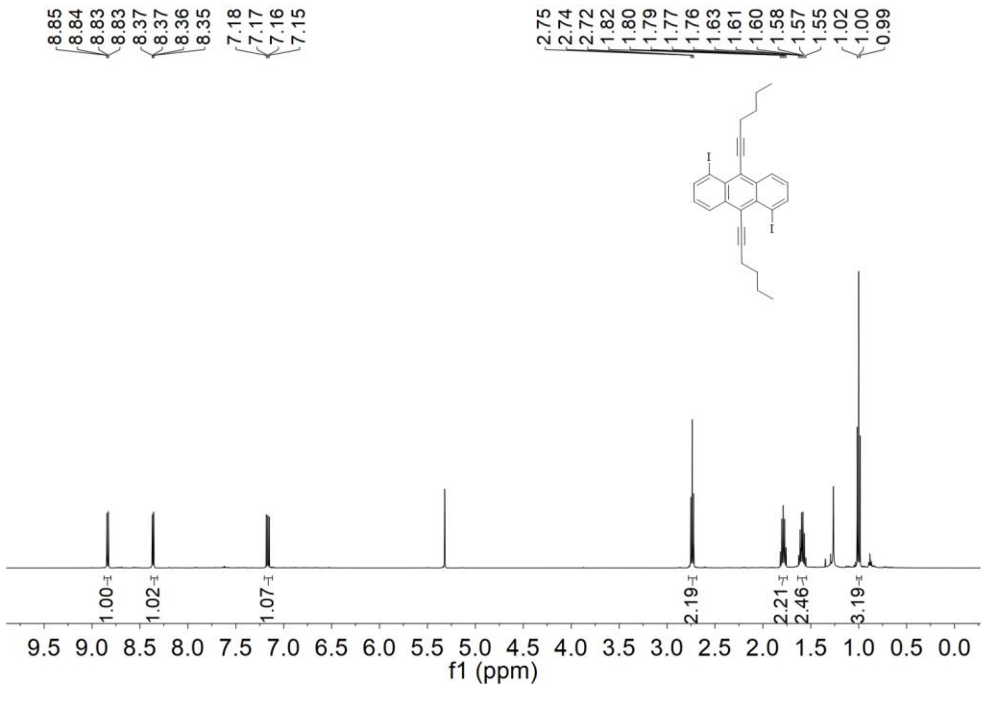

Figure S45. ^{1}H NMR spectrum of compound **7b** (500 MHz, CD_2Cl_2, rt).

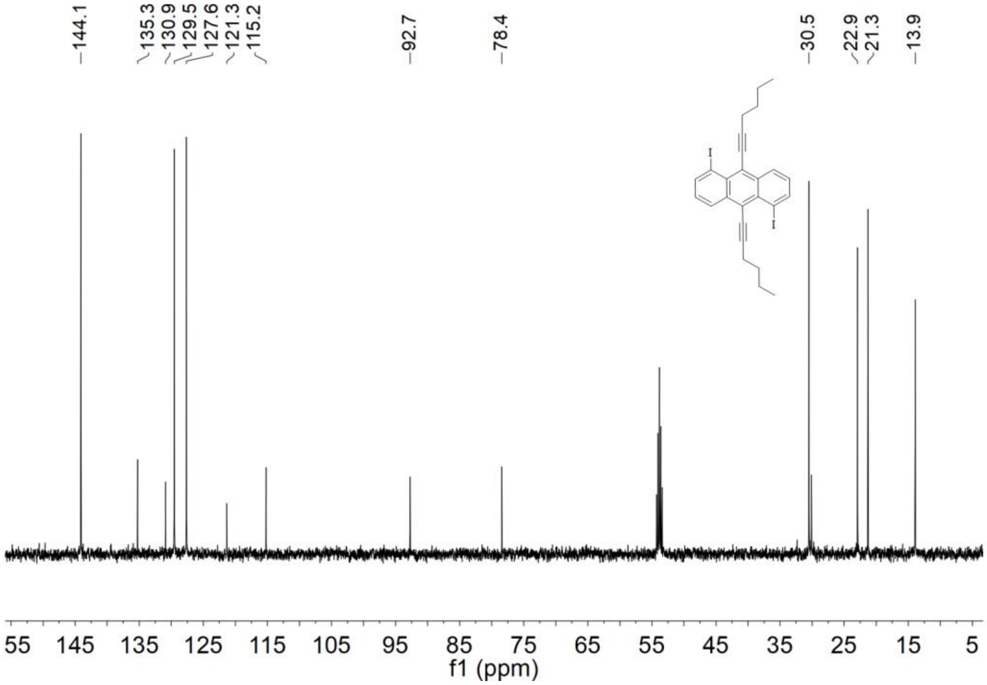

Figure S46. ^{13}C NMR spectrum of compound **7b** (125 MHz, CD_2Cl_2, rt).

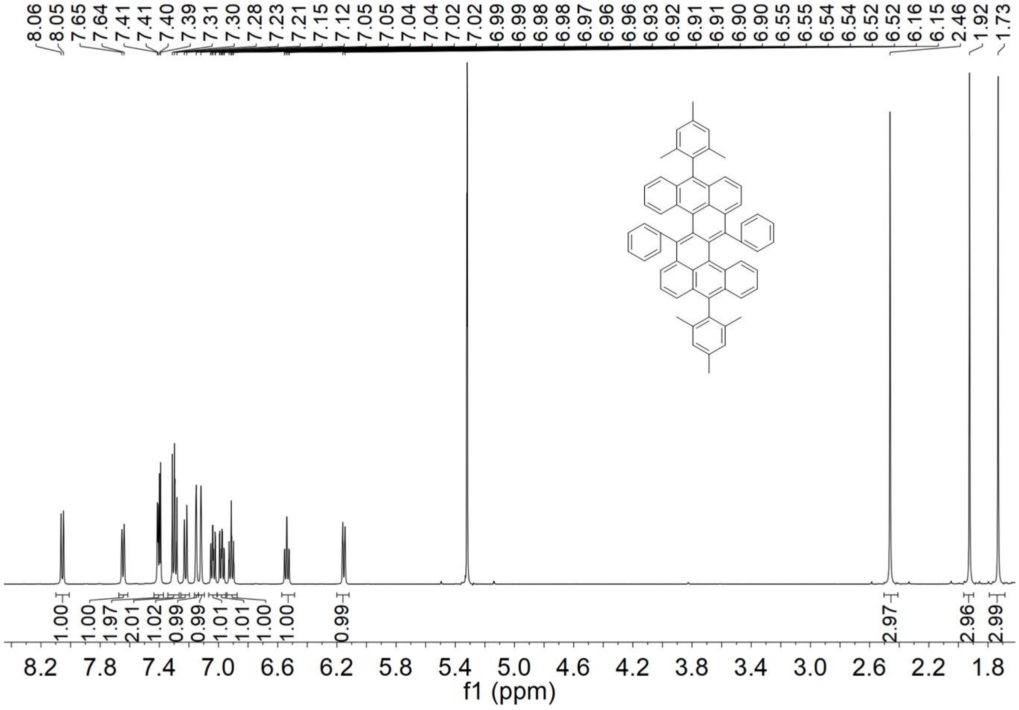

Figure S47. ^{1}H NMR spectrum of compound **1a** (500 MHz, deacidified CD_2Cl_2, rt).

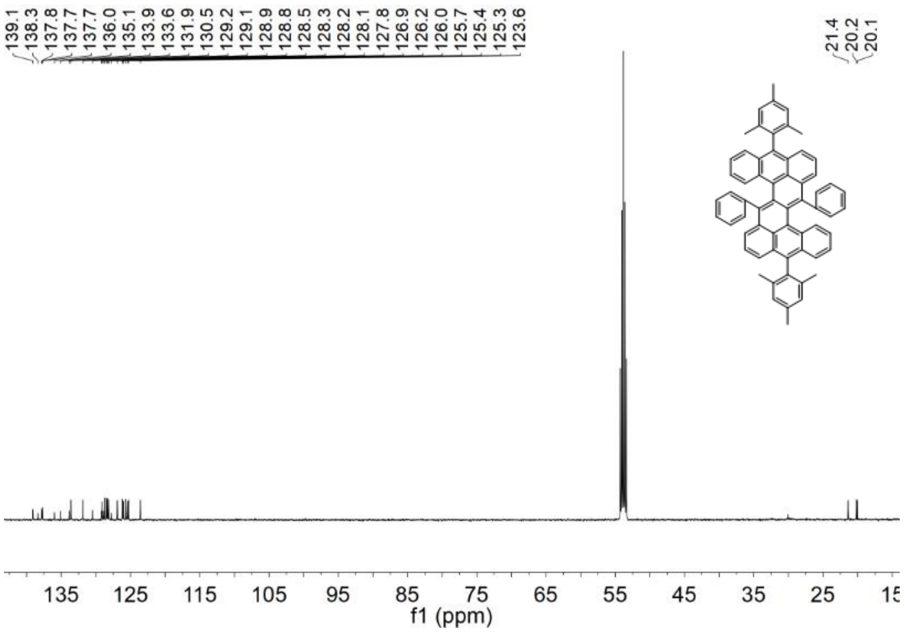

Figure S48. ^{13}C NMR spectrum of compound **1a** (125 MHz, deacidified CD_2Cl_2, rt).

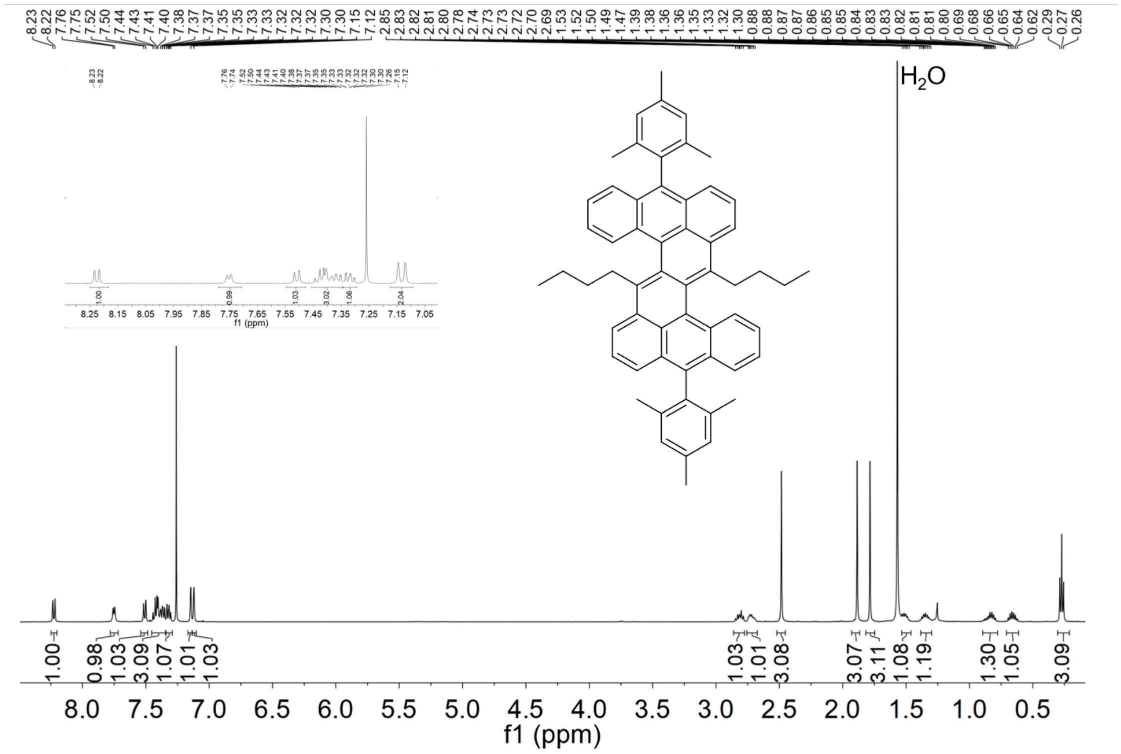

Figure S49. ^{1}H NMR spectrum of compound **1b** (500 MHz, deacidified $CDCl_3$, rt).

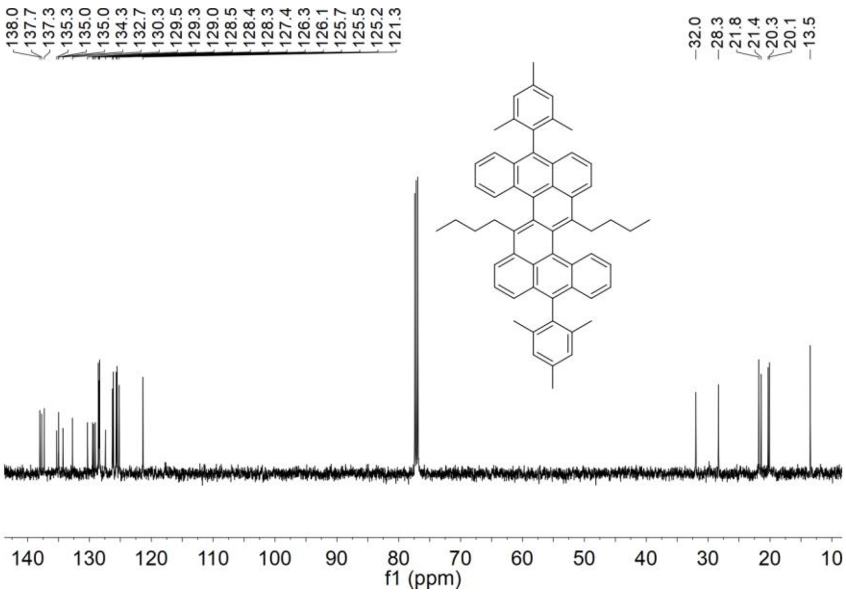

Figure S50. ^{13}C NMR spectrum of compound **1b** (125 MHz, deacidified $CDCl_3$, rt).

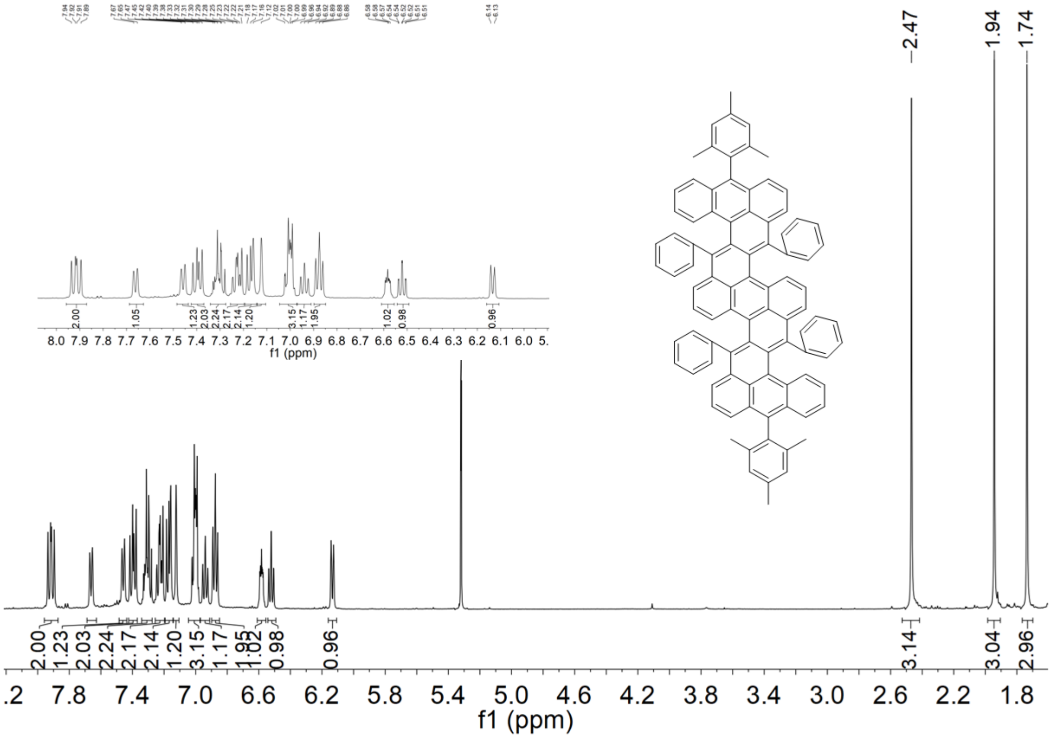

Figure S51. ^{1}H NMR spectrum of compound **2a** (500 MHz, deacidified CD_2Cl_2, rt).

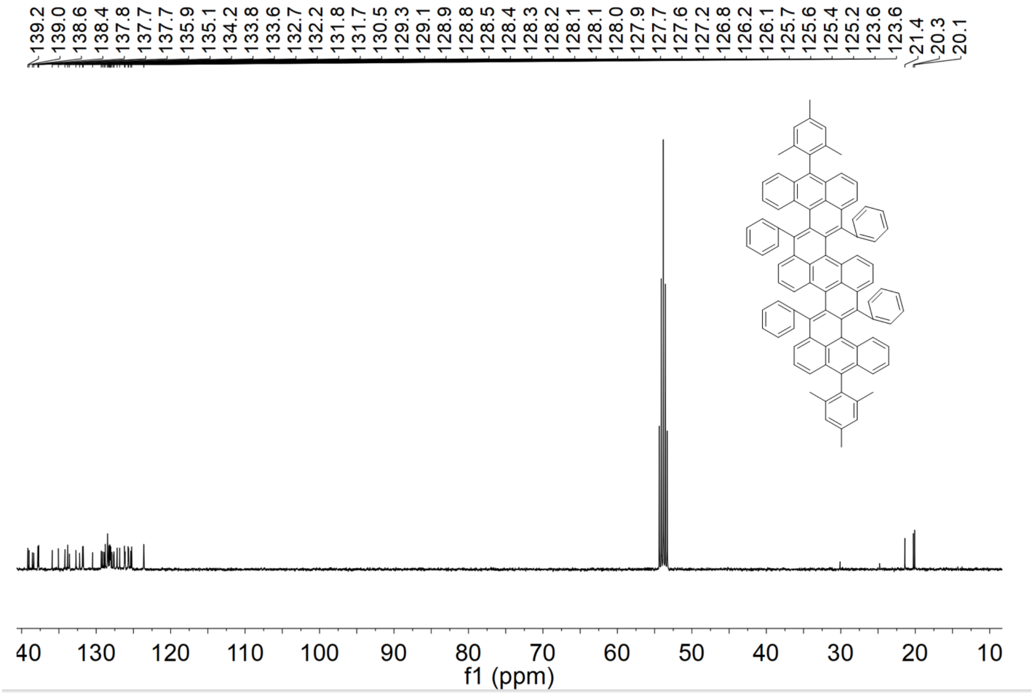

Figure S52. ^{13}C NMR spectrum of compound **2a** (100 MHz, deacidified CD_2Cl_2, rt).

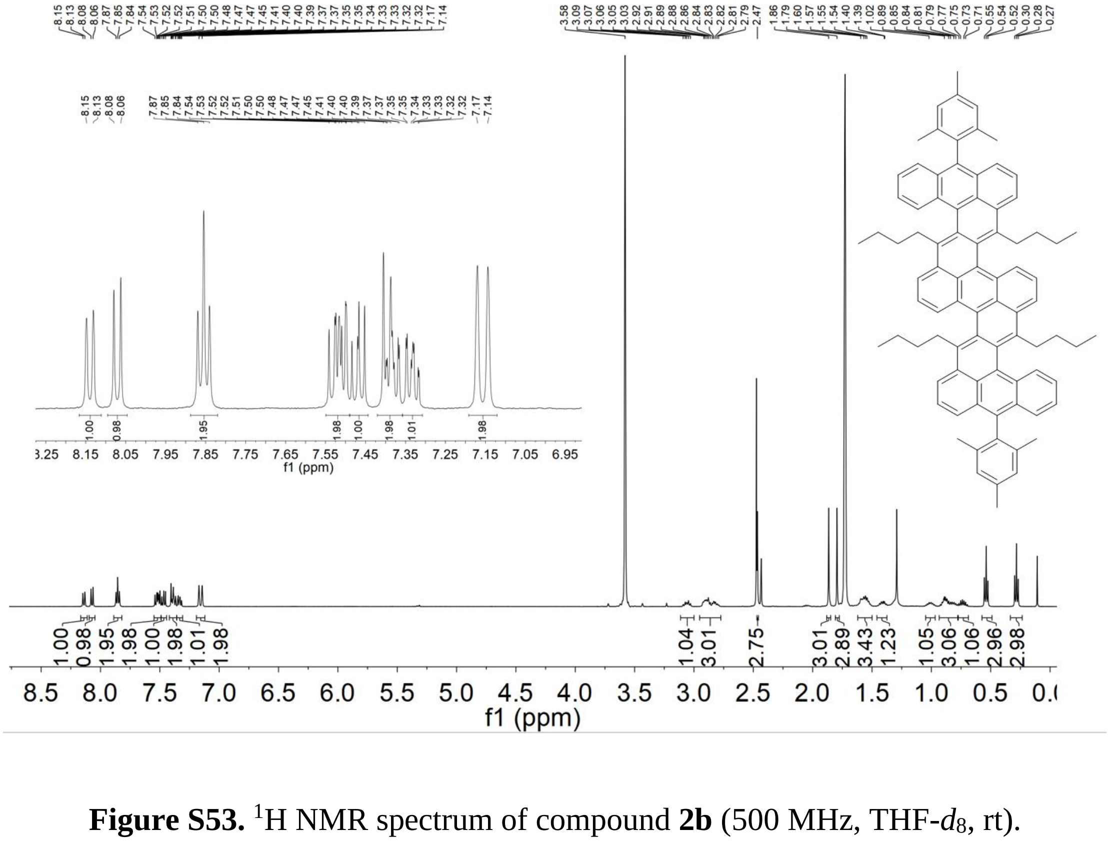

Figure S53. ^{1}H NMR spectrum of compound **2b** (500 MHz, THF-d_8, rt).

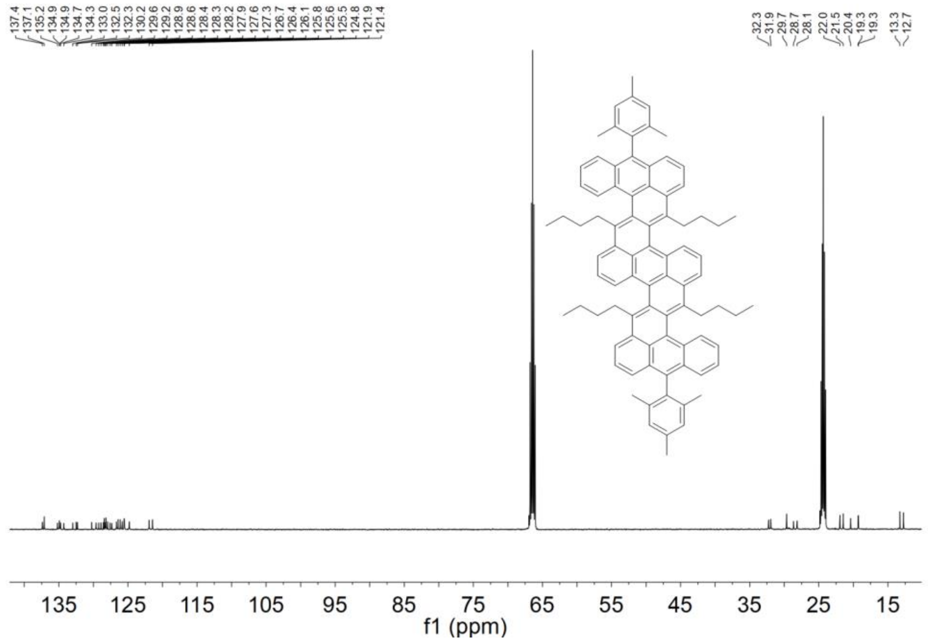

Figure S54. ^{13}C NMR spectrum of compound **2b** (125 MHz, THF-d_8, rt).

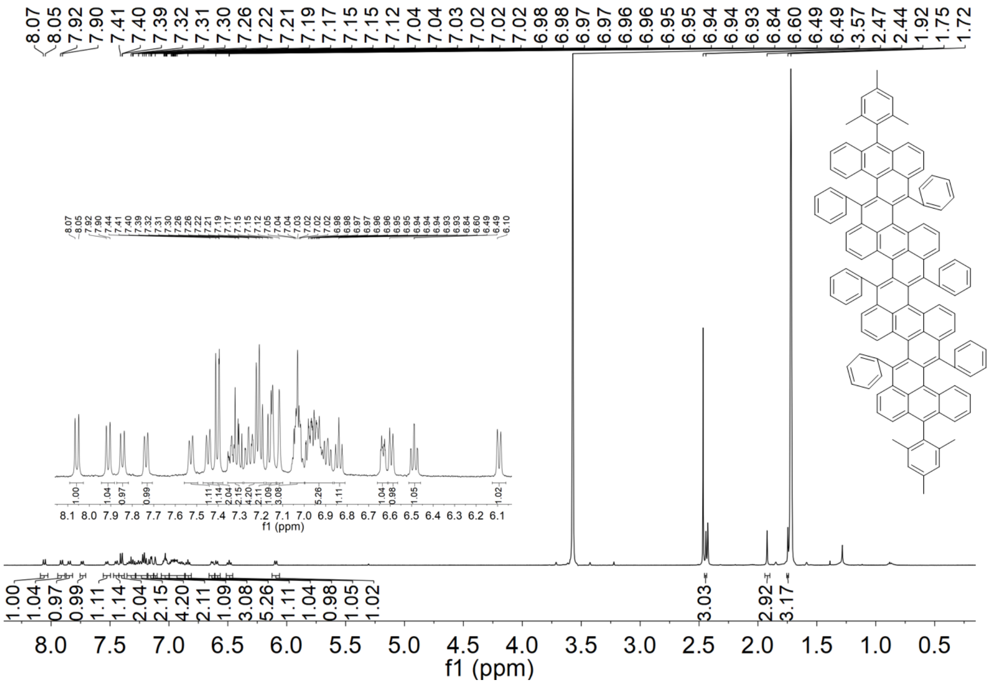

Figure S55. ^{1}H NMR spectrum of compound **3a** (500 MHz, THF-d_8, rt).

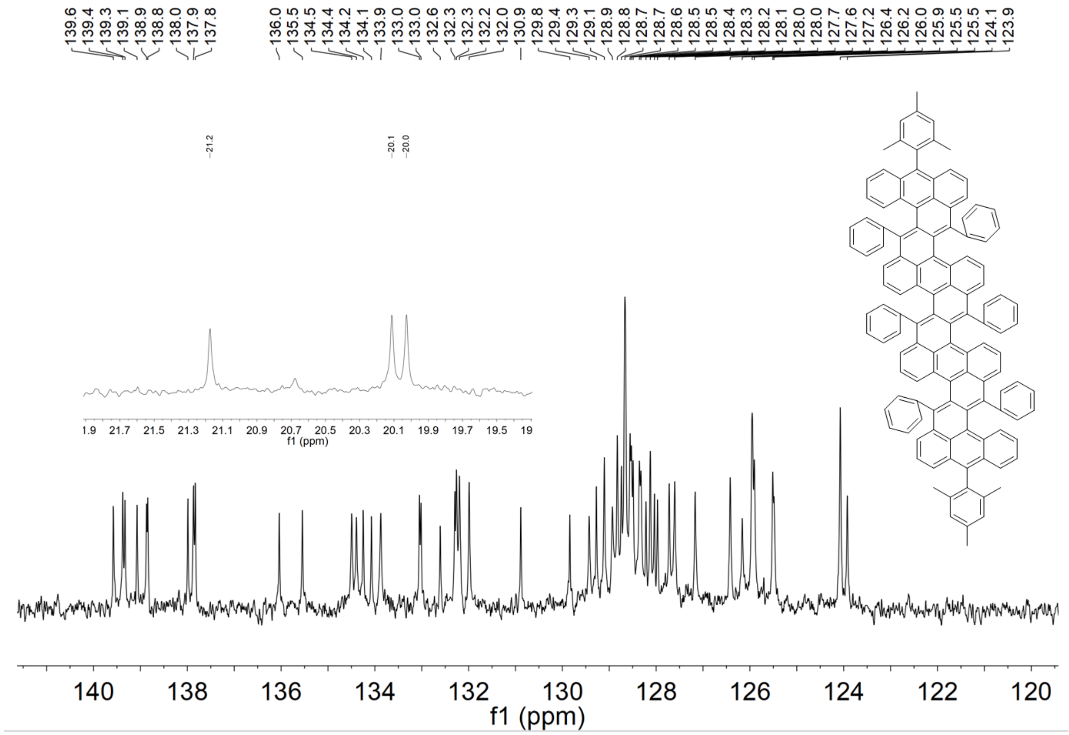

Figure S56. ^{13}C NMR spectrum of compound **3a** (125 MHz, THF-d_8, rt).

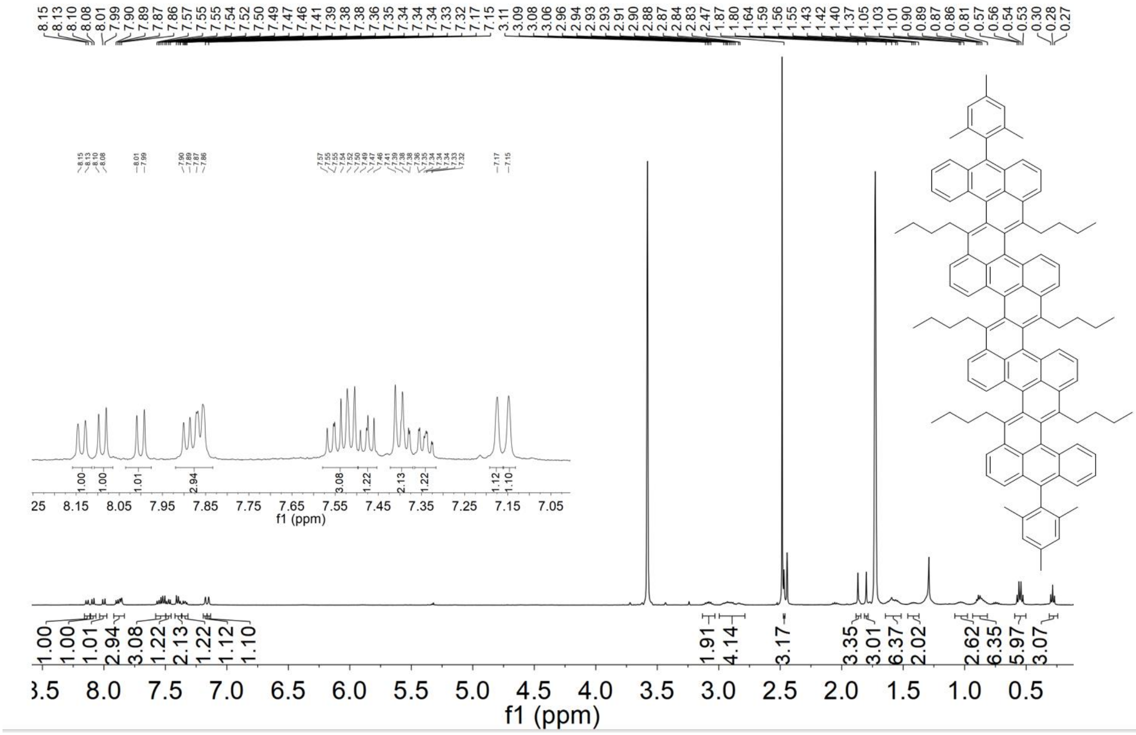

Figure S57. ^{1}H NMR spectrum of compound **3b** (500 MHz, THF-d_8, rt).

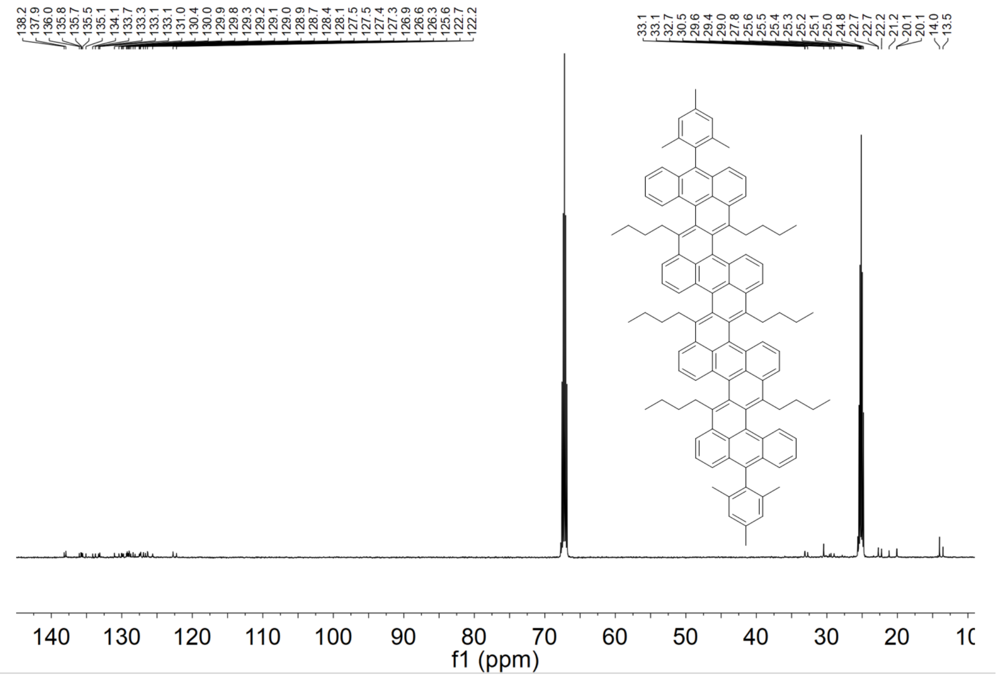

Figure S58. ^{13}C NMR spectrum of compound **3b** (125 MHz, THF-d_8, rt).

Mass Spectrum SmartFormula Report

**Sample Name**	SZT-1	**Data File**	D:\MassHunter\Data\Chemistry\2023\202301\20230103\SZT-1.d
**Instrument Name**	Agilent 6546 LC-QTOF	**IRM Calibration Status**	All Ions Missed
**Acq Method**	MS Scan_union_APCI-4.m	**Acquired Time**	3/1/2023 3:16:49 PM (UTC+08:00)
**Comment**	Prof Wu Jishan	**Operator**	WLK

Meas. m/z	#	Formula	Calc. Mass	Err [ppm]
455.0509	1	C23 H20 I O2	455.0502	1.54

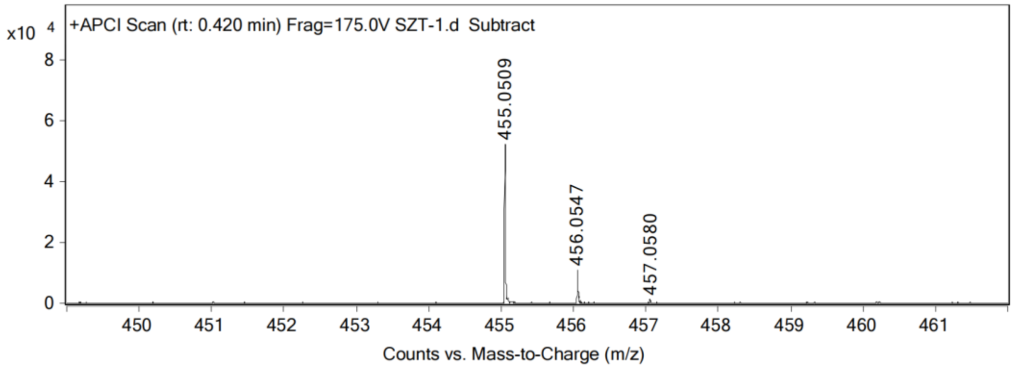

Page 1 of 1 Printed at 4:02 PM on 3-Jan-2023

Figure S59. HR mass spectrum (APCI) of **4′**.

Mass Spectrum SmartFormula Report

**Sample Name**	SZT-2	**Data File**	D:\MassHunter\Data\Chemistry\2023\202301\20230103\SZT-2.d
**Instrument Name**	Agilent 6546 LC-QTOF	**IRM Calibration Status**	All Ions Missed
**Acq Method**	MS Scan_union_APCI-4.m	**Acquired Time**	3/1/2023 3:19:50 PM (UTC+08:00)
**Comment**	Prof Wu Jishan	**Operator**	WLK

Meas. m/z	#	Formula	Calc. Mass	Err [ppm]
523.092	1	C31 H24 I	523.0917	0.57

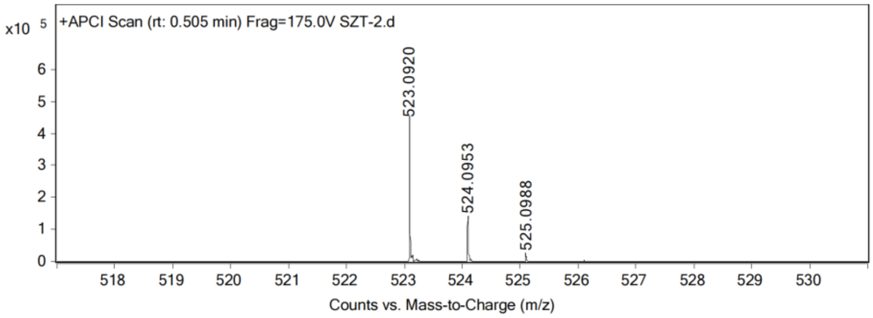

Page 1 of 1 Printed at 4:03 PM on 3-Jan-2023

Figure S60. HR mass spectrum (APCI) of **5a**.

Mass Spectrum SmartFormula Report

**Sample Name**	Szt-1	**Data File**	D:\MassHunter\Data\Chemistry\2023\202302\20230214\Szt-1.d
**Instrument Name**	Agilent 6546 LC-QTOF	**IRM Calibration Status**	All Ions Missed
**Acq Method**	MS Scan_union_APCI-3.m	**Acquired Time**	14/2/2023 5:12:26 PM (UTC+08:00)
**Comment**	Prof Wu Jishan	**Operator**	WLK

Meas. m/z	#	Formula	Calc. Mass	Err [ppm]
503.1227	1	C29 H28 I	503.123	0.60

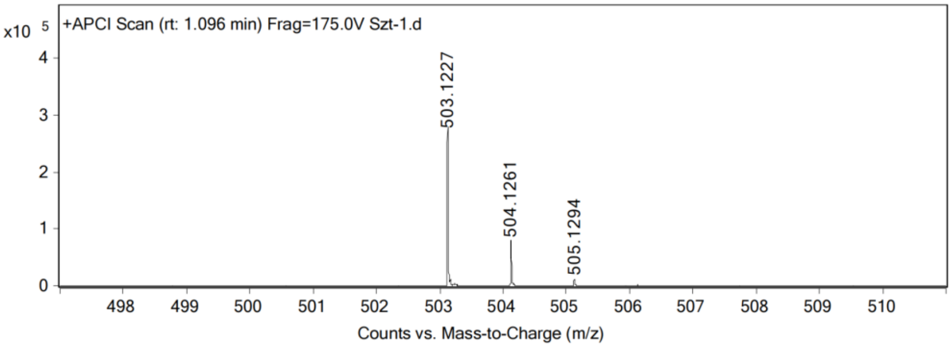

Page 1 of 1 Printed at 5:19 PM on 14-Feb-2023

Figure S61. HR mass spectrum (APCI) of **5b**.

Mass Spectrum SmartFormula Report

**Sample Name**	SZT-3	**Data File**	D:\MassHunter\Data\Chemistry\2023\202301\20230103\SZT-3.d
**Instrument Name**	Agilent 6546 LC-QTOF	**IRM Calibration Status**	Some Ions Missed
**Acq Method**	MS Scan_union_APCI-4.m	**Acquired Time**	3/1/2023 3:22:00 PM (UTC+08:00)
**Comment**	Prof Wu Jishan	**Operator**	WLK

Meas. m/z	#	Formula	Calc. Mass	Err [ppm]
630.9411	1	C30 H17 I2	630.9414	0.48

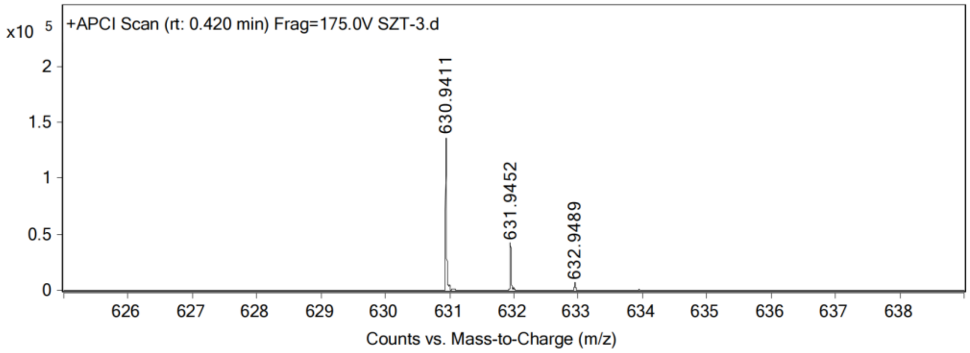

Page 1 of 1 Printed at 4:05 PM on 3-Jan-2023

Figure S62. HR mass spectrum (APCI) of **7a**.

Mass Spectrum SmartFormula Report

**Sample Name**	Szt-2	**Data File**	D:\MassHunter\Data\Chemistry\2023\202302\20230214\Szt-2.d
**Instrument Name**	Agilent 6546 LC-QTOF	**IRM Calibration Status**	All Ions Missed
**Acq Method**	MS Scan_union_APCI-3.m	**Acquired Time**	14/2/2023 5:17:17 PM (UTC+08:00)
**Comment**	Prof Wu Jishan	**Operator**	WLK

Meas. m/z	#	Formula	Calc. Mass	Err [ppm]
591.0036	1	C26 H25 I2	591.004	0.68

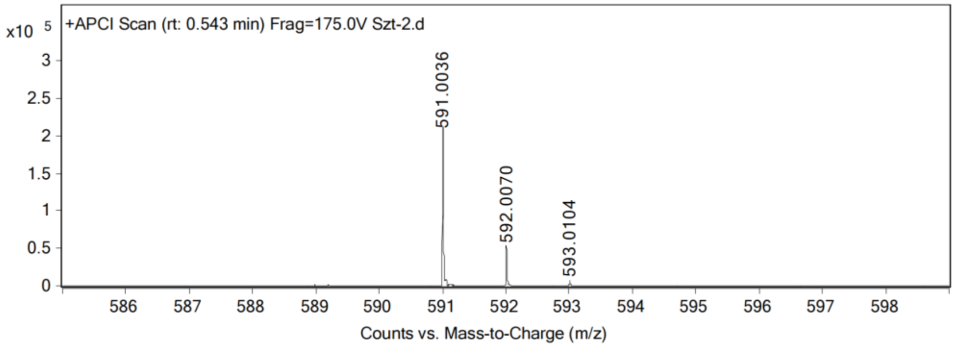

Page 1 of 1 Printed at 5:27 PM on 14-Feb-2023

Figure S63. HR mass spectrum (APCI) of **7b**.

Mass Spectrum SmartFormula Report

**Sample Name**	SZT-4	**Data File**	D:\MassHunter\Data\Chemistry\2023\202301\20230103\SZT-4.d
**Instrument Name**	Agilent 6546 LC-QTOF	**IRM Calibration Status**	All Ions Missed
**Acq Method**	MS Scan_union_APCI-4.m	**Acquired Time**	3/1/2023 3:24:34 PM (UTC+08:00)
**Comment**	Prof Wu Jishan	**Operator**	WLK

**Meas. m/z**	**#**	**Formula**	**Calc. Mass**	**Err [ppm]**
791.3668	1	C62 H47	791.3672	0.51

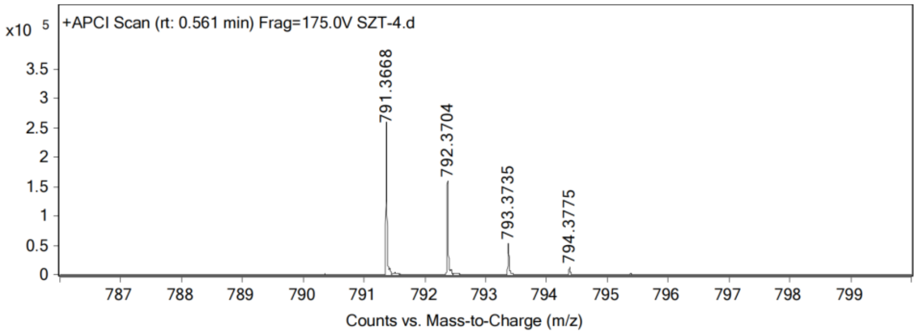

Page 1 of 1 Printed at 4:07 PM on 3-Jan-2023

Figure S64. HR mass spectrum (APCI) of **1a**.

Mass Spectrum SmartFormula Report

**Sample Name**	Szt-3	**Data File**	D:\MassHunter\Data\Chemistry\2023\202302\20230214\Szt-3.d
**Instrument Name**	Agilent 6546 LC-QTOF	**IRM Calibration Status**	Some Ions Missed
**Acq Method**	MS Scan_union_APCI-3.m	**Acquired Time**	14/2/2023 5:19:58 PM (UTC+08:00)
**Comment**	Prof Wu Jishan	**Operator**	WLK

**Meas. m/z**	**#**	**Formula**	**Calc. Mass**	**Err [ppm]**
751.43	1	C58 H55	751.4298	0.27

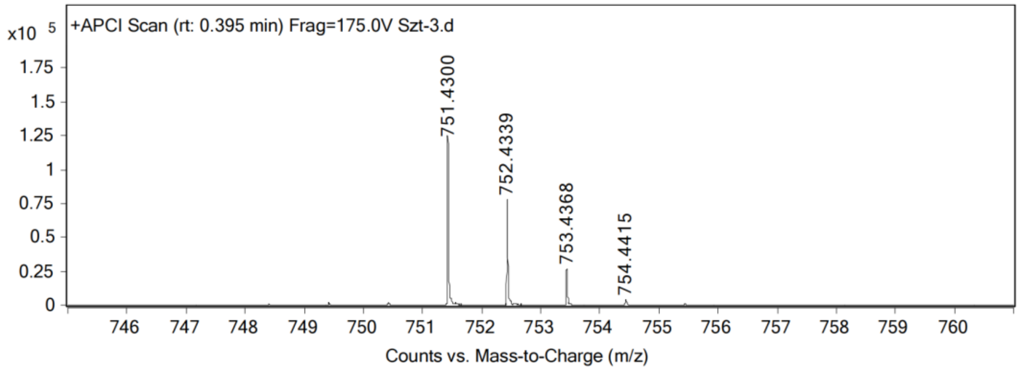

Page 1 of 1 Printed at 5:29 PM on 14-Feb-2023

Figure S65. HR mass spectrum (APCI) of **1b**.

Mass Spectrum SmartFormula Report

**Sample Name**	SZT-5	**Data File**	D:\MassHunter\Data\Chemistry\2023\202301\20230103-apci\SZT-5.d
**Instrument Name**	Agilent 6546 LC-QTOF	**IRM Calibration Status**	Some Ions Missed
**Acq Method**	MS Scan_union_APCI-4.m	**Acquired Time**	3/1/2023 3:27:52 PM (UTC+08:00)
**Comment**	Prof Wu Jishan	**Operator**	WLK

Meas. m/z	#	Formula	Calc. Mass	Err [ppm]
1167.4922	1	C92 H63	1167.4924	0.17

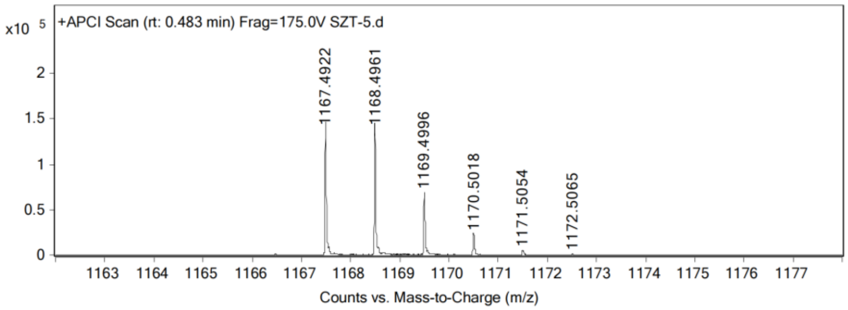

Page 1 of 1 Printed at 4:25 PM on 3-Jan-2023

Figure S66. HR mass spectrum (APCI) of **2a**.

Mass Spectrum SmartFormula Report

**Sample Name**	Szt-4	**Data File**	D:\MassHunter\Data\Chemistry\2023\202302\20230214\Szt-4.d
**Instrument Name**	Agilent 6546 LC-QTOF	**IRM Calibration Status**	Some Ions Missed
**Acq Method**	MS Scan_union_APCI-3.m	**Acquired Time**	14/2/2023 5:22:29 PM (UTC+08:00)
**Comment**	Prof Wu Jishan	**Operator**	WLK

Meas. m/z	#	Formula	Calc. Mass	Err [ppm]
1087.6184	1	C84 H79	1087.6176	0.74

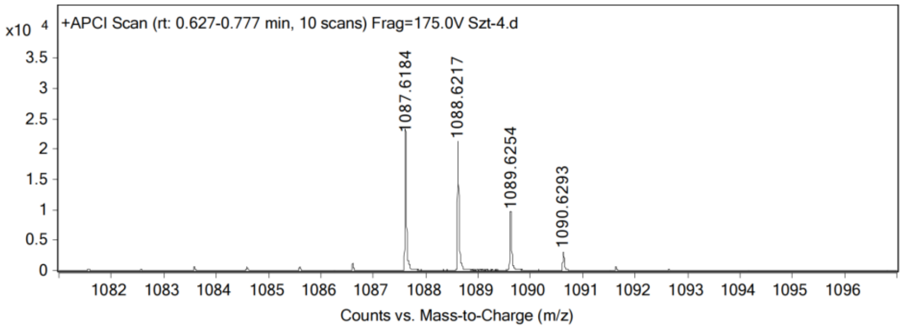

Page 1 of 1 Printed at 5:35 PM on 14-Feb-2023

Figure S67. HR mass spectrum (APCI) of **2b**.

Mass Spectrum SmartFormula Report

Sample Name	SZT-6	Data File	D:\MassHunter\Data\Chemistry\2023\202301\20230103-apci\SZT-6.d
Instrument Name	Agilent 6546 LC-QTOF	IRM Calibration Status	Some Ions Missed
Acq Method	MS Scan_union_APCI-4.m	Acquired Time	3/1/2023 3:30:23 PM (UTC+08:00)
Comment	Prof Wu Jishan	Operator	WLK

Meas. m/z	#	Formula	Calc. Mass	Err [ppm]
1543.6175	1	C122 H79	1543.6176	0.06

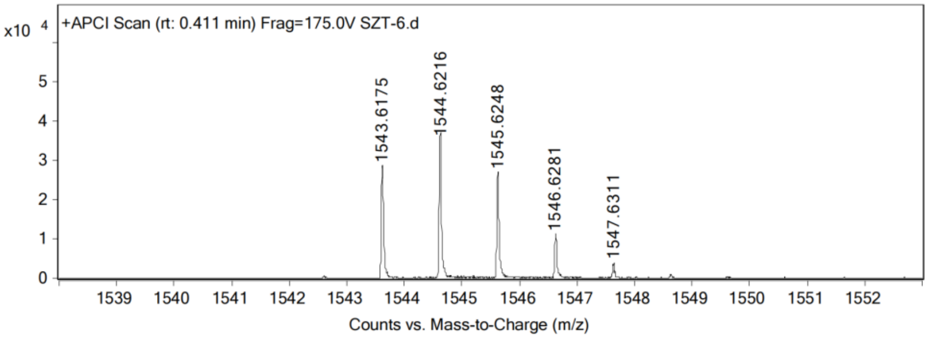

Page 1 of 1 Printed at 4:26 PM on 3-Jan-2023

Figure S68. HR mass spectrum (APCI) of **3a**.

Mass Spectrum SmartFormula Report

Sample Name	Szt-5	Data File	D:\MassHunter\Data\Chemistry\2023\202302\20230214\Szt-5.d
Instrument Name	Agilent 6546 LC-QTOF	IRM Calibration Status	Some Ions Missed
Acq Method	MS Scan_union_APCI-3.m	Acquired Time	14/2/2023 5:25:01 PM (UTC+08:00)
Comment	Prof Wu Jishan	Operator	WLK

Meas. m/z	#	Formula	Calc. Mass	Err [ppm]
1423.8044	1	C110 H103	1423.8054	0.70

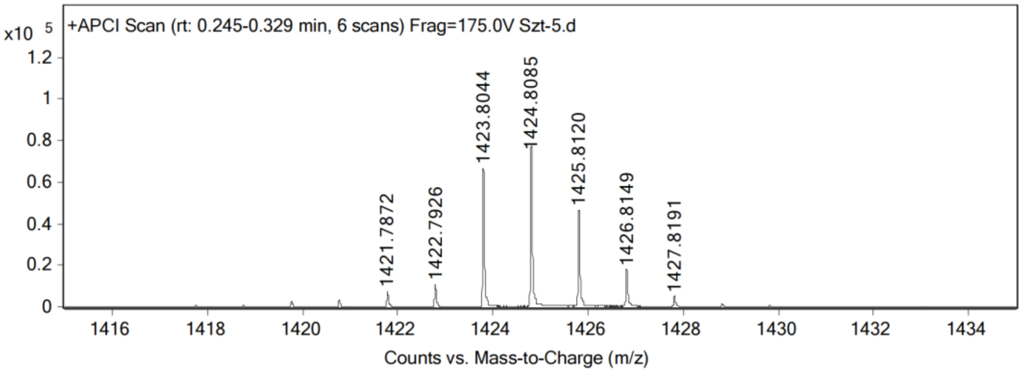

Page 1 of 1 Printed at 5:37 PM on 14-Feb-2023

Figure S69. HR mass spectrum (APCI) of **3b**.

7. References

[1] J. K. Kendall, H. Shechter, *J. Org. Chem.* **2001**, *66*, 6643-6649.

[2] R. H. Martin, M. J. Marchant, *Tetrahedron* **1974**, *30*, 343–345.

[3] Gaussian 09, Revision D.01, M. J. Frisch, G. W. Trucks, H. B. Schlegel, G. E. Scuseria, M. A. Robb, J. R. Cheeseman, G. Scalmani, V. Barone, B. Mennucci, G. A. Petersson, H. Nakatsuji, M. Caricato, X. Li, H. P. Hratchian, A. F. Izmaylov, J. Bloino, G. Zheng, J. L. Sonnenberg, M. Hada, M. Ehara, K. Toyota, R. Fukuda, J. Hasegawa, M. Ishida, T. Nakajima, Y. Honda, O. Kitao, H. Nakai, T. Vreven, J. A. Montgomery, Jr., J. E. Peralta, F. Ogliaro, M. Bearpark, J. J. Heyd, E. Brothers, K. N. Kudin, V. N. Staroverov, T. Keith, R. Kobayashi, J. Normand, K. Raghavachari, A. Rendell, J. C. Burant, S. S. Iyengar, J. Tomasi, M. Cossi, N. Rega, J. M. Millam, M. Klene, J. E. Knox, J. B. Cross, V. Bakken, C. Adamo, J. Jaramillo, R. Gomperts, R. E. Stratmann, O. Yazyev, A. J. Austin, R. Cammi, C. Pomelli, J. W. Ochterski, R. L. Martin, K. Morokuma, V. G. Zakrzewski, G. A. Voth, P. Salvador, J. J. Dannenberg, S. Dapprich, A. D. Daniels, O. Farkas, J. B. Foresman, J. V. Ortiz, J. Cioslowski, and D. J. Fox, Gaussian, Inc., Wallingford CT, **2013**.

[4] (a) A. D. Becke, *J. Chem. Phys.* **1993**, *98*, 5648-5652; (b) C. Lee, W. Yang, R. G. Parr, *Phys. Rev. B: Condens. Matter* **1988**, *37*, 785-789; (c) T. Yanai, D. Tew, N. Handy, *Chem. Phys. Lett.* **2004**, *393*, 51-57; (d) R. Ditchfield, J. W. Hehre, J. A. Pople, *J. Chem. Phys.* **1971**, *54*, 724-728; (e) W. J. Hehre, R. Ditchfield, J. A. Pople, *J. Chem. Phys.* **1972**, *56*, 2257-2261; (f) P. C. Hariharan, J. A. Pople, *Theor. Chim. Acta* **1973**, *28*, 213-222.

[5] P. V. R. Schleyer, C. Maerker, A. Dransfeld, H. Jiao, N. J. R. van Eikema Hommes, *J. Am. Chem. Soc.* **1996**, *118*, 6317–6318.

[6] D. Geuenich, K. Hess, F. Kohler, R. Herges, *Chem. Rev.* **2005**, *105*, 3758–3772.
